# Development of Age‐ and Sex‐Specific Metabolomics‐Based Biological Ageing Clocks for 10‐Year Mortality Prediction

**DOI:** 10.1002/advs.202510189

**Published:** 2025-10-15

**Authors:** Lei Peng, Ruijie Xie, Bernd Holleczek, Hermann Brenner, Ben Schöttker

**Affiliations:** ^1^ Division of Clinical Epidemiology and Aging Research German Cancer Research Center Im Neuenheimer Feld 581 69120 Heidelberg Germany; ^2^ Faculty of Medicine University of Heidelberg Im Neuenheimer Feld 672 69120 Heidelberg Germany; ^3^ Saarland Cancer Registry Neugeländstraße 9 66117 Saarbrücken Germany; ^4^ Division of Preventive Oncology German Cancer Research Center Im Neuenheimer Feld 460 69120 Heidelberg Germany

**Keywords:** all‐cause mortality, cohort studies, metabolomic age acceleration, metabolomics, risk prediction

## Abstract

Metabolite concentrations vary by age and sex, yet age‐ and sex‐specific metabolomic risk scores and biological ageing clocks for mortality prediction remain undeveloped. Nuclear magnetic resonance (NMR)‐based metabolomic profiling is conducted in 209144 UK Biobank participants (12347 deaths) and 6820 from the German ESTHER study (804 deaths). Mortality risk scores are derived using least absolute shrinkage and selection operator (LASSO)‐regularized Cox regression, and metabolomics‐based mortality risk clocks (MetaboMR clocks) are constructed using elastic net regression in sex‐ and age‐stratified subgroups (50–59 and 60–69 years). Models are trained in 70% of UK Biobank and validated internally (30%) and externally in ESTHER. 68 metabolites are significantly associated with 10‐year all‐cause mortality in both cohorts. 20, 18, 12, and 13 metabolites improved 10‐year mortality prediction in younger and older men, younger and older women. Metabolite‐augmented models improved c‐statistics by 0.036–0.084 across subgroups. In the external validation set, each year of age acceleration is associated with an 8% and 9% higher 10‐year mortality risk for MetaboMR clock1 and clock2. Sex‐ and age‐specific metabolomic risk scores significantly enhance 10‐year mortality prediction beyond traditional models. The MetaboMR clocks may serve as measures of biological ageing and support personalized risk stratification in clinical settings.

## Graphical abstract

Metabolomics‐based ageing clocks developed using blood biomarkers from over 215 000 European adults, improved the prediction of 10‐year all‐cause mortality compared to traditional models. Biological age acceleration is associated with higher mortality risk, as each additional year of metabolic age corresponded to at least 8% increased risk. These tools may support personalized risk assessment and provide insights into the biological ageing process.

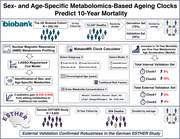

## 1 Introduction

Numerous prognostic models have been developed based on routinely collected demographic, lifestyle, and clinical information to predict short‐ and long‐term mortality in middle‐aged adults, older adults, and high‐risk populations.^[^ ^1^, ^2^, ^3^ ^]^ While these models enable useful risk stratification, they rely primarily on non‐biological variables. Although metabolic biomarkers are biologically informative and increasingly accessible,^[^ ^4^ ^]^ they have yet to be systematically integrated into stratified mortality prediction.

Metabolomics also holds promise for elucidating biological mechanisms underlying premature mortality, offering insights beyond traditional risk factors. A growing body of evidence has linked individual metabolites, such as glycoprotein acetyls (GlycA), albumin, citrate, and various lipoproteins, that are independently associated with mortality.^[^ ^4^, ^5^, ^6^, ^7^, ^8^, ^9^, ^10^, ^11^, ^12^ ^]^ Many of these metabolites can be reliably quantified using nuclear magnetic resonance (NMR)‐based platforms, which enable high‐throughput and reproducible measurement in large‐scale epidemiologic studies.

Prior studies, particularly those based on the UK Biobank, have examined associations between metabolomic profiles and mortality in both the general population^[^ ^11^, ^12^, ^13^, ^14^, ^15^, ^16^ ^]^ and specific subgroups.^[^ ^17^, ^18^, ^19^, ^20^ ^]^ Recent advances include the development of metabolomic ageing clocks, which estimate biological age and predict mortality risk.^[^ ^11^, ^13^, ^14^, ^15^ ^]^ These studies have improved our understanding of how metabolic dysregulation contributes to ageing and disease progression.^[^ ^21^ ^]^ However, these previous studies did not develop age‐ and sex‐specific metabolomic clocks, nor did they validate such models in independent external cohorts.

Moreover, metabolomic risk scores have demonstrated improved predictive performance when added to conventional clinical models.^[^ ^4^, ^5^, ^6^, ^7^, ^8^, ^9^, ^12^, ^15^ ^]^ Although some of these studies applied sex‐stratified biomarker selection^[^ ^7^ ^]^ or conducted post hoc sex‐ and age‐stratified analyses,^[^ ^4^, ^6^, ^8^ ^]^ none of them accounted for age‐specific metabolic variations during model development, despite evidence that metabolic pathways differ significantly across age groups.^[^ ^22^, ^23^, ^24^, ^25^ ^]^ While some studies included external validation, they were limited by either a narrow metabolite panel (e.g., only 98 metabolic variables in UK and Finnish cohorts^[^ ^13^ ^]^) or small sample sizes (e.g., a 250‐metabolite‐derived MASH risk score developed in small Chinese and European liver disease cohorts^[^ ^18^ ^]^).

To address these gaps, we aimed to (I) identify metabolomic biomarkers associated with all‐cause mortality, (II) map them on metabolic pathways linked to mortality, (III) construct and externally validate sex‐ and age‐specific mortality prediction models integrating identified metabolomic biomarkers and conventional risk factors, and (IV) develop and evaluate novel metabolomics‐based mortality clocks (MetaboMR clocks) as measures of biological ageing.

## 2 Results

### 2.1 Distribution of Traditional Risk Factors and Their Associations with 10‐Year Mortality in the UK Biobank and ESTHER Study

The baseline characteristics of the 6820 participants in the ESTHER study were broadly comparable to the 209144 participants in the UK Biobank (**Table** **1**), with similar mean age (60.4 ± 5.5 years vs 60.0 ± 5.4 years) and proportion of women (55.0% vs 53.9%). The distribution of education levels, physical activity levels, body mass index (BMI), and the prevalence of hypertension, diabetes, cardiovascular disease, cancer history, and treated dyslipidemia were also consistent across cohorts. Differences were observed in smoking and alcohol consumption, with the proportion of current smokers being higher in the ESTHER study than in the UK Biobank (17.6% vs 9.5%) and the proportion of participants with moderate or high alcohol consumption being higher in the UK Biobank than in the ESTHER study (29.4% vs 8.1%).

**Table 1:** Baseline characteristics of study participants in the UK Biobank and ESTHER study.

Characteristics	UK Biobank			ESTHER Study
	Derivation Set (*n* = 146400)	Internal Test Set (*n* = 62744)	Overall (*n* = 209144)	External Validation Set (*n* = 6820)
Age, years	60.0 (5.4)	60.0 (5.4)	60.0 (5.4)	60.4 (5.5)
Women, n (%)	78856 (53.9)	33976 (54.2)	112832 (53.9)	3753 (55.0)
High education level, n (%) ^a)^, ^g)^	41319 (28.2)	17655 (28.1)	58974 (28.2)	1744 (25.6)
Smoking status, n (%) ^g)^	/	/	/	/
Never smoker	77236 (52.8)	33109 (52.8)	110345 (52.8)	3420 (50.1)
Former smoker	55227 (37.7)	23686 (37.7)	78913 (37.7)	2198 (32.3)
Current smoker	13937 (9.5)	5949 (9.5)	19886 (9.5)	1202 (17.6)
Physical activity, n (%) ^g)^	/	/	/	/
Low	30595 (20.9)	13277 (21.2)	43872 (21.0)	1347 (19.8)
Moderate	60424 (41.3)	25693 (40.9)	86117 (41.2)	3089 (45.2)
High	55381 (37.8)	23774 (37.9)	79155 (37.8)	2384 (35.0)
Alcohol consumption, n (%) ^b)^, ^g)^	/	/	/	/
Abstainer	44669 (30.5)	19280 (30.7)	63949 (30.6)	2242 (32.9)
Low	58589 (40.0)	25074 (40.0)	83663 (40.0)	4028 (59.0)
Medium or high	43142 (29.5)	18390 (29.3)	61532 (29.4)	550 (8.1)
Body mass index, n (%) ^c)^	/	/	/	/
< 21.5 kg m^−2^	9560 (6.4)	4062 (6.5)	13622 (6.5)	352 (5.2)
≥ 21.5 – < 25 kg m^−2^	36513 (25.0)	15736 (25.1)	52249 (25.0)	1539 (22.6)
≥ 25 – < 30 kg m^−2^	63552 (43.4)	27126 (43.2)	90678 (43.3)	3189 (46.7)
≥ 30 – < 35 kg m^−2^	26709 (18.2)	11345 (18.1)	38054 (18.2)	1346 (19.7)
≥ 35 kg m^−2^	10066 (7.0)	4475 (7.1)	14541 (7.0)	394 (5.8)
Medical conditions, n (%)	/	/	/	/
Treated dyslipidemia ^g)^	31879 (21.8)	13566 (21.6)	45445 (21.7)	712 (10.4)
Hypertension ^d)^, ^g)^	85968 (58.7)	36819 (58.7)	122787 (58.7)	4146 (60.8)
Diabetes ^e)^	15075 (10.3)	6503 (10.4)	21578 (10.3)	964 (14.1)
Cardiovascular disease ^f)^, ^g)^	10791 (7.4)	4567 (7.3)	15358 (7.3)	503 (7.4)
History of cancer ^g)^	12825 (8.8)	5541 (8.8)	18366 (8.8)	471 (6.9)

Notes: Values are means (standard deviations, SDs) for continuous variables and percentages for categorical variables.

^a)^ In the UK Biobank, participants who reported “None of the above”, “Certificate of Secondary Education (CSEs) or equivalent”, “Ordinary Levels/General Certificate of Secondary Education (O levels/GCSEs) or equivalent”, “Other professional qualifications (e.g., nursing, teaching)”, or “Advanced Levels/Advanced Subsidiary Levels (A levels/AS levels) or equivalent” were categorized as having a low or medium education level. Those who reported “National Vocational Qualification (NVQ), Higher National Diploma (HND), Higher National Certificate (HNC) or equivalent”, or a “College or University degree” were categorized as having a higher education level. In the German ESTHER study, participants who reported “No education or “Hauptschule”” or ““Realschule”/“Mittlere Reife”” were categorized as having a low education level, whereas those who reported ““Fachhochschulreife” or “Abitur”” were categorized as having a high education level;

^b)^ Alcohol consumption was categorized as low for women consuming 0–19.99 g day^−1^ and men consuming 0–39.99 g day^−1^. It was categorized as medium or high for women consuming 20–39.99 g day^−1^ or ≥40 g day^−1^, and for men consuming 40–59.99 g day^−1^ or ≥60 g day^−1^;

^c)^ To enhance comparability between the two cohorts, body mass index (BMI) categories were defined according to the WHO classification with slight modifications to the cut‐off values. Participants with a BMI less than 21.5 kg/m^2^, including both underweight individuals and those at the lower end of the normal range, were grouped together to account for population‐specific differences and to ensure sufficient sample sizes across categories;

^d)^ Hypertension was defined as self‐reported hypertension, the use of antihypertensive medication, a systolic blood pressure ≥140 mm Hg, and a diastolic blood pressure ≥90 mm Hg;

^e)^ In this study, diabetes was defined as self‐reported diabetes, the use of glucose‐lowering medication, or an HbA_1c_ level of ≥6.5%;

^f)^ Cardiovascular disease in this study was defined as having myocardial infarction or stroke;

^g)^ Missing values for education level (1.2%), smoking status (0.1%), physical activity (9.6%), hypertension (0.02%), diabetes (0.02%), lipid‐lowering medication (0.02%), and cancer (0.4%) in the UK Biobank, as well as, education level (2.0%), smoking status (2.4%), physical activity (0.2%), alcohol consumption (8.0%), diabetes (1.7%), and cardiovascular disease (2.6%) in the ESTHER study, were imputed using random forest imputation with the R package *missRanger* (version 2.1.5).

During a median follow‐up of 10 years (mean duration: 9.75 years in the UK Biobank and 9.56 years in the ESTHER study), a total of 12347 deaths occurred in the UK Biobank and 804 deaths in the ESTHER study (Figure S1, Supporting Information).

In both the UK Biobank and ESTHER study, older age, male sex, current smoking, low physical activity, low weight (BMI<21.5 kg m^−^^2^), high weight (BMI≥35 kg m^−^^2^), hypertension, diabetes, cardiovascular disease, and cancer history were associated with an increased risk of 10‐year all‐cause mortality (Table S1, Supporting Information). The HRs for these risk and preventive factors were highly comparable between the two cohorts. Treated dyslipidemia was not significantly associated with mortality in either cohort. Notable differences were observed for medium or high alcohol consumption (associated with increased mortality in the UK Biobank only) and higher education (associated with lower mortality in the UK Biobank only).

### 2.2 Associations of Metabolomic Biomarkers with All‐Cause Mortality

**Figure 1:**
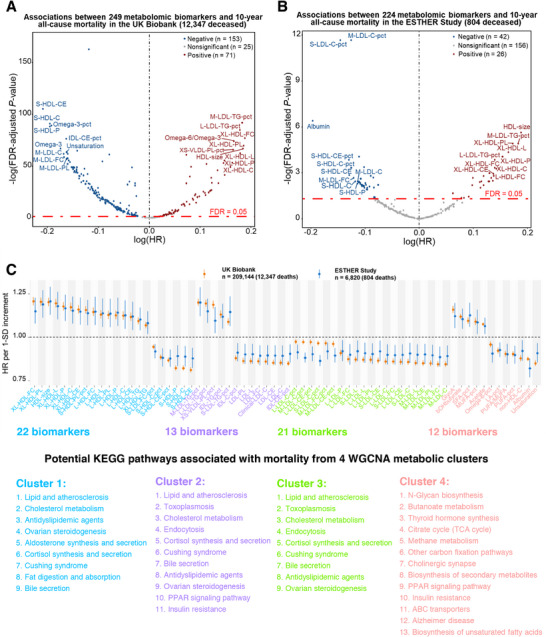
Metabolome‐wide discovery and validation of biomarkers associated with 10‐year all‐cause mortality in the UK Biobank and ESTHER study. A) In the UK Biobank (*n* = 209144; 12347 deaths), 224 of 249 measured metabolomic biomarkers showed significant associations with 10‐year all‐cause mortality in multivariable Cox regression analyses. The 10 biomarkers with the strongest positive and inverse HRs are highlighted by their abbreviations (the full names are detailed in Table S10 (Supporting Information)). B) Of these, 68 biomarkers were validated in the ESTHER study (*n* = 6820; 804 deaths). The corresponding volcano plot depicts their effect estimates. C) Forest plots display HRs with 95% CIs per 1‐SD increment for the validated metabolomic biomarkers in both cohorts. The 68 biomarkers were grouped into four co‐expression clusters by WGCNA; KEGG pathway enrichment analyses for each cluster suggested biological processes of potential relevance, summarized below the plot. All models were adjusted for age, sex, education level, smoking status, physical activity, alcohol consumption, body mass index, treated dyslipidemia, hypertension, diabetes, cardiovascular disease, and cancer. Abbreviations: CI, confidence interval; FDR, false discovery rate; HR, hazard ratio; KEGG, Kyoto Encyclopedia of Genes and Genomes; WGCNA, Weighted Gene Co‐expression Network Analysis.

Among 209144 UK Biobank participants, 224 of the 249 metabolomic biomarkers were significantly associated with 10‐year all‐cause mortality after adjustment for conventional risk factors and multiple testing (false discovery rate [FDR]‐adjusted *P *< 0.05) (**Figure** **1**A; Table S2, Supporting Information). Of these, 68 biomarkers were validated in the independent ESTHER cohort (*n* = 6820), including 26 with positive and 42 with negative associations with mortality (FDR‐adjusted *P *< 0.05) (Figure 1B; Table S3, Supporting Information). Most of the validated biomarkers were related to lipid metabolism.

### 2.3 Metabolic Pathways Associated with All‐Cause Mortality

To take into account inter‐correlations of the 68 validated metabolomic biomarkers, we conducted weighted gene co‐expression network analysis (WGCNA) in the UK Biobank and identified 4 clusters (Figure S2, Supporting Information). Table S4 (Supporting Information) lists the Kyoto Encyclopedia of Genes and Genomes (KEGG)‐based pathways enriched in these clusters. Figure 1C gives an overview on the strengths of the associations of the 68 validated metabolomic biomarkers, their clustering, and potential KEGG pathways. Cluster 1 (22 biomarkers) primarily consists of high‐density lipoprotein (HDL) particles and is enriched in pathways related to lipid metabolism, cardiovascular function, and endocrine regulation. Clusters 2 and 3 (13 and 21 biomarkers, respectively) are dominated by intermediate‐density lipoprotein (IDL), low‐density lipoprotein (LDL), and very‐low‐density lipoprotein (VLDL) particles and are associated with pathways involved in cardiovascular disease, lipid metabolism, and metabolic disorders. Cluster 4 (12 biomarkers) includes GlycA, *β*‐hydroxybutyrate (bOHbutyrate), various fatty acid markers, acetate, non‐HDL cholesterol, and albumin. This cluster is enriched in pathways related to carbohydrate metabolism, energy metabolism, endocrine signaling, and neurodegenerative diseases, including Alzheimer's disease.

### 2.4 Selected Metabolomic Biomarkers for Predicting 10‐Year All‐Cause Mortality

**Figure 2:**
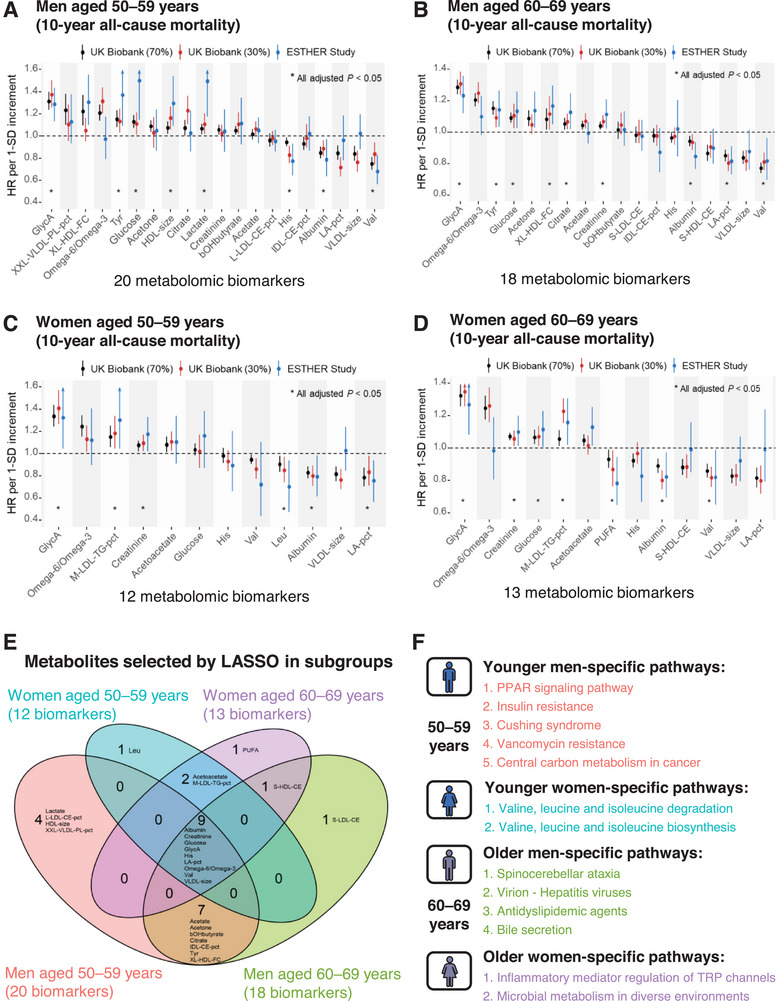
Sex‐ and age‐specific metabolomic biomarkers associated with 10‐year all‐cause mortality in A) younger men, B) older men, C) younger women, and D) older women; E) their overlap in age‐, and sex‐specific subgroups and F) their subgroup‐specific enriched KEGG pathways. Associations between LASSO‐selected metabolomic biomarkers and 10‐year all‐cause mortality (panel A–D) were assessed in multivariable Cox models adjusted for age, sex, education level, smoking status, physical activity, alcohol consumption, body mass index, treated dyslipidemia, hypertension, diabetes, cardiovascular disease, and cancer. HRs and 95% CIs per 1‐SD increment are shown across the derivation set (black; 70% of the UK Biobank), the internal validation set (red; remaining 30% of the UK Biobank), and the external validation set (blue; ESTHER study). Asterisks (*) indicate biomarkers that were consistently and significantly associated with mortality across all three sets. Abbreviations: HR, hazard ratio; CI, confidence interval; SD, standard deviation; LASSO, least absolute shrinkage and selection operator; KEGG, Kyoto Encyclopedia of Genes and Genomes.

To develop all‐cause mortality prediction models, we applied least absolute shrinkage and selection operator (LASSO) regularized Cox regression to identify sex‐ and age‐specific metabolomic biomarkers from a panel of 249 metabolites (**Figure** **2**). A total of 20 biomarkers in younger men (Figure 2A), 18 in older men (Figure 2B), 12 in younger women (Figure 2C), and 13 in older women (Figure 2D) were selected for risk score development. In models adjusted for conventional risk factors, 8, 9, 6, and 7 biomarkers, respectively, were statistically significantly associated with 10‐year all‐cause mortality across the derivation (UK Biobank 70%), internal validation (UK Biobank 30%), and external validation (ESTHER study) sets with consistent HRs per 1‐SD increment. Among these validated biomarkers, the inflammatory biomarkers, GlycA and albumin, were statistically significantly associated with 10‐year mortality in all age‐ and sex‐specific strata. Figure S3 (Supporting Information) ranks the standardized *β*‐coefficients of the selected metabolomic biomarkers in the sex‐ and age‐specific risk scores. GlycA and the Omega‐6/Omega‐3 ratio carried strong positive weights (*β*>0.15) in all 4 subgroups, whereas VLDL size (in all 4 subgroups), LA‐pct (in 3 subgroups), valine, and albumin (each in 2 subgroups) showed large negative weights (*β*<‐0.15) in more than one subgroup. These leading metabolites largely reflect pathways of systemic inflammation, energy metabolism, and lipid regulation.

Overall, 26 metabolomic biomarkers were selected and Figure 2E illustrates the overlap in the selection of metabolites in the subgroups. Nine biomarkers were selected in all four subgroups, 12 biomarkers were uniquely detected in men, four biomarkers were identified exclusively in women, and one biomarker was selected specifically in the older men and older women subgroups. The subgroup‐specific pathways highlighted distinct biological processes: in younger men, pathways related to insulin resistance, glucocorticoid activity, and cancer metabolism predominated; in older men, bile acid metabolism and viral infection pathways were enriched; in younger women, branched‐chain amino acid metabolism was prominent; and in older women, pathways related to inflammation and host‐microbial interactions were identified (Figure 2F).

### 2.5 Discrimination and Reclassification Analyses of 10‐Year Mortality Prediction Models Including Metabolomic Biomarkers

Since the 26 selected metabolomic biomarkers were neither strongly correlated with the conventional risk factors (absolute Spearman correlation coefficients |*ρ*|≤0.45) nor with each other (|*ρ*|≤0.67) (Figure S4, Supporting Information), we evaluated whether combined models of traditional risk factors and metabolomic biomarkers could enhance risk discrimination for 10‐year all‐cause mortality beyond conventional risk factors. Risk scores were derived in sex‐ and age‐specific groups using Cox regression and validated in both internal and external validation cohorts. The variables’ *β*‐coefficients, obtained in the derivation set and used in the two validation sets, are provided in Table S5 (Supporting Information).

Across all analyses, models based solely on metabolomic biomarkers yielded lower c‐statistics compared to traditional risk factor models (**Table** **2**). However, the c‐statistics of the metabolomics models were significantly higher than 0.5 in the range of 0.639–0.684 across the subgroups for 10‐year all‐cause mortality prediction. The c‐statistics for 5‐year all‐cause mortality prediction in the total study population were slightly higher than for 10‐year all‐cause mortality prediction in the derivation, internal validation, and external validation set (0.701, 0.685, and 0.673 vs 0.690, 0.679, and 0.662, respectively).

**Table 2:** C‐statistics of prediction models for 10‐ and 5‐year all‐cause mortality in the UK Biobank and ESTHER cohorts.

	C‐statistic (*P*) ^g)^		
	UK Biobank (derivation set, 70%)	UK Biobank (internal validation set, 30%)	ESTHER Study (external validation)
**10‐year all‐cause mortality in men aged 50–59 years**	***n* = 28158** **(1233 deaths)**	***n* = 12043** **(522 deaths)**	***n* = 1557** **(120 deaths)**
Traditional risk factor model ^a)^	0.781 (0.766, 0.795)	0.776 (0.754, 0.799)	0.732 (0.688, 0.776)
Metabolomics model ^b)^	0.684 (0.669, 0.698)	0.672 (0.649, 0.696)	0.658 (0.612, 0.706)
Combined model	0.844 (0.830, 0.858)	0.831 (0.811, 0.852)	0.816 (0.779, 0.852)
	*ΔC* = 0.063; *P *< 0.001	*ΔC* = 0.055; *P *< 0.001	*ΔC* = 0.084; *P *< 0.001
**10‐year all‐cause mortality in men aged 60–69 years**	***n* = 39386** **(4041 deaths)**	***n* = 16725** **(1699 deaths)**	***n* = 2196** **(349 deaths)**
Traditional risk factor model ^a)^	0.742 (0.734, 0.750)	0.733 (0.724, 0.741)	0.724 (0.695, 0.753)
Metabolomics model ^c)^	0.681 (0.672, 0.691)	0.667 (0.656, 0.677)	0.650 (0.621, 0.679)
Combined model	0.795 (0.787, 0.803)	0.781 (0.771, 0.790)	0.779 (0.751, 0.808)
	*ΔC* = 0.053; *P *< 0.001	*ΔC* = 0.048; *P *< 0.001	*ΔC* = 0.055; *P *< 0.001
**10‐year all‐cause mortality in women aged 50–59 years**	***n* = 35553** **(890 deaths)**	***n* = 15254** **(411 deaths)**	***n* = 1201** **(95 deaths)**
Traditional risk factor model ^a)^	0.746 (0.728, 0.764)	0.737 (0.709, 0.765)	0.725 (0.676, 0.774)
Metabolomics model ^d)^	0.678 (0.660, 0.697)	0.665 (0.637, 0.693)	0.647 (0.594, 0.700)
Combined model	0.800 (0.783, 0.817)	0.787 (0.762, 0.811)	0.761 (0.712, 0.810)
	*ΔC* = 0.054; *P *< 0.001	*ΔC* = 0.050; *P *< 0.001	*ΔC* = 0.036; *P =*0.065
**10‐year all‐cause mortality in women aged 60–69 years**	***n* = 43303** **(2467 deaths)**	***n* = 18722** **(1084 deaths)**	***n* = 1866** **(240 deaths)**
Traditional risk factor model ^a)^	0.750 (0.739, 0.761)	0.735 (0.718, 0.753)	0.714 (0.679, 0.749)
Metabolomics model ^e)^	0.670 (0.658, 0.681)	0.654 (0.637, 0.672)	0.639 (0.605, 0.674)
Combined model	0.797 (0.786, 0.809)	0.777 (0.762, 0.793)	0.769 (0.738, 0.799)
	*ΔC* = 0.047; *P *< 0.001	*ΔC* = 0.042; *P *< 0.001	*ΔC* = 0.055; *P *< 0.001
**10‐year all‐cause mortality in the total study population**	***n* = 146400** **(8631 deaths)**	***n* = 62744** **(3716 deaths)**	***n* = 6820** **(804 deaths)**
Traditional risk factor model ^a)^	0.757 (0.751, 0.763)	0.723 (0.715, 0.732)	0.720 (0.700, 0.741)
Metabolomics model ^f)^	0.690 (0.684, 0.696)	0.679 (0.670, 0.688)	0.662 (0.645, 0.679)
Combined model	0.833 (0.827, 0.839)	0.796 (0.788, 0.804)	0.788 (0.770, 0.808)
	*ΔC* = 0.076; *P *< 0.001	*ΔC* = 0.073; *P *< 0.001	*ΔC* = 0.068; *P *< 0.001
**5‐year all‐cause mortality in the total study population**	***n* = 146400** **(2944 deaths)**	***n* = 62744** **(1317 deaths)**	***n* = 6820** **(312 deaths)**
Traditional risk factor model ^a)^	0.761 (0.749, 0.773)	0.731 (0.717, 0.745)	0.734 (0.705, 0.762)
Metabolomics model ^f)^	0.701 (0.690, 0.712)	0.685 (0.671, 0.699)	0.673 (0.646, 0.700)
Combined model	0.856 (0.847, 0.865)	0.818 (0.805, 0.831)	0.812 (0.788, 0.836)
	*ΔC* = 0.095; *P *< 0.001	*ΔC* = 0.087; *P *< 0.001	*ΔC* = 0.078; *P *< 0.001

^a)^ Variables of the traditional risk factor models are: age, sex, education level, smoking status, physical activity, alcohol consumption, body mass index, treated dyslipidemia, hypertension, diabetes, cardiovascular disease, and cancer;

^b)^ Metabolites included in this model were (see Table S10 (Supporting Information) for abbreviations): GlycA, XXL‐VLDL‐PL‐pct, XL‐HDL‐FC, Omega‐6/Omega‐3, Tyr, Glucose, Acetone, HDL‐size, Citrate, Lactate, Creatinine, bOHbutyrate, Acetate, L‐LDL‐CE‐pct, His, IDL‐CE‐pct, Albumin, LA‐pct, VLDL‐size, Val;

^c)^ Metabolites included in this model were (see Table S10 (Supporting Information) for abbreviations): GlycA, Omega‐6/Omega‐3, Tyr, Glucose, Acetone, XL‐HDL‐FC, Citrate, Acetate, Creatinine, bOHbutyrate, S‐LDL‐CE, IDL‐CE‐pct, His, Albumin, S‐HDL‐CE, LA‐pct, VLDL‐size, Val;

^d)^ Metabolites included in this model were (see Table S10 (Supporting Information) for abbreviations): GlycA, Omega‐6/Omega‐3, M‐LDL‐TG‐pct, Creatinine, Acetoacetate, Glucose, His, Val, Leu, Albumin, VLDL‐size, LA‐pct;

^e)^ Metabolites included in this model were (see Table S10 (Supporting Information) for abbreviations): GlycA, Omega‐6/Omega‐3, Creatinine, Glucose, M‐LDL‐TG‐pct, Acetoacetate, PUFA, His, Albumin, S‐HDL‐CE, Val, VLDL‐size, LA‐pct;

^f)^ Metabolites included in this model were (see Table S10 (Supporting Information) for abbreviations): GlycA, XXL‐VLDL‐PL‐pct, XL‐HDL‐FC, Omega‐6/Omega‐3, Tyr, Glucose, Acetone, HDL‐size, Citrate, Lactate, Creatinine, bOHbutyrate, Acetate, L‐LDL‐CE‐pct, S‐LDL‐CE, IDL‐CE‐pct, M‐LDL‐TG‐pct, Acetoacetate, PUFA, His, Val, Leu, Albumin, S‐HDL‐CE, LA‐pct, VLDL‐size;

^g)^ The c‐statistic of the combined model was tested against the c‐statistic of the traditional risk factor model with the DeLong test.

Combining metabolomic biomarkers with traditional risk factors significantly improved risk discrimination, as reflected by increases in the c‐statistic compared to models with conventional risk factors alone (Table 2; Figure S5, Supporting Information). Improvements in 10‐year mortality prediction were observed in all the derivation (70% UK Biobank), internal validation (30% UK Biobank), and external validation (ESTHER) in younger men (+0.063, +0.055, and +0.084, respectively), older men (+0.053, +0.048, and +0.055, respectively), younger women (+0.054, +0.050, and +0.036, respectively), and older women (+0.047, +0.042, and +0.055, respectively). In the total study population, the c‐statistic increased from 0.757 to 0.833 (+0.076), from 0.723 to 0.796 (+0.073), and from 0.720 to 0.788 (+0.068) in the derivation, internal validation, and external validation set, respectively. The improvement in the total population was even more pronounced for 5‐year mortality risk prediction (c‐statistic increase: +0.095, +0.087, +0.078, respectively). When participants receiving lipidlowering therapy at baseline were excluded, the improvements in risk discrimination remained robust (Table S6, Supporting Information).

Moreover, reclassification analyses demonstrated statistically significant improvements in net reclassification improvement (NRI) and integrated discrimination improvement (IDI) for 10‐year all‐cause mortality across all sex‐ and age‐specific subgroups in both the internal and external validation cohorts, except for the group of younger women in the ESTHER study (Table S7, Supporting Information). With the exception of this group, the categorical NRIs, continuous NRIs, and IDIs ranged from 8.2%–18.9%, to 24.4%–41.3%, and 4.6%–12.4%, respectively across the strata and validation sets for 10‐year mortality prediction. The categorical NRIs, continuous NRIs, and IDIs ranges were even higher for 5‐year mortality prediction in the total study population: 19.1%–24.7%, 33.4%–42.2%, and 9.8%–12.3% respectively.

Calibration curves showed that models based on conventional risk factors were well‐calibrated in both studies and all subgroups except for younger women in ESTHER (Figure S6, Supporting Information). Adding metabolomic biomarkers further improved model calibration by bringing predicted and observed mortality rates closer to the ideal calibration line, which represents perfect agreement between predicted and observed cases. The model also got well‐calibrated in the group of younger women from the ESTHER study after inclusion of the metabolomic biomarkers.

### 2.6 Development and Testing of Metabolomic Ageing Clocks

**Figure 3:**
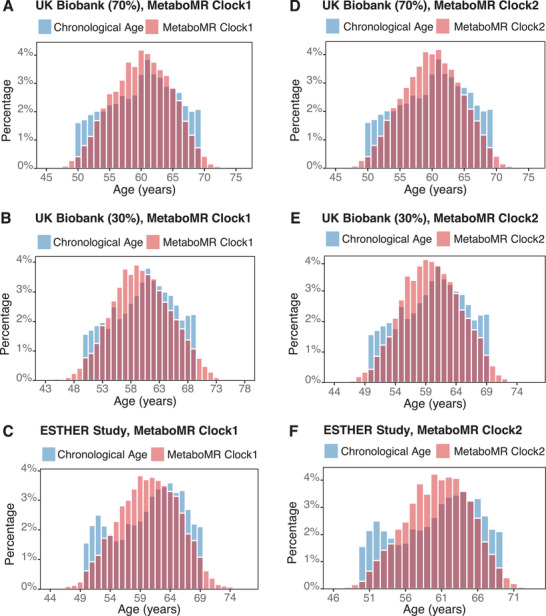
Distributions of chronological age and metabolomics‐based mortality‐related ageing clocks in the UK Biobank and ESTHER cohorts. A–C) MetaboMR clock1 (based on LASSO‐selected metabolomic biomarkers only); D–F) MetaboMR clock2 (based on LASSO‐selected metabolomic biomarkers + conventional risk factors). Abbreviations: LASSO, least absolute shrinkage and selection operator; MetaboMR, metabolomics‐based mortality risk.

**Figure 4:**
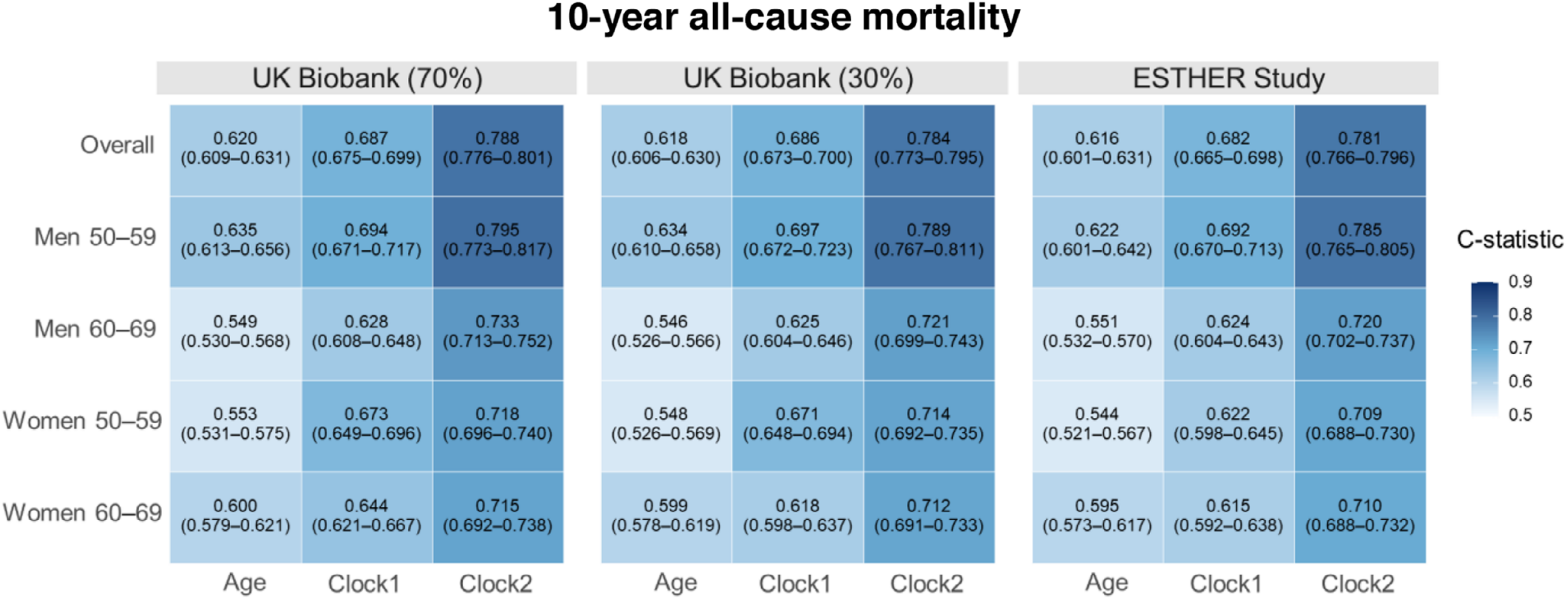
Comparison of the C‐statistics of chronological age and the MetaboMR clocks for 10‐year mortality prediction in the UK Biobank and ESTHER Study. The figure shows c‐statistics with 95% confidence intervals.

The equations used to calculate the MetaboMR clock1 and MetaboMR clock2 are provided in Table S8 (Supporting Information). The distributions of chronological age and the biological age according to the two clocks in the training, internal validation, and external validation datasets are illustrated in **Figure** **3**A–F. Figure S7 (Supporting Information) shows strong correlations between the MetaboMR clock1, which was derived from the selected metabolomic biomarkers only, and chronological age across all subgroups and in the overall study population (training set: *r* = 0.83–0.85; internal validation set: *r* = 0.82–0.85; external validation set: *r* = 0.80–0.83). The corresponding Pearson correlation coefficients for the MetabMR clock2, which was derived from the biomarkers and traditional mortality risk factors, were slightly higher (Figure S8, Supporting Information; training set: *r* = 0.85–0.86; internal validation set: *r *= 0.84–0.86; external validation set: *r* = 0.80–0.84). The c‐statistics for 10‐year mortality prediction using the MetaboMR clocks were substantially higher than the c‐statistics of chronological age in the total population of both studies and in all age and sex‐specific subgroups, and MetaboMR clock2 outperformed MetaboMR clock1 (**Figure** **4**). For instance, in the external validation cohort (ESTHER), the MetaboMR clock2 achieved a c‐statistic of 0.781 (0.766–0.796) in the total population, followed by a c‐statistic of 0.682 (0.665–0.698) of MetaboMR clock1, whereas the c‐statistic of the chronological age was 0.616 (0.601–0.631).

The distributions of metabolomic age acceleration derived from these clocks are shown in Figure S9 (Supporting Information). The metabolomic age acceleration derived from the MetaboMR clock1 (MetAA1) and MetaboMR clock2 (MetAA2) were consistently, significantly associated with 10‐year all‐cause mortality across all sub‐groups and the total population in both the internal and external validation sets with stronger associations among men than women (**Table** **3**). Overall, the results for MetAA1 and MetAA2 were comparable. In the total internal validation set (UKB) and total external validation set (ESTHER), each year increase of MetAA1 was significantly associated with a 10‐year all‐cause mortality increase in the adjusted model of 9% (HR [95%CI], 1.09 [1.08–1.10]) and 8% (HR [95%CI], 1.08 [1.06–1.09]), respectively. Excluding subjects on lipid‐lowering therapy led to almost the same results (Table S9, Supporting Information).

**Table 3:** Association of metabolomic age acceleration based on metabolomic biomarkers only (MetAA1) and based on both biomarkers and traditional risk factors (MetAA2) with 10‐year all‐cause mortality.

MetAA / Study population	UK Biobank (derivation set, 70%)			UK Biobank (internal validation set, 30%)			ESTHER Study (external validation)		
	Mean MetAA ± SD (years)	Crude HR per 1‐year MetAA increase ^a)^	Adjusted HR per 1‐year MetAA increase ^b)^	Mean MetAA ± SD (years)	Crude HR per 1‐year MetAA increase ^a)^	Adjusted HR per 1‐year MetAA increase ^b)^	Mean MetAA ± SD (years)	Crude HR per 1‐year MetAA increase ^a)^	Adjusted HR per 1‐year MetAA increase ^b)^
**MetAA1**									
**Men aged 50–59 years**	0.00 ± 5.71	**1.16 (1.12–1.20)**	**1.13 (1.09–1.17)**	0.27 ± 5.94	**1.20 (1.14–1.26)**	**1.17 (1.11–1.23)**	0.40 ± 5.76	**1.19 (1.08–1.32)**	**1.16 (1.04–1.29)**
**Men aged 60–69 years**	0.00 ± 5.82	**1.20 (1.18–1.22)**	**1.17 (1.15–1.19)**	0.30 ± 6.09	**1.19 (1.17–1.23)**	**1.17 (1.14–1.20)**	0.51 ± 5.95	**1.20 (1.14–1.26)**	**1.19 (1.13–1.26)**
**Women aged 50–59 years**	0.00 ± 5.73	**1.13 (1.09–1.17)**	**1.10 (1.05–1.14)**	0.17 ± 5.66	**1.12 (1.06–1.19)**	**1.10 (1.04–1.16)**	0.47 ± 6.14	**1.11 (1.01–1.23)**	**1.11 (1.00–1.23)**
**Women aged 60–69 years**	0.00 ± 5.77	**1.18 (1.16–1.21)**	**1.16 (1.14–1.19)**	0.22 ± 5.88	**1.19 (1.16–1.23)**	**1.17 (1.13–1.21)**	0.53 ± 5.77	**1.13 (1.05–1.20)**	**1.10 (1.03–1.18)**
**Total study population**	0.00 ± 5.81	**1.12 (1.11–1.13)**	**1.10 (1.09–1.11)**	0.24 ± 5.62	**1.11 (1.10–1.12)**	**1.09 (1.08–1.10)**	0.48 ± 6.08	**1.10 (1.09–1.12)**	**1.08 (1.06–1.09)**
**MetAA2**									
**Men aged 50–59 years**	0.00 ± 5.42	**1.14 (1.10–1.18)**	**N.A**.^c)^	0.16 ± 5.73	**1.17 (1.11–1.23)**	**N.A**.^c)^	0.34 ± 5.62	**1.15 (1.04–1.28)**	**N.A**.^c)^
**Men aged 60–69 years**	0.00 ± 5.58	**1.19 (1.17–1.21)**	**N.A**.^c)^	0.23 ± 5.84	**1.18 (1.16–1.22)**	**N.A**.^c)^	0.46 ± 5.81	**1.17 (1.11–1.23)**	**N.A**.^c)^
**Women aged 50–59 years**	0.00 ± 5.65	**1.12 (1.08–1.16)**	**N.A**.^c)^	0.12 ± 5.49	**1.11 (1.05–1.18)**	**N.A**.^c)^	0.39 ± 6.13	**1.10 (1.01–1.22)**	**N.A**.^c)^
**Women aged 60–69 years**	0.00 ± 5.70	**1.15 (1.13–1.18)**	**N.A**.^c)^	0.20 ± 5.76	**1.16 (1.13–1.20)**	**N.A**.^c)^	0.42 ± 5.97	**1.12 (1.04–1.19)**	**N.A**.^c)^
**Total study population**	0.00 ± 5.57	**1.11 (1.10–1.12)**	**N.A**.^c)^	0.18 ± 5.61	**1.10 (1.09–1.11)**	**N.A**.^c)^	0.37 ± 5.79	**1.09 (1.08–1.11)**	**N.A**.^c)^

Abbreviations: CI, confidence interval; HR, hazard ratio; MetAA, metabolomic age acceleration; N.A., not applicable; SD, standard deviation.

Notes: Values printed in bold indicate statistical significance (*P *< 0.05).

^a)^ HRs for mortality were estimated per 1‐year increase in metabolomic age acceleration (MetAA1), reflecting the relative increase in mortality risk for each year that a person's metabolomic age exceeds their chronological age;

^b)^ The adjusted Cox proportional hazards regression model included the following covariates: chronological age, sex, education level, smoking status, physical activity, alcohol consumption, body mass index, treated dyslipidemia, hypertension, diabetes, cardiovascular disease, and cancer;

^c)^ As the MetaboMR clock2 already includes the traditional risk factors, it cannot be further adjusted for them.

MetAAs were slightly stronger associated with cardiovascular disease (CVD) mortality than with cancer mortality in the total UK Biobank cohort (**Table** **4**). Each 1‐year increase in MetAA1 and MetAA2 was associated with a 13% and 14% higher risk of CVD mortality, respectively, whereas MetAA1 and MetAA2 were associated with a 9% and 10% higher risk of cancer mortality, respectively. Cause‐specific mortality results were not shown for the ESTHER study due to low case numbers.

**Table 4:** Association of metabolomic age acceleration based on metabolomic biomarkers only (MetAA1) and based on both biomarkers and traditional risk factors (MetAA2) with 10‐year CVD and cancer mortality in the total UK Biobank.

MetAA / Study population	CVD mortality				Cancer mortality			
	N_total_	N_cases_	Crude HR per 1‐year MetAA increase ^a)^	Adjusted HR per 1‐year MetAA increase ^b)^	N_total_	N_cases_	Crude HR per 1‐year MetAA increase ^a)^	Adjusted HR per 1‐year MetAA increase ^b)^
**MetAA1**								
**Men aged 50–59 years**	40201	431	**1.30 (1.27–1.34)**	**1.13 (1.09–1.16)**	40201	870	**1.19 (1.17–1.21)**	**1.11 (1.09–1.13)**
**Men aged 60–69 years**	56111	1426	**1.37 (1.35–1.40)**	**1.18 (1.16–1.20)**	56111	2831	**1.22 (1.20–1.24)**	**1.13 (1.12–1.14)**
**Women aged 50–59 years**	50807	146	**1.34 (1.29–1.39)**	**1.15 (1.11–1.20)**	50807	878	**1.17 (1.14–1.20)**	**1.09 (1.07–1.11)**
**Women aged 60–69 years**	62025	578	**1.45 (1.41–1.49)**	**1.21 (1.18–1.25)**	62025	2135	**1.21 (1.19–1.23)**	**1.12 (1.10–1.14)**
**Total study population**	209144	2581	**1.30 (1.29–1.31)**	**1.13 (1.12–1.14)**	209144	6714	**1.17 (1.15–1.19)**	**1.09 (1.08–1.10)**
**MetAA2**								
**Men aged 50–59 years**	40201	431	**1.15 (1.11–1.18)**	**N.A**.^c)^	40201	870	**1.12 (1.10–1.14)**	**N.A**.^c)^
**Men aged 60–69 years**	56111	1426	**1.20 (1.18–1.22)**	**N.A**.^c)^	56111	2831	**1.15 (1.14–1.17)**	**N.A**.^c)^
**Women aged 50–59 years**	50807	146	**1.16 (1.12–1.21)**	**N.A**.^c)^	50807	878	**1.10 (1.08–1.12)**	**N.A**.^c)^
**Women aged 60–69 years**	62025	578	**1.23 (1.20–1.27)**	**N.A**.^c)^	62025	2135	**1.13 (1.11–1.15)**	**N.A**.^c)^
**Total study population**	209144	2581	**1.14 (1.13–1.15)**	**N.A**.^c)^	209144	6714	**1.10 (1.09–1.11)**	**N.A**.^c)^

Abbreviations: CI, confidence interval; CVD, cardiovascular disease; HR, hazard ratio; MetAA, metabolomic age acceleration; N.A., not applicable; SD, standard deviation.

Notes: Values printed in bold indicate statistical significance (*P *< 0.05).

^a)^ HRs for mortality were estimated per 1‐year increase in metabolomic age acceleration (MetAA1), reflecting the relative increase in mortality risk for each year that a person's metabolomic age exceeds their chronological age;

^b)^ The adjusted Cox proportional hazards regression model included the following covariates: chronological age, sex, education level, smoking status, physical activity, alcohol consumption, body mass index, treated dyslipidemia, hypertension, diabetes, cardiovascular disease, and cancer;

^c)^ As the MetaboMR clock2 already includes the traditional risk factors, it cannot be further adjusted for them.

The algorithms for the MetaboMR clocks have been made publicly available on GitHub (https://github.com/leipeng0825/metabolomics_10y_all_cause_mortality) to facilitate independent validation, and an interactive web calculator has been developed to enable clinical implementation (https://metabomrclock.shinyapps.io/metabomrclock/).

## 3 Discussion

By integrating high‐throughput NMR metabolomics profiling with conventional risk factors in two large prospective cohorts, we identified 68 circulating metabolomic biomarkers independently associated with 10‐year all‐cause mortality and validated them across both cohorts. These biomarkers mapped to key lipid metabolism and disease‐related pathways. Additionally, we developed sex‐ and age‐specific mortality prediction models, demonstrating consistent improvements in risk prediction across internal and external validation cohorts. The inclusion of metabolomic biomarkers significantly enhanced the mortality prediction, with the greatest benefit observed for short‐term (5‐year) mortality prediction. Moreover, we developed the MetaboMR clock1 and clock2, which captured the biological age well because each year of calculated age acceleration was associated with an 8% (MetaboMR clock1) or 9% (MetaboMR clock2) higher mortality in the external validation set than could be expected based on the chronological age alone.

After adjusting for conventional risk factors, our metabolome‐wide association analysis identified and externally validated key metabolomic biomarkers previously associated with mortality, including more than half of the 14 biomarkers reported by Deelen et al.^[^ ^4^ ^]^ and all four identified by Fischer et al.^[^ ^5^ ^]^ These include albumin, GlycA, citrate, histidine, and several lipid metabolites. From a mechanistic perspective, albumin is widely recognized as an indicator of systemic inflammation and nutritional status, reflecting overall protein homeostasis, which declines with ageing.^[^ ^26^ ^]^ Lower albumin levels have been associated with increased mortality risk, potentially due to their role in immune function, oxidative stress modulation, and chronic disease progression.^[^ ^26^, ^27^, ^28^ ^]^ GlycA has been widely validated as a robust marker of systemic inflammation, with strong associations with cardiovascular and autoimmune diseases,^[^ ^29^, ^30^, ^31^, ^32^ ^]^ contributing to disease‐related mortality.^[^ ^33^, ^34^, ^35^, ^36^ ^]^ Citrate, a key intermediate in the tricarboxylic acid (TCA) cycle, has been implicated in cancer metabolism, where altered energy utilization promotes tumor progression.^[^ ^37^, ^38^ ^]^ Similarly, lipids involved in cholesterol metabolism play a critical role in cardiovascular and metabolic disease‐related mortality.^[^ ^39^ ^]^ In contrast, histidine, another metabolite involved in central carbon metabolism, has been inversely associated with all‐cause mortality, possibly due to its role in antioxidant activity, nitric oxide production, and immune regulation.^[^ ^8^ ^]^

Using hierarchical clustering analysis, we identified four metabolic clusters linked to mortality, primarily composed of lipoprotein particles (HDL, IDL, LDL, and VLDL). KEGG‐based pathway analysis revealed associations with lipid metabolism, energy metabolism, and systemic inflammation, highlighting their role in cardiovascular disease, neurodegenerative diseases, and metabolic dysfunction. Additionally, the enrichment of pathways related to endocrine regulation (e.g., thyroid hormone synthesis and insulin signaling) suggests that metabolic perturbations may contribute to mortality through systemic effects.^[^ ^40^ ^]^ Beyond the shared clusters, sex‐ and age‐specific pathway enrichment was also observed. In younger men, enrichment of cancer metabolism, insulin resistance, and glucocorticoid pathways may underlie their higher midlife risk of metabolic and stress‐related disorders as well as malignancy.^[^ ^41^ ^]^ In older men, bile acid metabolism and viral infection pathways are consistent with age‐related changes in lipid regulation and immune function.^[^ ^42^, ^43^ ^]^ In younger women, enrichment of branched‐chain amino acid metabolism supports prior evidence linking elevated BCAAs to obesity and diabetes.^[^ ^39^ ^]^ In older women, pathways related to inflammation and microbial metabolism align with the role of inflammaging and gut dysbiosis in late‐life morbidity and mortality.^[^ ^44^, ^45^ ^]^ These subgroup‐specific patterns provide biological plausibility for the observed heterogeneity in metabolite‐mortality associations.

The observation that GlycA, the Omega‐6/Omega‐3 ratio, VLDL size, LA‐pct, valine, and albumin showed large positive or negative weights in two or more subgroups, further underscores the central role of inflammation, energy metabolism, and lipid regulation in ageing biology and mortality risk. While our study provides strong observational evidence, additional functional studies and Mendelian randomization analyses are required to establish causal relationships and to evaluate whether interventions targeting these pathways could modify ageing trajectories and mortality risk.

Integrating NMR‐based metabolomic data with clinical indicators and sociodemographic factors to develop a mortality risk score is a feasible approach.^[^ ^4^, ^5^, ^12^ ^]^ The improvement of the c‐statistic by adding metabolites to a traditional risk factor model in our study was consistent with those reported from general population samples by Fischer et al. (+0.031 for 5‐year mortality)^[^ ^5^ ^]^ and Deelen et al. (+0.065 and +0.040 for 5‐ and 10‐year mortality, respectively).^[^ ^4^ ^]^ Similarly, Lian et al. found that incorporating 40 NMR‐derived metabolomic biomarkers with conventional risk factors enhanced long‐term risk prediction (AUC increase from 0.813 to 0.833) in 110000 UK Biobank participants.^[^ ^12^ ^]^

Additionally, the categorical NRI in our study was 14.7% for 10‐year mortality and 19.1% for 5‐year mortality prediction in the total study population of the external validation cohort. The IDI was also statistically significant in these analyses. Comparable risk reclassification statistics have been reported by previous studies.^[^ ^4^, ^5^, ^12^ ^]^ However, these studies lacked external validation sets,^[^ ^5^, ^11^, ^12^, ^13^, ^14^, ^17^, ^20^ ^]^ which usually result in lower c‐statistics and reclassification statistics than in internal validation sets, limiting their generalizability.

Although some previous studies performed post hoc sex‐ or age‐stratified analyses,^[^ ^4^, ^6^, ^8^ ^]^ none has derived sex‐ and age‐specific biomarker models during model development. This distinction is critical, given biological differences in metabolic pathways across age and sex.^[^ ^22^, ^23^, ^24^, ^25^ ^]^ Thus, our results are novel and not directly comparable with previous research.

We further explored the utility of metabolomic biomarkers in predicting the biological age by developing two metabolomics‐based ageing clocks. The clock combining the selected metabolomic biomarkers and traditional risk factors (MetaboMR clock2) outperformed the clock based on metabolites only (MetaboMR clock1) and especially chronological age, when they were used to predict 10‐year all‐cause mortality alone without any other variables in the model. This shows that the MetaboMRclocks capture the biological age better than the chronological age. Surprisingly, the MetaboMRclock2 did not substantially outperform the MetaboMR clock1 in terms of the correlation with chronological age and the association of the metabolic age acceleration with all‐cause mortality. This underscores the utility of metabolomic biomarkers in estimating the biological age acceleration. Biologically, the blood metabolome might mirror the physiological alterations caused by the traditional risk factors so well that adding the traditional risk factors adds little additional risk information.

The observed 8% increase in 10‐year all‐cause mortality per year of biological age acceleration, as measured by MetaboMR clock1, indicates that each additional year of higher biological age compared to the chronological age is associated with an 8% higher risk of death over the following 10 years, highlighting its considerable predictive value. The risk for cancer mortality (9%) was comparable to that for all‐cause mortality, whereas the risk of death from CVD was particularly strongly increased (13% for each year of higher biological age), which underscores the importance of metabolites in pathophysiological processes leading to CVD, while their role as indicators of an increased risk for cancer mortality should also not be neglected.

The clocks developed in this study could serve as clinical endpoints to evaluate the efficacy of such interventions or as motivational tools to encourage individuals to adopt lifestyle changes that improve their metabolic profile (e.g., dietary or physical activity interventions). To facilitate real‐world applications, we developed a web‑based calculator for the MetaboMR clocks that requires only the input of the metabolomic measurements for the MetaboMR clock1, and additional basic questionnaire‐based mortality risk factor information for the MetaboMR clock2. This tool can readily be used in routine care.

Our age‐ and sex‐specific derivation approach of the MetaboMR clocks distincts the performance of the MetaboMR clocks from other metabolomic ageing clocks, previously developed in the UK Biobank. The clocks of Zhang et al.,^[^ ^11^ ^]^ Mutz et al.,^[^ ^14^ ^]^ Jia et al.^[^ ^15^ ^]^ and Hao et al.^[^ ^46^ ^]^ showed strong associations with ageing and mortality in the UK Biobank. However, these previous studies lacked external validation using large‐scale metabolomic data. Comparative evaluation of UK Biobank‐derived metabolomic ageing clocks in external cohorts remains an important direction for future research.

The strengths of our study include its prospective design, large sample size, extended follow‐up, and independent external validation. The use of the same standardized NMR metabolomics platform across both cohorts ensured consistency of the measurements. However, certain limitations must be acknowledged. The NMR platform primarily quantifies lipid metabolites, limiting assessment of other metabolic pathways.^[^ ^47^ ^]^ As an observational study, causality cannot be established, though several of Hill's criteria (temporality, strength, consistency, and biological plausibility) are met.^[^ ^48^ ^]^ Furthermore, metabolomics data were collected only at baseline, and studies with repeated measurements are needed to assess how dynamic changes in metabolite profiles are related to mortality risk.^[^ ^25^, ^49^, ^50^ ^]^ Despite differences in sample type (plasma in UK Biobank vs serum in ESTHER), our results remained highly concordant, emphasizing the reproducibility of identified biomarkers. This aligns with findings from Deelen et al., who observed consistent biomarker‐mortality associations across 12 cohorts, regardless of sample type.^[^ ^4^ ^]^ Moreover, although the UK Biobank had a higher baseline prevalence of treated dyslipidemia than the ESTHER study, sensitivity analyses excluding participants receiving lipid‑lowering therapy at baseline produced materially unchanged results, implying that pharmacologic lipid modulation does not have a relevant impact on the performance of the MetaboMR clocks. Finally, as the models were derived and tested in a mostly Caucasian population from Western Europe, additional validation across diverse populations is needed.

In conclusion, this study provides robust evidence that incorporating metabolites alongside conventional risk factors enhances mortality prediction, and that the subsequently derived MetaboMR clock1, which is based solely on metabolomic biomarkers, and MetaboMR clock2, which incorporates both biomarkers and traditional mortality risk factors, could serve as useful measures for assessing the biological age. By deriving age‐ and sex‐specific models, we highlight the potential of metabolomics for enhancing risk stratification and precision medicine. As NMR‐based metabolomics is a cost‐effective, scalable tool for risk assessment, its translation into clinical settings is possible.^[^ ^51^ ^]^ Future research should integrate metabolomics with other omics technologies (multi‐omics) to further refine personalized risk prediction models.

## 4 Experimental Section

### Study Design and Populations

This study used data from the UK Biobank as the derivation cohort and the German ESTHER study as the external validation cohort. The UK Biobank is a large‐scale prospective cohort study involving 502366 participants aged 37–73 years, recruited between March 13, 2006, and October 1, 2010. Participants were selected from 22 assessment centers located across England, Scotland, and Wales, all situated within a 25‐mile (≈40‐km) radius of the respective centers.^[^ ^52^, ^53^ ^]^ Baseline data collection included extensive health, lifestyle, and biomarker information.^[^ ^52^, ^54^ ^]^

The ESTHER study (German name: Epidemiologische Studie zu Chancen der Verhütung Früherkennung und optimierten Therapie chronischer Erkrankungen in der älteren Bevölkerung) is a population‐based cohort study conducted in Saarland, Germany,^[^ ^55^, ^56^, ^57^ ^]^ and is utilized as the external validation set. In brief, 9940 participants aged 50–75 years were recruited by general practitioners (GPs) during routine health check‐ups between 1 July 2000 and 1 June 2002.

Participants were selected according to predefined inclusion and exclusion criteria in both the UK Biobank and ESTHER cohorts (see flow‐chart, Figure S1, Supporting Information). In the UK Biobank, individuals not selected for plasma metabolomics analysis (*n* = 228017) and those outside the baseline age range of 50–69 years (*n* = 65205) were excluded, resulting in a final cohort of 209144 participants. This cohort was randomly divided into a derivation set (70%, *n* = 146400) and an internal test set (30%, *n* = 62744) and stratified by sex and age. Finally, both the derivation and internal test set were stratified by sex and age groups (50–59 and 60–69 years). For external validation, the ESTHER cohort was processed using the same inclusion and exclusion criteria, yielding 6820 eligible participants, who were similarly stratified into four subgroups.

### Metabolomics Profiling

An NMR platform (Nightingale Health Ltd., Helsinki, Finland)^[^ ^58^ ^]^ was employed to quantify circulating metabolomic biomarkers at baseline from ethylenediaminetetraacetic acid (EDTA) plasma samples collected from study participants in the UK Biobank and from serum samples in the ESTHER study. Nightingale Health's NMR technology ensures high reproducibility and reliability, supported by a certified quality management system in accordance with international standards such as EN ISO 13485:2016 and SFS‐EN ISO/IEC 17025. This method enables the simultaneous quantification of 250 metabolomic biomarkers, including lipids, lipoprotein subclasses, apolipoproteins, fatty acids, amino acids, ketone bodies, glycolysis related metabolites, albumin, the kidney function biomarker creatinine, and the inflammation biomarker glycoprotein acetyls (GlycA). Details of the experimentation and applications of the NMR metabolomics platform have been described previously.^[^ ^51^, ^59^ ^]^ Glycerol was excluded due to a proportion of missing values exceeding 10% in both cohorts. The names of the metabolomic biomarkers are indicated by representative abbreviations, and the full names and distributions are detailed in Table S10 (Supporting Information).

### Assessment of Mortality

In the UK Biobank, mortality was ascertained via linkage to national health registries (NHS Digital for England and Wales, and the NHS Central Register for Scotland),^[^ ^52^ ^]^ with follow‐up available until November 30, 2022. In the ESTHER study, mortality was determined through linkage with local population registries in Saarland, Germany,^[^ ^60^ ^]^ and was complete through December 31, 2022. In both cohorts, causes of death were coded according to the International Classification of Diseases, 10th Revision (ICD‐10). CVD mortality was defined as codes I00–I99, and cancer mortality as codes C00–C97, excluding non‐melanoma skin cancer (C44). Participants were followed from baseline until the date of death, date lost to follow‐up, or completion of 10 years of follow‐up. Follow‐up for mortality was virtually complete in both cohorts.

### Assessment of Covariates

In both the UK Biobank and the ESTHER study, baseline demographic characteristics, lifestyle factors, and medical history were collected using standardized questionnaires. In the UK Biobank, data and blood samples were collected by study staff at assessment centers and verified through linkage to national health records to ensure accuracy and completeness. In the ESTHER study, trained GPs conducted standardized health check‐ups, collected blood samples, and documented current medication prescriptions and chronic diseases.

Age was recorded in years, and sex was self‐reported. Education level was categorized based on the highest qualification attained. Smoking status was classified as never, former, or current smoker. Physical activity was assessed using a structured self‐administered questionnaire in the ESTHER study and the International Physical Activity Questionnaire (IPAQ) in the UK Biobank, which captured frequency, duration, and intensity. Alcohol consumption was recorded in grams of alcohol per day. BMI was calculated as weight in kilograms divided by height in meters squared (kg/m^2^). Medical history, including treated dyslipidemia, hypertension, diabetes, cardiovascular disease, and cancer, was based on self‐reported physician diagnoses and medication use. Diastolic and systolic blood pressure measurements ≥90 mm Hg and ≥140 mm Hg, respectively, were used to complement diagnoses of hypertension. The diagnosis of diabetes was based on self‐report, use of glucose‐lowering drugs, or HbA_1c_ levels ≥6.5%, which were measured from whole‐blood samples using high‐performance liquid chromatography on the Variant II analyzer (Bio‐Rad). Table 1 presents the detailed descriptions for each covariate.

### Statistical Analysis

Missing values for all variables were imputed using a random forest‐based imputation method.^[^ ^61^ ^]^ This iterative approach predicts missing values by constructing random forest models for each variable with missing data, leveraging observed values from other variables as predictors.^[^ ^61^ ^]^ All metabolomic biomarker concentrations were log‐transformed and standardized to z‐scores by mean‐centering and scaling to a SD of 1, allowing for direct comparisons of effect sizes across metabolomic biomarkers.^[^ ^62^, ^63^ ^]^

In the total study population of both cohorts, Cox proportional hazards models, adjusted for conventional risk factors (age, sex, education level, smoking status, physical activity, alcohol consumption, BMI, treated dyslipidemia, hypertension, diabetes, cardiovascular disease, and cancer), were used to determine hazard ratios (HRs) per 1‐SD increment and 95% confidence intervals (CIs) for the associations of all 249 metabolomic biomarkers with 10‐year all‐cause mortality. To account for multiple testing, the Benjamini‐Hochberg procedure^[^ ^64^ ^]^ was applied to keep the FDR at <5%. Schoenfeld residuals were utilized to examine the proportional hazards assumption.^[^ ^65^, ^66^ ^]^ Metabolite clusters among the validated biomarkers were searched using WGCNA, which constructs a scale‐free network based on hierarchical clustering.^[^ ^67^, ^68^ ^]^ Furthermore, metabolic pathway analysis based on the KEGG database (http://www.kegg.jp/ or http://www.genome.jp/kegg/)^[^ ^69^ ^]^ was used for biological interpretation of the associations of validated metabolites with mortality.

The LASSO regularized Cox regression model, including the covariates of the traditional risk factor model, with ten‐fold cross‐validation was used to identify promising metabolomic biomarkers for predicting 10‐year all‐cause mortality within age‐ (50–59/60–69 years) and sex‐specific strata from the derivation set.^[^ ^70^, ^71^ ^]^ A bootstrap LASSO approach was employed,^[^ ^72^ ^]^ involving the generation of 1000 resampled datasets, with the LASSO procedure applied to each. Metabolomic biomarkers selected by the LASSO procedure in at least 95% of the resampled datasets were retained for inclusion in the final model.

Three metrics were used to compare and evaluate the improvement in predictive performance between models that included metabolites and those based solely on traditional risk factors: (1) Harrell's c‐statistic,^[^ ^73^ ^]^ (2) NRI^[^ ^74^ ^]^ and (3) IDI.^[^ ^75^ ^]^ The Delong test was used to compare the c‐statistics.^[^ ^76^ ^]^ The categorical NRI was evaluated across three risk categories (≤ 5%, >5%–10%, >10%) based on conventional risk factors and selected metabolomic biomarkers.^[^ ^77^ ^]^ Model calibration was assessed by plotting observed versus predicted mortality probabilities using a nonparametric locally weighted scatterplot smoothing (loess) approach.^[^ ^78^ ^]^ In an additional sensitivity analysis, the model performance metrics were assessed for short‐term mortality prediction (5 years).

To construct two age‐ and sex‐specific MetaboMR clocks, the LASSO‐selected mortality‐associated metabolites from the derivation set were used as predictors in elastic net regression models,^[^ ^79^ ^]^ with chronological age as the dependent variable. The MetaboMR clock1 was based on the selected metabolites only and the MetaboMR clock2 combined them with the traditional risk factors. To improve the comparability with the chronological age, the raw metabolomic age was subsequently calibrated using the intercept (*β_0_*) and slope (*β_1_*) from a linear regression model with chronological age as the dependent variable and the raw metabolomic age as the independent variable. The calibrated metabolomic age was then defined as: MetaboMR clock *= β_0_ + β_1_ ×* raw metabolomic age. The distribution of the MetaboMR clocks was plotted against the distribution of the chronological age in histograms to show how well the two ageing metrics align. Subsequently, a Shiny‑based web calculator for the MetaboMR clocks was developed for simplified use of the algorithms in clinical routine.

The performance of these clocks was evaluated by examining their correlations with the chronological age using Pearson correlation coefficients. To evaluate the discriminatory ability of the MetaboMR clocks for mortality prediction, c‐statistics for 10‐year all‐cause mortality were also calculated in comparison with the c‐statistic of the chronological age. Subsequently, HRs with 95%CI were assessed for the associations of each year of the metabolomic age acceleration (MetAA) with 10‐year all‐cause mortality. The MetAA is the residual from a linear regression model, in which the MetaboMR clock was the dependent and the chronological age was the independent variable. These residuals represent the portion of the difference between the MetaboMR clocks and chronological age that is not explained by the chronological age, or in other words, the MetAAs reflect inter‐individual variation in biological ageing beyond what is expected for a given inter‐individual variation of the chronological age. The distribution of the MetAAs was visualized using violin plots. At last, associations of the MetAAs with all‐cause mortality, CVD mortality, and cancer mortality were assessed with proportional hazards regression models.

In a sensitivity analysis, participants on lipid‑lowering therapy at baseline were excluded, and the models’ discrimination (c‑statistics) and associations of MetAAs with mortality were re‐assessed.

The dataset was created using SAS software, version 9.4 (Cary, North Carolina, USA). Continuous variables are reported as mean (SD) and categorical variables as count (percent). Exact sample sizes (n) for each cohort and analysis are given in Table 1 and Figure S1 (Supporting Information). All statistical tests were two‐sided, with significance defined as *P *< 0.05, and were performed in R, version 4.3.1. The main R packages included ‘*missRanger* 2.1.5′ for random forest imputation (100 trees and 5 iterations), ‘*survival* 3.5‐7′ for Cox regression and model calibration, ‘*WGCNA* 1.72‐5′ for metabolite cluster derivation, ‘*glmnet* 4.1‐8′ for LASSO and elastic net regressions, ‘*pROC* 1.18.5′ for discrimination, ‘*nricens* 1.6′ for reclassification, ‘*ggplot2* 3.5.1′ for visualization, and ‘*shiny* 1.8.1.1′ for development of the practical web calculator for MetaboMR clocks.

### Ethics Approval and Consent to Participate

The UK Biobank was approved by the Northwest Multi‐center Research Ethics Committee (MREC) as a Research Tissue Bank (RTB) approval (2021: 21/NW/0157), and all participants provided written informed consent. The ESTHER study was approved by the ethics committee of the Heidelberg Medical Faculty of Heidelberg University and the State Medical Board of Saarland, Germany. All participants provided written informed consent.

## Conflict of Interest

The authors declare no conflict of interest

## Author Contributions

B.S. designed the study. L.P. conducted data analyses and drafted the initial manuscript. B.S. made major revisions. H.B., B.H., and R.X. critically reviewed and revised the manuscript, contributing to data interpretation and discussion. All authors approved the final manuscript. L.P. and B.S. are responsible for the integrity of the work, ensuring accuracy and completeness are properly addressed.

## Supporting information



Supporting Information

## Data Availability

We are unable to share the raw NMR data from Nightingale Health Ltd., as the company holds the proprietary rights. This study used UK Biobank data, publicly accessible to researchers via an open application at https://www.ukbiobank.ac.uk/register‐apply/. The ESTHER study data (http://esther.dkfz.org/esther/) cannot be publicly shared due to informed consent restrictions but if investigators wishing to use ESTHER dataset generated and/or analyzed during the current study are asked to submit a brief description of the proposed project to the corresponding author.
