# Supplementary material for: Development of Age‐ and Sex‐Specific Metabolomics‐Based Biological Ageing Clocks for 10‐Year Mortality Prediction
Source: Adv Sci (Weinh). 2025 Oct 15;13(1):e10189. doi: 10.1002/advs.202510189 (PMC12767096; doi:10.1002/advs.202510189)
Supplement: Supplementary file 1 — Supporting Information [file ADVS-13-e10189-s001.docx]

**Supporting Information**

**Development of Age- and Sex-Specific Metabolomics-Based Biological Ageing Clocks for 10-Year Mortality Prediction**

**Table of contents**

**Table S1.** Longitudinal association between traditional risk factors and 10-year all-cause mortality in the UK Biobank and ESTHER study 1

**Table S2.** Longitudinal associations of all 249 metabolites with 10-year all-cause mortality in the total study population of the UK Biobank 3

**Table S3.** Longitudinal associations of 224 selected metabolites with 10-year all-cause mortality in the total study population of the ESTHER study 8

**Table S4.** Summary of potential KEGG pathways associated with 68 validated metabolic biomarkers identified from WGCNA-derived metabolite clusters 13

**Table S5.** *β*-coefficients of the variables included in the risk scores for 10-year all-cause mortality prediction derived in 70% of the UK Biobank and used with the same values in the internal and external validation 15

**Table S6.** C-statistics of prediction models for 10- and 5-year all-cause mortality in the UK Biobank and ESTHER cohorts, excluding participants on lipid‑lowering therapy 17

**Table S7.** Reclassification improvements in 10- and 5-year mortality prediction by adding metabolomic biomarkers to conventional risk models: evidence from the UK Biobank and ESTHER study 18

**Table S8.** Equations used to calculate the metabolomics-based mortality risk prediction (MetaboMR) clocks 1 and 2 19

**Table S9.** Association of metabolomic age acceleration based on metabolomic biomarkers only (MetAA1) and based on both biomarkers and traditional risk factors (MetAA2) with 10-year all-cause mortality, excluding participants on lipid‑lowering therapy 21

**Table S10.** Abbreviations, full names, classes and distributions of 249 metabolomic biomarkers in the UK Biobank and ESTHER study 22

**Figure S1.** Flow chart of study population from the UK Biobank and ESTHER study 36

**Figure S2.** Topological overlap map of metabolomic biomarkers 37

**Figure S3.** Effect sizes of standardized *β*-coefficients of selected biomarkers used to derive mortality risk scores in the training set (70% of the UK Biobank) 38

**Figure S4.** Spearman correlations between the conventional risk factors and 26 metabolomic biomarkers in the derivation set (70% UK Biobank) 39

**Figure S5.** Improved mortality prediction accuracy with identified metabolomic biomarkers in sex- and age-specific groups, as well as the total study population 40

**Figure S6.** Calibration curves of the models only including conventional risk factors and their combinations with the metabolites for risk prediction in the internal and external validation cohorts 42

**Figure S7.** Linear correlation between chronological age and the metabolomic age based on 26 selected metabolomic biomarkers only (metabolomics-based mortality risk clock 1 (MetaboMR clock1) 44

**Figure S8.** Linear correlation between chronological age and metabolomic age as a metabolomics-based mortality risk clock (MetaboMR clock2) derived from the 26 selected metabolomic biomarkers and conventional risk factors 45

**Figure S9.** Violin plots of the distribution of metabolomic age acceleration (MetAA) derived from (a) MetaboMR clock1 and (b) from MetaboMR clock2 in the UK Biobank and ESTHER cohorts 46

**Supplementary References** 47

# Table S1. Longitudinal association between traditional risk factors and 10-year all-cause mortality in the UK Biobank and ESTHER study.

| **Baseline Characteristics** | **UK Biobank (209,144 participants)** | | |  | **ESTHER Study (6,820 participants)** | | |
| --- | --- | --- | --- | --- | --- | --- | --- |
|  | ***N* _death_ (%)** | **HR (95% CI) ^a^** | ***P*-value** |  | ***N* _death_ (%)** | **HR (95% CI) ^a^** | ***P*-value** |
| **Age** |  |  |  |  |  |  |  |
| 50–59 years old | 3,056 (3.4) | Ref. |  |  | 215 (5.3) | Ref. |  |
| 60–69 years old | 9,291 (7.9) | 2.31 (2.22–2.40) | <0.001 |  | 589 (14.5) | 2.09 (1.81–2.42) | <0.001 |
| **Sex** |  |  |  |  |  |  |  |
| Women | 4,852 (4.3) | Ref. |  |  | 335 (8.9) | Ref. |  |
| Men | 7,495 (7.8) | 1.71 (1.65–1.78) | <0.001 |  | 469 (15.3) | 1.72 (1.47–2.02) | <0.001 |
| **Education level** |  |  |  |  |  |  |  |
| Medium or low | 8,979 (6.0) | Ref. |  |  | 622 (12.3) | Ref. |  |
| High | 3,368 (5.7) | 0.95 (0.92–0.99) | 0.036 |  | 182 (10.4) | 0.99 (0.85–1.18) | 0.849 |
| **Smoking status** |  |  |  |  |  |  |  |
| Never smoker | 4,625 (4.2) | Ref. |  |  | 312 (9.1) | Ref. |  |
| Former smoker | 5,402 (6.9) | 1.36 (1.30–1.42) | <0.001 |  | 284 (12.9) | 1.20 (1.01–1.43) | 0.041 |
| Current smoker | 2,320 (11.7) | 2.66 (2.53–2.80) | <0.001 |  | 208 (17.3) | 2.15 (1.78–2.59) | <0.001 |
| **Physical activity** |  |  |  |  |  |  |  |
| Low | 3,198 (7.3) | Ref. |  |  | 213 (15.8) | Ref. |  |
| Moderate | 4,849 (5.6) | 0.82 (0.79–0.86) | <0.001 |  | 378 (12.2) | 0.78 (0.66–0.92) | 0.004 |
| High | 4,300 (5.4) | 0.78 (0.74–0.81) | <0.001 |  | 213 (8.9) | 0.63 (0.52–0.77) | <0.001 |
| **Alcohol consumption** |  |  |  |  |  |  |  |
| Abstainer | 3,281 (5.1) | Ref. |  |  | 242 (10.8) | Ref. |  |
| Low | 4,493 (5.4) | 1.08 (1.01–1.17) | 0.011 |  | 496 (12.3) | 1.12 (1.02–1.23) | 0.037 |
| Medium or high | 4,573 (7.4) | 1.39 (1.33–1.46) | <0.001 |  | 66 (12.0) | 1.04 (0.77–1.35) | 0.919 |
| **Body mass index** |  |  |  |  |  |  |  |
| < 21.5 kg/m^2^ | 947 (7.0) | 1.52 (1.41–1.64) | <0.001 |  | 50 (14.2) | 1.66 (1.20–2.30) | 0.002 |
| ≥ 21.5 – < 25 kg/m^2^ | 2,578 (4.9) | Ref. |  |  | 154 (10.0) | Ref. |  |
| ≥ 25 – < 30 kg/m^2^ | 5,085 (5.6) | 0.94 (0.89–0.98) | 0.007 |  | 350 (11.0) | 0.92 (0.76–1.11) | 0.378 |
| ≥ 30 – < 35 kg/m^2^ | 2,475 (6.5) | 0.98 (0.93–1.04) | 0.576 |  | 179 (13.3) | 1.03 (0.82–1.29) | 0.801 |
| ≥ 35 kg/m^2^ | 1,262 (8.7) | 1.30 (1.21–1.39) | <0.001 |  | 71 (18.0) | 1.51 (1.13–2.02) | 0.006 |
| **Treated dyslipidemia** |  |  |  |  |  |  |  |
| No | 8,072 (4.9) | Ref. |  |  | 692 (11.3) | Ref. |  |
| Yes | 4,275 (9.4) | 0.98 (0.93–1.02) | 0.319 |  | 112 (15.7) | 0.98 (0.80–1.21) | 0.858 |
| **Hypertension** |  |  |  |  |  |  |  |
| No | 3,515 (4.1) | Ref. |  |  | 230 (8.6) | Ref. |  |
| Yes | 8,832 (7.2) | 1.23 (1.18–1.29) | <0.001 |  | 574 (13.8) | 1.25 (1.06–1.47) | 0.009 |
| **Diabetes** |  |  |  |  |  |  |  |
| No | 9,844 (5.3) | Ref. |  |  | 595 (10.2) | Ref. |  |
| Yes | 2,503 (11.6) | 1.50 (1.43–1.57) | <0.001 |  | 209 (21.7) | 1.66 (1.41–1.96) | <0.001 |
| **Cardiovascular disease** |  |  |  |  |  |  |  |
| No | 10,130 (5.2) | Ref. |  |  | 677 (10.7) | Ref. |  |
| Yes | 2,217 (14.4) | 1.75 (1.66–1.84) | <0.001 |  | 127 (25.3) | 1.68 (1.37–2.06) | <0.001 |
| **Cancer** |  |  |  |  |  |  |  |
| No | 10,040 (5.3) | Ref. |  |  | 698 (11.0) | Ref. |  |
| Yes | 2,307 (12.5) | 2.31 (2.20–2.41) | <0.001 |  | 106 (22.5) | 2.12 (1.73–2.61) | <0.001 |

**a)** Cox proportional hazards regression model included all covariates: age, sex, education level, smoking status, physical activity, alcohol consumption, body mass index, treated dyslipidemia, hypertension, diabetes, cardiovascular disease, and cancer. Proportional hazards assumption was evaluated using Schoenfeld residuals.^[1-2]^ CI, confidence interval; HR, hazard ratio.

# Table S2. Longitudinal associations of all 249 metabolites with 10-year all-cause mortality in the total study population of the UK Biobank.

| **Metabolic biomarkers** | **Estimate** | **SE** | **HR (95% CI) ^a^** | ***P*-value** | **FDR-adjusted *P*-value** |
| --- | --- | --- | --- | --- | --- |
| Albumin | -0.134 | 0.005 | 0.87 (0.87–0.88) | <0.001 | <0.001 |
| S-HDL-CE | -0.209 | 0.009 | 0.81 (0.80–0.83) | <0.001 | <0.001 |
| M-LDL-TG-pct | 0.182 | 0.009 | 1.20 (1.18–1.22) | <0.001 | <0.001 |
| S-HDL-C | -0.196 | 0.010 | 0.82 (0.81–0.84) | <0.001 | <0.001 |
| Omega-3-pct | -0.193 | 0.009 | 0.82 (0.81–0.84) | <0.001 | <0.001 |
| S-HDL-P | -0.196 | 0.010 | 0.82 (0.81–0.84) | <0.001 | <0.001 |
| L-LDL-TG-pct | 0.178 | 0.009 | 1.19 (1.17–1.22) | <0.001 | <0.001 |
| XL-HDL-FC | 0.188 | 0.010 | 1.21 (1.18–1.23) | <0.001 | <0.001 |
| IDL-CE-pct | -0.164 | 0.009 | 0.85 (0.83–0.86) | <0.001 | <0.001 |
| Omega-6/Omega-3 | 0.166 | 0.009 | 1.18 (1.16–1.20) | <0.001 | <0.001 |
| XL-HDL-PL | 0.185 | 0.010 | 1.20 (1.18–1.23) | <0.001 | <0.001 |
| Omega-3 | -0.175 | 0.010 | 0.84 (0.82–0.86) | <0.001 | <0.001 |
| HDL-size | 0.184 | 0.010 | 1.20 (1.18–1.23) | <0.001 | <0.001 |
| XS-VLDL-PL-pct | 0.171 | 0.010 | 1.19 (1.16–1.21) | <0.001 | <0.001 |
| S-HDL-CE-pct | -0.142 | 0.008 | 0.87 (0.85–0.88) | <0.001 | <0.001 |
| Unsaturation | -0.163 | 0.010 | 0.85 (0.83–0.87) | <0.001 | <0.001 |
| XL-HDL-L | 0.178 | 0.011 | 1.20 (1.17–1.22) | <0.001 | <0.001 |
| M-LDL-C | -0.168 | 0.010 | 0.85 (0.83–0.86) | <0.001 | <0.001 |
| S-HDL-L | -0.160 | 0.010 | 0.85 (0.84–0.87) | <0.001 | <0.001 |
| XL-HDL-C-pct | -0.079 | 0.005 | 0.92 (0.92–0.93) | <0.001 | <0.001 |
| Acetate | 0.073 | 0.004 | 1.08 (1.07–1.08) | <0.001 | <0.001 |
| M-LDL-FC | -0.168 | 0.010 | 0.85 (0.83–0.86) | <0.001 | <0.001 |
| M-LDL-L | -0.160 | 0.010 | 0.85 (0.84–0.87) | <0.001 | <0.001 |
| M-LDL-CE | -0.162 | 0.010 | 0.85 (0.83–0.87) | <0.001 | <0.001 |
| M-LDL-PL | -0.163 | 0.010 | 0.85 (0.83–0.87) | <0.001 | <0.001 |
| LDL-C | -0.159 | 0.010 | 0.85 (0.84–0.87) | <0.001 | <0.001 |
| LDL-CE | -0.159 | 0.010 | 0.85 (0.84–0.87) | <0.001 | <0.001 |
| GlycA | 0.148 | 0.009 | 1.16 (1.14–1.18) | <0.001 | <0.001 |
| L-VLDL-TG-pct | -0.093 | 0.006 | 0.91 (0.90–0.92) | <0.001 | <0.001 |
| Val | -0.156 | 0.010 | 0.86 (0.84–0.87) | <0.001 | <0.001 |
| XL-HDL-P | 0.163 | 0.010 | 1.18 (1.15–1.20) | <0.001 | <0.001 |
| S-HDL-PL-pct | 0.146 | 0.009 | 1.16 (1.14–1.18) | <0.001 | <0.001 |
| DHA | -0.161 | 0.010 | 0.85 (0.83–0.87) | <0.001 | <0.001 |
| bOHbutyrate | 0.112 | 0.007 | 1.12 (1.10–1.13) | <0.001 | <0.001 |
| Acetone | 0.082 | 0.005 | 1.09 (1.07–1.10) | <0.001 | <0.001 |
| XL-HDL-C | 0.159 | 0.011 | 1.17 (1.15–1.20) | <0.001 | <0.001 |
| S-LDL-C | -0.153 | 0.010 | 0.86 (0.84–0.88) | <0.001 | <0.001 |
| Clinical-LDL-C | -0.150 | 0.010 | 0.86 (0.84–0.88) | <0.001 | <0.001 |
| VLDL-size | -0.142 | 0.010 | 0.87 (0.85–0.88) | <0.001 | <0.001 |
| L-LDL-CE | -0.152 | 0.010 | 0.86 (0.84–0.88) | <0.001 | <0.001 |
| S-LDL-C-pct | -0.035 | 0.002 | 0.97 (0.96–0.98) | <0.001 | <0.001 |
| LDL-L | -0.147 | 0.010 | 0.86 (0.85–0.88) | <0.001 | <0.001 |
| S-LDL-FC | -0.150 | 0.010 | 0.86 (0.84–0.88) | <0.001 | <0.001 |
| IDL-C-pct | -0.128 | 0.009 | 0.88 (0.87–0.90) | <0.001 | <0.001 |
| L-LDL-C | -0.150 | 0.010 | 0.86 (0.84–0.88) | <0.001 | <0.001 |
| PUFA | -0.143 | 0.010 | 0.87 (0.85–0.88) | <0.001 | <0.001 |
| IDL-PL-pct | 0.134 | 0.009 | 1.14 (1.12–1.16) | <0.001 | <0.001 |
| LDL-PL | -0.147 | 0.010 | 0.86 (0.85–0.88) | <0.001 | <0.001 |
| Total-BCAA | -0.143 | 0.010 | 0.87 (0.85–0.88) | <0.001 | <0.001 |
| LDL-FC | -0.148 | 0.010 | 0.86 (0.84–0.88) | <0.001 | <0.001 |
| S-LDL-CE | -0.145 | 0.010 | 0.87 (0.85–0.88) | <0.001 | <0.001 |
| Leu | -0.141 | 0.010 | 0.87 (0.85–0.89) | <0.001 | <0.001 |
| S-HDL-C-pct | -0.122 | 0.009 | 0.89 (0.87–0.90) | <0.001 | <0.001 |
| S-HDL-PL | -0.134 | 0.010 | 0.87 (0.86–0.89) | <0.001 | <0.001 |
| XL-HDL-CE | 0.148 | 0.011 | 1.16 (1.14–1.18) | <0.001 | <0.001 |
| DHA-pct | -0.130 | 0.009 | 0.88 (0.86–0.89) | <0.001 | <0.001 |
| L-LDL-L | -0.138 | 0.010 | 0.87 (0.85–0.89) | <0.001 | <0.001 |
| S-LDL-L | -0.137 | 0.010 | 0.87 (0.85–0.89) | <0.001 | <0.001 |
| Acetoacetate | 0.080 | 0.006 | 1.08 (1.07–1.10) | <0.001 | <0.001 |
| S-LDL-PL-pct | 0.122 | 0.009 | 1.13 (1.11–1.15) | <0.001 | <0.001 |
| L-LDL-PL | -0.138 | 0.011 | 0.87 (0.85–0.89) | <0.001 | <0.001 |
| non-HDL-C | -0.130 | 0.010 | 0.88 (0.86–0.90) | <0.001 | <0.001 |
| M-VLDL-L | -0.123 | 0.010 | 0.88 (0.87–0.90) | <0.001 | <0.001 |
| M-LDL-CE-pct | -0.039 | 0.003 | 0.96 (0.95–0.97) | <0.001 | <0.001 |
| L-LDL-FC | -0.134 | 0.011 | 0.87 (0.86–0.89) | <0.001 | <0.001 |
| IDL-TG-pct | 0.123 | 0.010 | 1.13 (1.11–1.15) | <0.001 | <0.001 |
| L-HDL-FC | 0.135 | 0.011 | 1.15 (1.12–1.17) | <0.001 | <0.001 |
| M-LDL-C-pct | -0.028 | 0.002 | 0.97 (0.96–0.98) | <0.001 | <0.001 |
| Creatinine | 0.073 | 0.006 | 1.08 (1.06–1.09) | <0.001 | <0.001 |
| S-HDL-FC | -0.120 | 0.010 | 0.89 (0.87–0.90) | <0.001 | <0.001 |
| M-LDL-P | -0.126 | 0.010 | 0.88 (0.86–0.90) | <0.001 | <0.001 |
| M-VLDL-TG | -0.117 | 0.010 | 0.89 (0.87–0.91) | <0.001 | <0.001 |
| S-LDL-CE-pct | -0.043 | 0.004 | 0.96 (0.95–0.96) | <0.001 | <0.001 |
| L-LDL-CE-pct | -0.028 | 0.002 | 0.97 (0.96–0.98) | <0.001 | <0.001 |
| L-VLDL-TG | -0.114 | 0.010 | 0.89 (0.88–0.91) | <0.001 | <0.001 |
| His | -0.114 | 0.010 | 0.89 (0.88–0.91) | <0.001 | <0.001 |
| PUFA-pct | -0.101 | 0.009 | 0.90 (0.89–0.92) | <0.001 | <0.001 |
| Omega-6 | -0.113 | 0.010 | 0.89 (0.88–0.91) | <0.001 | <0.001 |
| Total-CE | -0.120 | 0.011 | 0.89 (0.87–0.91) | <0.001 | <0.001 |
| LA | -0.112 | 0.010 | 0.89 (0.88–0.91) | <0.001 | <0.001 |
| Total-C | -0.117 | 0.010 | 0.89 (0.87–0.91) | <0.001 | <0.001 |
| S-LDL-PL | -0.112 | 0.010 | 0.89 (0.88–0.91) | <0.001 | <0.001 |
| L-HDL-L | 0.122 | 0.011 | 1.13 (1.11–1.15) | <0.001 | <0.001 |
| L-HDL-PL | 0.120 | 0.011 | 1.13 (1.10–1.15) | <0.001 | <0.001 |
| L-VLDL-L | -0.106 | 0.010 | 0.90 (0.88–0.92) | <0.001 | <0.001 |
| M-VLDL-P | -0.111 | 0.010 | 0.90 (0.88–0.91) | <0.001 | <0.001 |
| L-HDL-P | 0.119 | 0.011 | 1.13 (1.10–1.15) | <0.001 | <0.001 |
| S-HDL-FC-pct | 0.106 | 0.010 | 1.11 (1.09–1.13) | <0.001 | <0.001 |
| M-VLDL-PL | -0.108 | 0.010 | 0.90 (0.88–0.92) | <0.001 | <0.001 |
| PUFA/MUFA | -0.101 | 0.010 | 0.90 (0.89–0.92) | <0.001 | <0.001 |
| XL-HDL-FC-pct | -0.104 | 0.010 | 0.90 (0.88–0.92) | <0.001 | <0.001 |
| L-HDL-C | 0.117 | 0.011 | 1.12 (1.10–1.15) | <0.001 | <0.001 |
| XL-VLDL-FC-pct | 0.109 | 0.011 | 1.12 (1.09–1.14) | <0.001 | <0.001 |
| Total-P | -0.109 | 0.011 | 0.90 (0.88–0.92) | <0.001 | <0.001 |
| LDL-P | -0.108 | 0.011 | 0.90 (0.88–0.92) | <0.001 | <0.001 |
| S-LDL-P | -0.106 | 0.010 | 0.90 (0.88–0.92) | <0.001 | <0.001 |
| M-VLDL-FC | -0.105 | 0.010 | 0.90 (0.88–0.92) | <0.001 | <0.001 |
| SFA-pct | 0.091 | 0.009 | 1.10 (1.08–1.11) | <0.001 | <0.001 |
| Total-L | -0.099 | 0.010 | 0.91 (0.89–0.92) | <0.001 | <0.001 |
| L-HDL-CE | 0.110 | 0.011 | 1.12 (1.09–1.14) | <0.001 | <0.001 |
| ApoB | -0.102 | 0.010 | 0.90 (0.88–0.92) | <0.001 | <0.001 |
| L-HDL-TG | 0.089 | 0.009 | 1.09 (1.07–1.11) | <0.001 | <0.001 |
| Total-FC | -0.104 | 0.011 | 0.90 (0.88–0.92) | <0.001 | <0.001 |
| L-LDL-C-pct | -0.026 | 0.003 | 0.97 (0.96–0.98) | <0.001 | <0.001 |
| L-VLDL-CE-pct | 0.098 | 0.010 | 1.10 (1.08–1.12) | <0.001 | <0.001 |
| L-VLDL-C-pct | 0.094 | 0.010 | 1.10 (1.08–1.12) | <0.001 | <0.001 |
| L-VLDL-FC | -0.093 | 0.010 | 0.91 (0.89–0.93) | <0.001 | <0.001 |
| M-VLDL-C | -0.101 | 0.011 | 0.90 (0.88–0.92) | <0.001 | <0.001 |
| L-VLDL-C | -0.092 | 0.010 | 0.91 (0.89–0.93) | <0.001 | <0.001 |
| HDL-P | -0.098 | 0.011 | 0.91 (0.89–0.93) | <0.001 | <0.001 |
| S-LDL-TG-pct | 0.085 | 0.009 | 1.09 (1.07–1.11) | <0.001 | <0.001 |
| L-LDL-P | -0.095 | 0.011 | 0.91 (0.89–0.93) | <0.001 | <0.001 |
| XL-VLDL-CE | -0.090 | 0.010 | 0.91 (0.90–0.93) | <0.001 | <0.001 |
| S-VLDL-FC | -0.092 | 0.010 | 0.91 (0.89–0.93) | <0.001 | <0.001 |
| MUFA-pct | 0.086 | 0.010 | 1.09 (1.07–1.11) | <0.001 | <0.001 |
| L-VLDL-P | -0.086 | 0.010 | 0.92 (0.90–0.94) | <0.001 | <0.001 |
| L-VLDL-CE | -0.087 | 0.010 | 0.92 (0.90–0.93) | <0.001 | <0.001 |
| VLDL-TG | -0.083 | 0.010 | 0.92 (0.90–0.94) | <0.001 | <0.001 |
| IDL-CE | -0.097 | 0.011 | 0.91 (0.89–0.93) | <0.001 | <0.001 |
| L-VLDL-PL | -0.083 | 0.010 | 0.92 (0.9–0.94) | <0.001 | <0.001 |
| VLDL-L | -0.081 | 0.010 | 0.92 (0.91–0.94) | <0.001 | <0.001 |
| XL-VLDL-TG-pct | -0.070 | 0.008 | 0.93 (0.92–0.95) | <0.001 | <0.001 |
| LA-pct | -0.080 | 0.010 | 0.92 (0.91–0.94) | <0.001 | <0.001 |
| S-VLDL-PL | -0.083 | 0.010 | 0.92 (0.90–0.94) | <0.001 | <0.001 |
| Citrate | 0.073 | 0.009 | 1.08 (1.06–1.09) | <0.001 | <0.001 |
| Remnant-C | -0.087 | 0.011 | 0.92 (0.90–0.94) | <0.001 | <0.001 |
| XL-HDL-PL-pct | 0.092 | 0.011 | 1.10 (1.07–1.12) | <0.001 | <0.001 |
| XL-VLDL-C | -0.080 | 0.010 | 0.92 (0.91–0.94) | <0.001 | <0.001 |
| XL-VLDL-L | -0.077 | 0.010 | 0.93 (0.91–0.94) | <0.001 | <0.001 |
| S-LDL-FC-pct | -0.057 | 0.007 | 0.94 (0.93–0.96) | <0.001 | <0.001 |
| M-VLDL-CE | -0.087 | 0.011 | 0.92 (0.90–0.94) | <0.001 | <0.001 |
| XL-HDL-TG-pct | -0.077 | 0.010 | 0.93 (0.91–0.94) | <0.001 | <0.001 |
| XL-VLDL-TG | -0.075 | 0.010 | 0.93 (0.91–0.95) | <0.001 | <0.001 |
| IDL-C | -0.086 | 0.011 | 0.92 (0.90–0.94) | <0.001 | <0.001 |
| XL-HDL-TG | 0.067 | 0.009 | 1.07 (1.05–1.09) | <0.001 | <0.001 |
| IDL-TG | 0.068 | 0.009 | 1.07 (1.05–1.09) | <0.001 | <0.001 |
| L-HDL-FC-pct | 0.079 | 0.010 | 1.08 (1.06–1.10) | <0.001 | <0.001 |
| Ile | -0.073 | 0.010 | 0.93 (0.91–0.95) | <0.001 | <0.001 |
| VLDL-FC | -0.074 | 0.010 | 0.93 (0.91–0.95) | <0.001 | <0.001 |
| Glucose | 0.063 | 0.009 | 1.07 (1.05–1.08) | <0.001 | <0.001 |
| XL-HDL-CE-pct | -0.040 | 0.006 | 0.96 (0.95–0.97) | <0.001 | <0.001 |
| XL-VLDL-P | -0.070 | 0.010 | 0.93 (0.91–0.95) | <0.001 | <0.001 |
| S-VLDL-L | -0.070 | 0.010 | 0.93 (0.92–0.95) | <0.001 | <0.001 |
| XS-VLDL-PL | 0.070 | 0.010 | 1.07 (1.05–1.09) | <0.001 | <0.001 |
| VLDL-C | -0.070 | 0.010 | 0.93 (0.91–0.95) | <0.001 | <0.001 |
| Ala | -0.062 | 0.009 | 0.94 (0.92–0.96) | <0.001 | <0.001 |
| XL-VLDL-FC | -0.066 | 0.010 | 0.94 (0.92–0.95) | <0.001 | <0.001 |
| VLDL-PL | -0.065 | 0.010 | 0.94 (0.92–0.95) | <0.001 | <0.001 |
| XL-VLDL-PL | -0.065 | 0.010 | 0.94 (0.92–0.96) | <0.001 | <0.001 |
| S-VLDL-C | -0.067 | 0.010 | 0.94 (0.92–0.95) | <0.001 | <0.001 |
| Gln | -0.058 | 0.009 | 0.94 (0.93–0.96) | <0.001 | <0.001 |
| Total-PL | -0.065 | 0.010 | 0.94 (0.92–0.96) | <0.001 | <0.001 |
| Total-TG | -0.060 | 0.009 | 0.94 (0.92–0.96) | <0.001 | <0.001 |
| Phosphatidylc | -0.065 | 0.010 | 0.94 (0.92–0.96) | <0.001 | <0.001 |
| S-VLDL-P | -0.059 | 0.010 | 0.94 (0.92–0.96) | <0.001 | <0.001 |
| ApoB/ApoA1 | -0.062 | 0.010 | 0.94 (0.92–0.96) | <0.001 | <0.001 |
| Total-FA | -0.056 | 0.009 | 0.95 (0.93–0.96) | <0.001 | <0.001 |
| M-HDL-CE-pct | -0.045 | 0.007 | 0.96 (0.94–0.97) | <0.001 | <0.001 |
| VLDL-CE | -0.062 | 0.010 | 0.94 (0.92–0.96) | <0.001 | <0.001 |
| M-VLDL-TG-pct | -0.061 | 0.010 | 0.94 (0.92–0.96) | <0.001 | <0.001 |
| IDL-P | -0.064 | 0.011 | 0.94 (0.92–0.96) | <0.001 | <0.001 |
| L-LDL-TG | 0.051 | 0.009 | 1.05 (1.03–1.07) | <0.001 | <0.001 |
| IDL-L | -0.063 | 0.011 | 0.94 (0.92–0.96) | <0.001 | <0.001 |
| Cholines | -0.059 | 0.010 | 0.94 (0.92–0.96) | <0.001 | <0.001 |
| XL-VLDL-C-pct | 0.057 | 0.010 | 1.06 (1.04–1.08) | <0.001 | <0.001 |
| Pyruvate | 0.045 | 0.008 | 1.05 (1.03–1.06) | <0.001 | <0.001 |
| S-VLDL-PL-pct | -0.057 | 0.010 | 0.94 (0.93–0.96) | <0.001 | <0.001 |
| Phosphoglyc | -0.055 | 0.010 | 0.95 (0.93–0.97) | <0.001 | <0.001 |
| M-HDL-C-pct | -0.041 | 0.007 | 0.96 (0.95–0.97) | <0.001 | <0.001 |
| S-VLDL-FC-pct | -0.054 | 0.010 | 0.95 (0.93–0.97) | <0.001 | <0.001 |
| M-HDL-PL-pct | 0.052 | 0.010 | 1.05 (1.03–1.07) | <0.001 | <0.001 |
| S-VLDL-TG | -0.050 | 0.009 | 0.95 (0.93–0.97) | <0.001 | <0.001 |
| L-VLDL-FC-pct | 0.055 | 0.010 | 1.06 (1.04–1.08) | <0.001 | <0.001 |
| VLDL-P | -0.051 | 0.010 | 0.95 (0.93–0.97) | <0.001 | <0.001 |
| S-VLDL-CE | -0.051 | 0.010 | 0.95 (0.93–0.97) | <0.001 | <0.001 |
| XS-VLDL-FC | 0.051 | 0.010 | 1.05 (1.03–1.07) | <0.001 | <0.001 |
| XS-VLDL-L | 0.049 | 0.010 | 1.05 (1.03–1.07) | <0.001 | <0.001 |
| TG/PG | -0.049 | 0.010 | 0.95 (0.93–0.97) | <0.001 | <0.001 |
| Omega-6-pct | -0.042 | 0.009 | 0.96 (0.94–0.98) | <0.001 | <0.001 |
| Phe | 0.039 | 0.008 | 1.04 (1.02–1.06) | <0.001 | <0.001 |
| IDL-FC | -0.052 | 0.011 | 0.95 (0.93–0.97) | <0.001 | <0.001 |
| M-VLDL-PL-pct | 0.047 | 0.010 | 1.05 (1.03–1.07) | <0.001 | <0.001 |
| L-HDL-PL-pct | -0.027 | 0.006 | 0.97 (0.96–0.99) | <0.001 | <0.001 |
| XS-VLDL-TG | 0.041 | 0.009 | 1.04 (1.02–1.06) | <0.001 | <0.001 |
| M-LDL-FC-pct | -0.035 | 0.008 | 0.97 (0.95–0.99) | <0.001 | <0.001 |
| HDL-FC | 0.048 | 0.011 | 1.05 (1.03–1.07) | <0.001 | <0.001 |
| S-HDL-TG-pct | 0.043 | 0.010 | 1.04 (1.02–1.07) | <0.001 | <0.001 |
| XXL-VLDL-FC-pct | 0.039 | 0.009 | 1.04 (1.02–1.06) | <0.001 | <0.001 |
| XS-VLDL-CE-pct | -0.041 | 0.010 | 0.96 (0.94–0.98) | <0.001 | <0.001 |
| XS-VLDL-P | 0.041 | 0.010 | 1.04 (1.02–1.06) | <0.001 | <0.001 |
| M-HDL-CE | -0.043 | 0.011 | 0.96 (0.94–0.98) | <0.001 | <0.001 |
| M-VLDL-FC-pct | 0.040 | 0.010 | 1.04 (1.02–1.06) | <0.001 | <0.001 |
| XXL-VLDL-PL-pct | 0.044 | 0.012 | 1.05 (1.02–1.07) | <0.001 | <0.001 |
| XL-VLDL-CE-pct | 0.037 | 0.010 | 1.04 (1.02–1.06) | <0.001 | <0.001 |
| M-HDL-C | -0.039 | 0.011 | 0.96 (0.94–0.98) | <0.001 | <0.001 |
| XXL-VLDL-TG | -0.035 | 0.009 | 0.97 (0.95–0.98) | <0.001 | <0.001 |
| L-LDL-FC-pct | -0.029 | 0.008 | 0.97 (0.96–0.99) | <0.001 | <0.001 |
| S-VLDL-CE-pct | 0.035 | 0.010 | 1.04 (1.02–1.06) | <0.001 | <0.001 |
| LDL-TG | 0.032 | 0.009 | 1.03 (1.01–1.05) | <0.001 | <0.001 |
| XS-VLDL-C-pct | -0.035 | 0.010 | 0.97 (0.95–0.98) | <0.001 | 0.001 |
| XXL-VLDL-L | -0.033 | 0.009 | 0.97 (0.95–0.99) | <0.001 | 0.001 |
| M-HDL-L | -0.035 | 0.010 | 0.97 (0.95–0.99) | 0.001 | 0.001 |
| Lactate | 0.030 | 0.009 | 1.03 (1.01–1.05) | 0.001 | 0.001 |
| L-LDL-PL-pct | 0.027 | 0.009 | 1.03 (1.01–1.05) | 0.001 | 0.002 |
| S-HDL-TG | -0.031 | 0.010 | 0.97 (0.95–0.99) | 0.001 | 0.002 |
| Tyr | 0.029 | 0.009 | 1.03 (1.01–1.05) | 0.001 | 0.002 |
| LDL-size | 0.030 | 0.009 | 1.03 (1.01–1.05) | 0.002 | 0.002 |
| XS-VLDL-C | 0.034 | 0.011 | 1.03 (1.01–1.06) | 0.002 | 0.002 |
| XXL-VLDL-CE | -0.030 | 0.010 | 0.97 (0.95–0.99) | 0.002 | 0.003 |
| IDL-PL | -0.034 | 0.011 | 0.97 (0.95–0.99) | 0.002 | 0.003 |
| XXL-VLDL-C | -0.028 | 0.010 | 0.97 (0.95–0.99) | 0.003 | 0.003 |
| XXL-VLDL-P | -0.028 | 0.009 | 0.97 (0.96–0.99) | 0.003 | 0.004 |
| ApoA1 | -0.031 | 0.011 | 0.97 (0.95–0.99) | 0.004 | 0.004 |
| M-HDL-TG-pct | 0.029 | 0.010 | 1.03 (1.01–1.05) | 0.004 | 0.004 |
| M-VLDL-C-pct | 0.028 | 0.010 | 1.03 (1.01–1.05) | 0.005 | 0.005 |
| XXL-VLDL-FC | -0.026 | 0.009 | 0.97 (0.96–0.99) | 0.006 | 0.007 |
| XXL-VLDL-PL | -0.025 | 0.009 | 0.98 (0.96–0.99) | 0.008 | 0.009 |
| M-HDL-PL | -0.027 | 0.010 | 0.97 (0.95–0.99) | 0.009 | 0.011 |
| M-HDL-P | -0.027 | 0.011 | 0.97 (0.95–0.99) | 0.011 | 0.012 |
| SFA | -0.023 | 0.009 | 0.98 (0.96–0.99) | 0.012 | 0.014 |
| L-HDL-TG-pct | -0.024 | 0.010 | 0.98 (0.96–0.99) | 0.014 | 0.016 |
| L-HDL-C-pct | 0.022 | 0.009 | 1.02 (1.01–1.04) | 0.015 | 0.016 |
| XS-VLDL-CE | 0.025 | 0.011 | 1.03 (1.01–1.05) | 0.026 | 0.028 |
| HDL-TG | 0.020 | 0.009 | 1.02 (1.01–1.04) | 0.030 | 0.033 |
| XXL-VLDL-C-pct | 0.018 | 0.010 | 1.02 (1.00–1.04) | 0.057 | 0.063 |
| M-VLDL-CE-pct | 0.015 | 0.009 | 1.01 (1.00–1.03) | 0.114 | 0.125 |
| S-LDL-TG | -0.014 | 0.009 | 0.99 (0.97–1.00) | 0.122 | 0.134 |
| HDL-PL | 0.016 | 0.011 | 1.02 (0.99–1.04) | 0.136 | 0.148 |
| M-HDL-TG | 0.012 | 0.009 | 1.01 (0.99–1.03) | 0.189 | 0.206 |
| M-HDL-FC | -0.013 | 0.011 | 0.99 (0.97–1.01) | 0.218 | 0.237 |
| M-LDL-TG | 0.011 | 0.009 | 1.01 (0.99–1.03) | 0.225 | 0.243 |
| XS-VLDL-FC-pct | 0.011 | 0.009 | 1.01 (0.99–1.03) | 0.249 | 0.268 |
| S-VLDL-TG-pct | 0.011 | 0.011 | 1.01 (0.99–1.03) | 0.277 | 0.296 |
| Gly | 0.011 | 0.011 | 1.01 (0.99–1.03) | 0.279 | 0.296 |
| L-HDL-CE-pct | 0.009 | 0.008 | 1.01 (0.99–1.03) | 0.279 | 0.296 |
| XXL-VLDL-TG-pct | -0.009 | 0.009 | 0.99 (0.97–1.01) | 0.331 | 0.349 |
| HDL-L | 0.010 | 0.011 | 1.01 (0.99–1.03) | 0.361 | 0.380 |
| L-VLDL-PL-pct | -0.009 | 0.010 | 0.99 (0.97–1.01) | 0.378 | 0.395 |
| MUFA | -0.008 | 0.009 | 0.99 (0.97–1.01) | 0.405 | 0.422 |
| XL-VLDL-PL-pct | 0.008 | 0.010 | 1.01 (0.99–1.03) | 0.424 | 0.440 |
| Sphingomyelins | -0.008 | 0.011 | 0.99 (0.97–1.01) | 0.469 | 0.485 |
| M-LDL-PL-pct | 0.006 | 0.009 | 1.01 (0.99–1.02) | 0.494 | 0.509 |
| IDL-FC-pct | 0.005 | 0.009 | 1.01 (0.99–1.02) | 0.554 | 0.568 |
| HDL-CE | -0.007 | 0.011 | 0.99 (0.97–1.02) | 0.557 | 0.569 |
| XXL-VLDL-CE-pct | 0.004 | 0.010 | 1.00 (0.99–1.02) | 0.657 | 0.668 |
| HDL-C | 0.004 | 0.011 | 1.00 (0.98–1.03) | 0.749 | 0.758 |
| XS-VLDL-TG-pct | 0.003 | 0.011 | 1.00 (0.98–1.02) | 0.788 | 0.795 |
| M-HDL-FC-pct | -0.003 | 0.011 | 1.00 (0.98–1.02) | 0.792 | 0.795 |
| S-VLDL-C-pct | 0.000 | 0.010 | 1.00 (0.98–1.02) | 0.974 | 0.974 |

Abbreviations: CI, confidence interval; FDR, false discovery rate;^[3]^ HR, hazard ratio; SE, standard error.

Notes: The names of the metabolic biomarkers are indicated by representative abbreviations, and the full names of which are detailed in **Table S10** (Supporting Information).

**a)** All results were from the multivariable Cox models adjusted for age, sex, education level, smoking status, physical activity, alcohol consumption, body mass index, treated dyslipidemia, hypertension, diabetes, cardiovascular disease, and cancer.

**b)** Schoenfeld residuals were used to assess the proportional hazards assumption in the Cox models.^[1-2, 4]^

# Table S3. Longitudinal associations of 224 selected metabolites with 10-year all-cause mortality in the total study population of the ESTHER study.

| **Metabolic biomarkers** | **Estimate** | **SE** | **HR (95% CI) ^a^** | ***P*-value** | **FDR-adjusted *P*-value** |
| --- | --- | --- | --- | --- | --- |
| M-LDL-C-pct | -0.126 | 0.016 | 0.88 (0.85–0.91) | <0.001 | <0.001 |
| S-LDL-C-pct | -0.146 | 0.019 | 0.86 (0.83–0.90) | <0.001 | <0.001 |
| Albumin | -0.197 | 0.034 | 0.82 (0.77–0.88) | <0.001 | <0.001 |
| HDL-size | 0.188 | 0.034 | 1.21 (1.13–1.29) | <0.001 | <0.001 |
| M-LDL-TG-pct | 0.182 | 0.035 | 1.20 (1.12–1.29) | <0.001 | <0.001 |
| XL-HDL-PL | 0.173 | 0.034 | 1.19 (1.11–1.27) | <0.001 | <0.001 |
| XL-HDL-L | 0.164 | 0.034 | 1.18 (1.10–1.26) | <0.001 | <0.001 |
| M-LDL-CE-pct | -0.112 | 0.024 | 0.89 (0.85–0.94) | <0.001 | <0.001 |
| XL-HDL-P | 0.154 | 0.033 | 1.17 (1.09–1.24) | <0.001 | <0.001 |
| S-HDL-CE-pct | -0.130 | 0.029 | 0.88 (0.83–0.93) | <0.001 | <0.001 |
| L-LDL-TG-pct | 0.139 | 0.031 | 1.15 (1.08–1.22) | <0.001 | <0.001 |
| L-LDL-CE-pct | -0.102 | 0.024 | 0.90 (0.86–0.95) | <0.001 | <0.001 |
| XL-HDL-FC | 0.139 | 0.033 | 1.15 (1.08–1.23) | <0.001 | <0.001 |
| S-HDL-C-pct | -0.126 | 0.030 | 0.88 (0.83–0.94) | <0.001 | <0.001 |
| XL-HDL-C | 0.142 | 0.034 | 1.15 (1.08–1.23) | <0.001 | 0.001 |
| S-HDL-PL-pct | 0.130 | 0.033 | 1.14 (1.07–1.21) | <0.001 | 0.001 |
| XL-HDL-CE | 0.137 | 0.035 | 1.15 (1.07–1.23) | <0.001 | 0.001 |
| S-HDL-CE | -0.130 | 0.033 | 0.88 (0.82–0.94) | <0.001 | 0.001 |
| L-LDL-C-pct | -0.090 | 0.023 | 0.91 (0.87–0.96) | <0.001 | 0.002 |
| L-HDL-FC | 0.138 | 0.036 | 1.15 (1.07–1.23) | <0.001 | 0.002 |
| SFA-pct | 0.119 | 0.031 | 1.13 (1.06–1.20) | <0.001 | 0.002 |
| S-LDL-FC-pct | -0.085 | 0.023 | 0.92 (0.88–0.96) | <0.001 | 0.002 |
| bOHbutyrate | 0.099 | 0.027 | 1.10 (1.05–1.16) | <0.001 | 0.002 |
| L-HDL-P | 0.133 | 0.036 | 1.14 (1.06–1.23) | <0.001 | 0.002 |
| M-LDL-FC | -0.121 | 0.033 | 0.89 (0.83–0.94) | <0.001 | 0.002 |
| PUFA-pct | -0.108 | 0.030 | 0.90 (0.85–0.95) | <0.001 | 0.002 |
| S-LDL-TG-pct | 0.136 | 0.038 | 1.15 (1.06–1.23) | <0.001 | 0.003 |
| L-HDL-L | 0.129 | 0.037 | 1.14 (1.06–1.22) | <0.001 | 0.004 |
| LDL-C | -0.113 | 0.032 | 0.89 (0.84–0.95) | <0.001 | 0.004 |
| M-LDL-C | -0.114 | 0.033 | 0.89 (0.84–0.95) | <0.001 | 0.004 |
| S-HDL-C | -0.118 | 0.034 | 0.89 (0.83–0.95) | 0.001 | 0.004 |
| GlycA | 0.114 | 0.033 | 1.12 (1.05–1.20) | 0.001 | 0.004 |
| L-HDL-PL | 0.127 | 0.037 | 1.14 (1.06–1.22) | 0.001 | 0.004 |
| Clinical-LDL-C | -0.111 | 0.032 | 0.90 (0.84–0.95) | 0.001 | 0.004 |
| IDL-TG-pct | 0.112 | 0.033 | 1.12 (1.05–1.19) | 0.001 | 0.004 |
| LDL-CE | -0.111 | 0.032 | 0.90 (0.84–0.95) | 0.001 | 0.004 |
| LDL-FC | -0.112 | 0.033 | 0.89 (0.84–0.95) | 0.001 | 0.004 |
| S-LDL-FC | -0.109 | 0.032 | 0.90 (0.84–0.96) | 0.001 | 0.005 |
| IDL-CE-pct | -0.100 | 0.030 | 0.91 (0.85–0.96) | 0.001 | 0.005 |
| S-HDL-P | -0.114 | 0.034 | 0.89 (0.83–0.95) | 0.001 | 0.005 |
| M-LDL-PL | -0.109 | 0.033 | 0.90 (0.84–0.96) | 0.001 | 0.005 |
| L-LDL-CE | -0.107 | 0.033 | 0.90 (0.84–0.96) | 0.001 | 0.006 |
| L-LDL-C | -0.106 | 0.033 | 0.90 (0.84–0.96) | 0.001 | 0.006 |
| LA-pct | -0.076 | 0.023 | 0.93 (0.89–0.97) | 0.001 | 0.006 |
| Omega-6-pct | -0.098 | 0.030 | 0.91 (0.85–0.96) | 0.001 | 0.006 |
| M-LDL-L | -0.106 | 0.033 | 0.90 (0.84–0.96) | 0.001 | 0.006 |
| L-HDL-TG | 0.098 | 0.030 | 1.10 (1.04–1.17) | 0.001 | 0.006 |
| L-HDL-C | 0.119 | 0.037 | 1.13 (1.05–1.21) | 0.001 | 0.006 |
| PUFA/MUFA | -0.103 | 0.032 | 0.90 (0.85–0.96) | 0.002 | 0.007 |
| LDL-L | -0.102 | 0.032 | 0.90 (0.85–0.96) | 0.002 | 0.007 |
| M-LDL-CE | -0.104 | 0.033 | 0.90 (0.85–0.96) | 0.002 | 0.007 |
| LDL-PL | -0.103 | 0.033 | 0.90 (0.85–0.96) | 0.002 | 0.007 |
| L-LDL-FC | -0.106 | 0.034 | 0.90 (0.84–0.96) | 0.002 | 0.007 |
| IDL-C-pct | -0.092 | 0.030 | 0.91 (0.86–0.97) | 0.002 | 0.007 |
| M-LDL-FC-pct | -0.084 | 0.027 | 0.92 (0.87–0.97) | 0.002 | 0.009 |
| Unsaturation | -0.096 | 0.032 | 0.91 (0.85–0.97) | 0.002 | 0.009 |
| L-LDL-PL | -0.102 | 0.033 | 0.90 (0.85–0.96) | 0.002 | 0.009 |
| L-HDL-CE | 0.113 | 0.037 | 1.12 (1.04–1.20) | 0.002 | 0.009 |
| S-LDL-C | -0.097 | 0.033 | 0.91 (0.85–0.97) | 0.003 | 0.012 |
| L-LDL-L | -0.096 | 0.033 | 0.91 (0.85–0.97) | 0.003 | 0.012 |
| Acetate | 0.062 | 0.022 | 1.06 (1.02–1.11) | 0.006 | 0.021 |
| XS-VLDL-PL-pct | 0.091 | 0.035 | 1.10 (1.02–1.17) | 0.010 | 0.034 |
| S-LDL-L | -0.083 | 0.033 | 0.92 (0.86–0.98) | 0.011 | 0.039 |
| S-LDL-CE | -0.084 | 0.033 | 0.92 (0.86–0.98) | 0.011 | 0.039 |
| non-HDL-C | -0.082 | 0.032 | 0.92 (0.86–0.98) | 0.011 | 0.039 |
| XL-HDL-TG | 0.078 | 0.031 | 1.08 (1.02–1.15) | 0.012 | 0.042 |
| MUFA-pct | 0.082 | 0.033 | 1.09 (1.02–1.16) | 0.013 | 0.042 |
| L-LDL-P | -0.083 | 0.034 | 0.92 (0.86–0.98) | 0.013 | 0.042 |
| LDL-P | -0.079 | 0.033 | 0.92 (0.87–0.99) | 0.017 | 0.054 |
| XL-HDL-C-pct | -0.045 | 0.019 | 0.96 (0.92–0.99) | 0.017 | 0.055 |
| S-VLDL-FC-pct | -0.077 | 0.032 | 0.93 (0.87–0.99) | 0.018 | 0.056 |
| His | -0.082 | 0.035 | 0.92 (0.86–0.99) | 0.020 | 0.062 |
| S-HDL-FC-pct | 0.070 | 0.030 | 1.07 (1.01–1.14) | 0.020 | 0.062 |
| S-LDL-PL | -0.075 | 0.033 | 0.93 (0.87–0.99) | 0.021 | 0.065 |
| Total-CE | -0.074 | 0.033 | 0.93 (0.87–0.99) | 0.026 | 0.077 |
| S-HDL-L | -0.075 | 0.034 | 0.93 (0.87–0.99) | 0.026 | 0.078 |
| XL-VLDL-CE-pct | -0.077 | 0.035 | 0.93 (0.86–0.99) | 0.027 | 0.080 |
| S-VLDL-PL-pct | -0.073 | 0.033 | 0.93 (0.87–0.99) | 0.029 | 0.083 |
| Acetoacetate | 0.045 | 0.021 | 1.05 (1.00–1.09) | 0.030 | 0.084 |
| HDL-FC | 0.079 | 0.037 | 1.08 (1.01–1.16) | 0.031 | 0.088 |
| ApoB/ApoA1 | -0.072 | 0.034 | 0.93 (0.87–0.99) | 0.033 | 0.090 |
| ApoB | -0.070 | 0.033 | 0.93 (0.87–0.99) | 0.033 | 0.090 |
| S-LDL-CE-pct | -0.069 | 0.033 | 0.93 (0.88–0.99) | 0.036 | 0.096 |
| IDL-CE | -0.070 | 0.034 | 0.93 (0.87–0.99) | 0.036 | 0.096 |
| M-LDL-P | -0.069 | 0.033 | 0.93 (0.88–0.99) | 0.038 | 0.099 |
| Total-C | -0.068 | 0.033 | 0.93 (0.88–0.99) | 0.039 | 0.102 |
| XL-HDL-FC-pct | -0.051 | 0.025 | 0.95 (0.90–0.99) | 0.041 | 0.105 |
| HDL-TG | 0.064 | 0.031 | 1.07 (1.01–1.13) | 0.042 | 0.106 |
| IDL-TG | 0.062 | 0.031 | 1.06 (1.01–1.13) | 0.044 | 0.112 |
| XL-HDL-TG-pct | 0.063 | 0.031 | 1.06 (1.01–1.13) | 0.045 | 0.113 |
| XS-VLDL-CE-pct | -0.063 | 0.032 | 0.94 (0.88–0.99) | 0.048 | 0.119 |
| IDL-C | -0.065 | 0.033 | 0.94 (0.88–0.99) | 0.049 | 0.120 |
| XS-VLDL-C-pct | -0.061 | 0.032 | 0.94 (0.88–1.00) | 0.053 | 0.129 |
| LA | -0.062 | 0.032 | 0.94 (0.88–1.00) | 0.054 | 0.129 |
| LDL-TG | 0.059 | 0.031 | 1.06 (1.00–1.13) | 0.056 | 0.131 |
| XS-VLDL-TG | 0.059 | 0.031 | 1.06 (1.00–1.13) | 0.060 | 0.141 |
| IDL-P | -0.063 | 0.034 | 0.94 (0.88–1.00) | 0.062 | 0.143 |
| L-LDL-TG | 0.056 | 0.030 | 1.06 (1.00–1.12) | 0.065 | 0.148 |
| L-LDL-FC-pct | -0.047 | 0.026 | 0.95 (0.91–1.00) | 0.068 | 0.155 |
| S-HDL-TG-pct | 0.063 | 0.035 | 1.07 (0.99–1.14) | 0.070 | 0.157 |
| Val | -0.064 | 0.035 | 0.94 (0.88–1.01) | 0.071 | 0.157 |
| IDL-FC | -0.060 | 0.034 | 0.94 (0.88–1.01) | 0.072 | 0.157 |
| Omega-3-pct | -0.057 | 0.032 | 0.94 (0.89–1.01) | 0.077 | 0.167 |
| M-VLDL-CE | -0.057 | 0.033 | 0.94 (0.89–1.01) | 0.082 | 0.176 |
| Citrate | 0.055 | 0.032 | 1.06 (0.99–1.13) | 0.083 | 0.176 |
| XXL-VLDL-P | 0.052 | 0.031 | 1.05 (0.99–1.12) | 0.089 | 0.188 |
| XXL-VLDL-PL | 0.053 | 0.031 | 1.05 (0.99–1.12) | 0.092 | 0.192 |
| Phe | 0.053 | 0.032 | 1.05 (0.99–1.12) | 0.093 | 0.193 |
| Omega-6 | -0.054 | 0.033 | 0.95 (0.89–1.01) | 0.104 | 0.213 |
| Leu | -0.056 | 0.035 | 0.95 (0.88–1.01) | 0.105 | 0.213 |
| M-VLDL-C | -0.053 | 0.033 | 0.95 (0.89–1.01) | 0.106 | 0.213 |
| XXL-VLDL-C | 0.051 | 0.032 | 1.05 (0.99–1.12) | 0.109 | 0.217 |
| Total-FC | -0.052 | 0.033 | 0.95 (0.89–1.01) | 0.110 | 0.217 |
| XXL-VLDL-L | 0.051 | 0.032 | 1.05 (0.99–1.12) | 0.110 | 0.217 |
| M-VLDL-L | -0.053 | 0.033 | 0.95 (0.89–1.01) | 0.112 | 0.218 |
| PUFA | -0.052 | 0.033 | 0.95 (0.89–1.01) | 0.113 | 0.218 |
| IDL-PL | -0.052 | 0.033 | 0.95 (0.89–1.01) | 0.115 | 0.220 |
| S-LDL-P | -0.051 | 0.033 | 0.95 (0.89–1.01) | 0.119 | 0.226 |
| XXL-VLDL-TG | 0.048 | 0.031 | 1.05 (0.99–1.12) | 0.120 | 0.226 |
| XXL-VLDL-CE | 0.049 | 0.032 | 1.05 (0.99–1.12) | 0.125 | 0.233 |
| Total-BCAA | -0.054 | 0.035 | 0.95 (0.88–1.02) | 0.126 | 0.233 |
| Acetone | 0.040 | 0.026 | 1.04 (0.99–1.10) | 0.128 | 0.235 |
| XXL-VLDL-FC | 0.048 | 0.032 | 1.05 (0.99–1.12) | 0.130 | 0.236 |
| L-HDL-FC-pct | 0.055 | 0.036 | 1.06 (0.98–1.13) | 0.133 | 0.239 |
| Gln | -0.049 | 0.033 | 0.95 (0.89–1.02) | 0.133 | 0.239 |
| IDL-L | -0.049 | 0.033 | 0.95 (0.89–1.02) | 0.138 | 0.245 |
| XL-HDL-CE-pct | -0.030 | 0.021 | 0.97 (0.93–1.01) | 0.155 | 0.274 |
| S-HDL-PL | -0.048 | 0.034 | 0.95 (0.89–1.02) | 0.158 | 0.276 |
| M-HDL-TG-pct | 0.048 | 0.035 | 1.05 (0.98–1.12) | 0.163 | 0.283 |
| Remnant-C | -0.044 | 0.033 | 0.96 (0.90–1.02) | 0.175 | 0.297 |
| SFA | 0.043 | 0.032 | 1.04 (0.98–1.11) | 0.175 | 0.297 |
| M-VLDL-PL | -0.045 | 0.033 | 0.96 (0.90–1.02) | 0.176 | 0.297 |
| VLDL-size | -0.045 | 0.033 | 0.96 (0.90–1.02) | 0.176 | 0.297 |
| S-VLDL-FC | -0.044 | 0.033 | 0.96 (0.90–1.02) | 0.188 | 0.315 |
| L-VLDL-FC-pct | -0.043 | 0.033 | 0.96 (0.90–1.02) | 0.194 | 0.322 |
| L-VLDL-TG-pct | -0.035 | 0.027 | 0.97 (0.92–1.02) | 0.201 | 0.331 |
| M-VLDL-FC | -0.042 | 0.033 | 0.96 (0.90–1.02) | 0.206 | 0.336 |
| M-VLDL-P | -0.042 | 0.033 | 0.96 (0.90–1.02) | 0.207 | 0.337 |
| M-HDL-C-pct | -0.037 | 0.030 | 0.96 (0.91–1.02) | 0.220 | 0.354 |
| S-HDL-FC | -0.042 | 0.034 | 0.96 (0.90–1.03) | 0.221 | 0.354 |
| XL-VLDL-TG-pct | -0.041 | 0.034 | 0.96 (0.90–1.03) | 0.227 | 0.361 |
| S-VLDL-CE-pct | -0.036 | 0.032 | 0.96 (0.91–1.03) | 0.254 | 0.401 |
| XL-HDL-PL-pct | 0.040 | 0.036 | 1.04 (0.97–1.12) | 0.257 | 0.402 |
| XL-VLDL-C-pct | -0.040 | 0.036 | 0.96 (0.90–1.03) | 0.264 | 0.409 |
| ApoA1 | 0.040 | 0.036 | 1.04 (0.97–1.12) | 0.265 | 0.409 |
| Total-P | -0.040 | 0.036 | 0.96 (0.89–1.03) | 0.272 | 0.418 |
| M-VLDL-TG | -0.036 | 0.033 | 0.96 (0.90–1.03) | 0.279 | 0.426 |
| Tyr | 0.036 | 0.034 | 1.04 (0.97–1.11) | 0.287 | 0.434 |
| Lactate | 0.034 | 0.032 | 1.03 (0.97–1.10) | 0.293 | 0.440 |
| Total-L | -0.033 | 0.033 | 0.97 (0.91–1.03) | 0.309 | 0.462 |
| L-VLDL-C-pct | -0.029 | 0.030 | 0.97 (0.92–1.03) | 0.346 | 0.512 |
| XS-VLDL-PL | 0.030 | 0.032 | 1.03 (0.97–1.10) | 0.348 | 0.512 |
| S-VLDL-PL | -0.031 | 0.033 | 0.97 (0.91–1.03) | 0.354 | 0.518 |
| XL-VLDL-CE | -0.031 | 0.034 | 0.97 (0.91–1.04) | 0.356 | 0.518 |
| M-HDL-CE-pct | -0.024 | 0.026 | 0.98 (0.93–1.03) | 0.365 | 0.527 |
| DHA-pct | -0.028 | 0.031 | 0.97 (0.91–1.03) | 0.368 | 0.528 |
| S-VLDL-C | -0.030 | 0.033 | 0.97 (0.91–1.04) | 0.371 | 0.529 |
| S-HDL-TG | 0.029 | 0.033 | 1.03 (0.97–1.10) | 0.377 | 0.534 |
| M-HDL-P | 0.032 | 0.036 | 1.03 (0.96–1.11) | 0.379 | 0.534 |
| L-VLDL-CE-pct | -0.028 | 0.033 | 0.97 (0.91–1.04) | 0.395 | 0.554 |
| Omega-3 | -0.027 | 0.033 | 0.97 (0.91–1.04) | 0.412 | 0.573 |
| L-VLDL-TG | -0.027 | 0.033 | 0.97 (0.91–1.04) | 0.417 | 0.573 |
| L-LDL-PL-pct | -0.026 | 0.032 | 0.97 (0.91–1.04) | 0.417 | 0.573 |
| XS-VLDL-L | 0.025 | 0.032 | 1.03 (0.96–1.09) | 0.428 | 0.585 |
| LDL-size | -0.025 | 0.032 | 0.97 (0.92–1.04) | 0.431 | 0.585 |
| M-VLDL-C-pct | -0.025 | 0.032 | 0.98 (0.92–1.04) | 0.433 | 0.585 |
| IDL-PL-pct | 0.025 | 0.033 | 1.03 (0.96–1.09) | 0.453 | 0.607 |
| HDL-P | -0.026 | 0.036 | 0.97 (0.91–1.05) | 0.470 | 0.627 |
| S-LDL-PL-pct | 0.025 | 0.035 | 1.03 (0.96–1.10) | 0.473 | 0.627 |
| L-VLDL-L | -0.024 | 0.033 | 0.98 (0.91–1.04) | 0.479 | 0.630 |
| Glucose | 0.023 | 0.033 | 1.02 (0.96–1.09) | 0.486 | 0.637 |
| VLDL-CE | -0.023 | 0.033 | 0.98 (0.92–1.04) | 0.494 | 0.643 |
| M-HDL-PL | 0.024 | 0.035 | 1.02 (0.96–1.10) | 0.506 | 0.655 |
| Total-TG | 0.021 | 0.033 | 1.02 (0.96–1.09) | 0.529 | 0.681 |
| S-VLDL-CE | -0.021 | 0.033 | 0.98 (0.92–1.05) | 0.533 | 0.682 |
| M-HDL-L | 0.021 | 0.036 | 1.02 (0.95–1.10) | 0.550 | 0.700 |
| L-VLDL-CE | -0.020 | 0.034 | 0.98 (0.92–1.05) | 0.554 | 0.701 |
| VLDL-C | -0.018 | 0.033 | 0.98 (0.92–1.05) | 0.581 | 0.731 |
| S-VLDL-TG | 0.018 | 0.033 | 1.02 (0.95–1.09) | 0.590 | 0.732 |
| XXL-VLDL-PL-pct | 0.019 | 0.035 | 1.02 (0.95–1.09) | 0.590 | 0.732 |
| Omega-6/Omega-3 | 0.017 | 0.032 | 1.02 (0.95–1.08) | 0.591 | 0.732 |
| Phosphoglyc | 0.018 | 0.034 | 1.02 (0.95–1.09) | 0.607 | 0.747 |
| L-VLDL-C | -0.017 | 0.034 | 0.98 (0.92–1.05) | 0.611 | 0.748 |
| XL-VLDL-PL | 0.017 | 0.033 | 1.02 (0.95–1.09) | 0.614 | 0.748 |
| TG/PG | 0.017 | 0.034 | 1.02 (0.95–1.09) | 0.622 | 0.753 |
| M-VLDL-PL-pct | 0.015 | 0.032 | 1.01 (0.95–1.08) | 0.642 | 0.765 |
| XS-VLDL-CE | -0.015 | 0.033 | 0.99 (0.92–1.05) | 0.643 | 0.765 |
| Phosphatidylc | 0.016 | 0.035 | 1.02 (0.95–1.09) | 0.647 | 0.765 |
| Total-FA | 0.015 | 0.032 | 1.01 (0.95–1.08) | 0.652 | 0.765 |
| XS-VLDL-FC | 0.014 | 0.032 | 1.01 (0.95–1.08) | 0.654 | 0.765 |
| L-HDL-TG-pct | 0.015 | 0.034 | 1.02 (0.95–1.09) | 0.658 | 0.765 |
| XS-VLDL-P | 0.014 | 0.032 | 1.01 (0.95–1.08) | 0.659 | 0.765 |
| XL-VLDL-FC | 0.015 | 0.033 | 1.01 (0.95–1.08) | 0.659 | 0.765 |
| Pyruvate | 0.014 | 0.032 | 1.01 (0.95–1.08) | 0.667 | 0.770 |
| XL-VLDL-P | 0.013 | 0.033 | 1.01 (0.95–1.08) | 0.691 | 0.791 |
| L-VLDL-FC | -0.013 | 0.034 | 0.99 (0.92–1.05) | 0.692 | 0.791 |
| DHA | -0.012 | 0.032 | 0.99 (0.93–1.05) | 0.703 | 0.800 |
| S-VLDL-L | -0.012 | 0.033 | 0.99 (0.93–1.05) | 0.718 | 0.812 |
| Ala | -0.011 | 0.033 | 0.99 (0.93–1.05) | 0.728 | 0.820 |
| M-HDL-C | 0.011 | 0.036 | 1.01 (0.94–1.09) | 0.770 | 0.861 |
| VLDL-FC | -0.010 | 0.033 | 0.99 (0.93–1.06) | 0.772 | 0.861 |
| Total-PL | -0.009 | 0.034 | 0.99 (0.93–1.06) | 0.781 | 0.866 |
| XL-VLDL-C | -0.009 | 0.034 | 0.99 (0.93–1.06) | 0.800 | 0.883 |
| VLDL-L | -0.008 | 0.033 | 0.99 (0.93–1.06) | 0.807 | 0.887 |
| XL-VLDL-TG | 0.007 | 0.033 | 1.01 (0.94–1.07) | 0.837 | 0.913 |
| M-HDL-CE | 0.007 | 0.036 | 1.01 (0.94–1.08) | 0.840 | 0.913 |
| L-HDL-PL-pct | -0.004 | 0.028 | 1.00 (0.94–1.05) | 0.879 | 0.951 |
| Creatinine | 0.005 | 0.035 | 1.01 (0.94–1.08) | 0.888 | 0.956 |
| M-VLDL-TG-pct | -0.004 | 0.033 | 1.00 (0.93–1.06) | 0.893 | 0.957 |
| XS-VLDL-C | -0.004 | 0.032 | 1.00 (0.94–1.06) | 0.907 | 0.957 |
| L-HDL-C-pct | 0.003 | 0.030 | 1.00 (0.95–1.06) | 0.911 | 0.957 |
| VLDL-P | -0.004 | 0.033 | 1.00 (0.93–1.06) | 0.912 | 0.957 |
| XL-VLDL-L | 0.004 | 0.033 | 1.00 (0.94–1.07) | 0.914 | 0.957 |
| Cholines | 0.004 | 0.035 | 1.00 (0.94–1.07) | 0.915 | 0.957 |
| L-VLDL-PL | -0.003 | 0.033 | 1.00 (0.93–1.06) | 0.922 | 0.960 |
| M-VLDL-FC-pct | -0.003 | 0.033 | 1.00 (0.93–1.06) | 0.926 | 0.960 |
| S-VLDL-P | -0.003 | 0.033 | 1.00 (0.93–1.06) | 0.937 | 0.968 |
| XL-VLDL-FC-pct | -0.002 | 0.036 | 1.00 (0.93–1.07) | 0.950 | 0.974 |
| L-VLDL-P | -0.002 | 0.033 | 1.00 (0.94–1.06) | 0.953 | 0.974 |
| XXL-VLDL-FC-pct | -0.002 | 0.034 | 1.00 (0.93–1.07) | 0.958 | 0.974 |
| VLDL-PL | -0.002 | 0.033 | 1.00 (0.94–1.07) | 0.963 | 0.974 |
| Ile | -0.001 | 0.035 | 1.00 (0.93–1.07) | 0.966 | 0.974 |
| M-HDL-PL-pct | 0.001 | 0.031 | 1.00 (0.94–1.06) | 0.969 | 0.974 |
| VLDL-TG | 0.001 | 0.033 | 1.00 (0.94–1.07) | 0.988 | 0.988 |

Abbreviations: CI, confidence interval; FDR, false discovery rate [3]; HR, hazard ratio; SE, standard error.

Notes: The names of the metabolic biomarkers are indicated by representative abbreviations, and the full names of which are detailed in **Table S10** (Supporting Information).

**a)** All results were from the multivariable Cox models adjusted for age, sex, education level, smoking status, physical activity, alcohol consumption, body mass index, treated dyslipidemia, hypertension, diabetes, cardiovascular disease, and cancer.

**b)** Schoenfeld residuals were used to assess the proportional hazards assumption in the Cox models.^[1-2, 4]^

# Table S4. Summary of potential KEGG pathways associated with 68 validated metabolic biomarkers identified from WGCNA-derived metabolite clusters.

| **Metabolite clusters** | **Class of pathways ^a^** | **Subclass of pathways** | **Pathways in KEGG database** |
| --- | --- | --- | --- |
| Cluster 1 |  |  |  |
|  | Human Diseases | Cardiovascular disease | Lipid and atherosclerosis ([map05417](https://www.kegg.jp/entry/map05417)) |
|  | Organismal Systems | Digestive system | Cholesterol metabolism ([map04979](https://www.kegg.jp/entry/map04979)) |
|  | Drug Development | Chronology: Other drugs | Antidyslipidemic agents ([map07052](https://www.kegg.jp/entry/map07052)) |
|  | Organismal Systems | Endocrine system | Ovarian steroidogenesis ([map04913](https://www.kegg.jp/entry/map04913)) |
|  | Organismal Systems | Endocrine system | Aldosterone synthesis and secretion ([map04925](https://www.kegg.jp/entry/map04925)) |
|  | Organismal Systems | Endocrine system | Cortisol synthesis and secretion ([map04927](https://www.kegg.jp/entry/map04927)) |
|  | Human Diseases | Endocrine and metabolic disease | Cushing syndrome ([map04934](https://www.kegg.jp/entry/map04934)) |
|  | Organismal Systems | Digestive system | Fat digestion and absorption ([map04975](https://www.kegg.jp/entry/map04975)) |
|  | Organismal Systems | Digestive system | Bile secretion ([map04976](https://www.kegg.jp/entry/map04976)) |
| Cluster 2 |  |  |  |
|  | Human Diseases | Cardiovascular disease | Lipid and atherosclerosis ([map05417](https://www.kegg.jp/entry/map05417)) |
|  | Human Diseases | Infectious disease: parasitic | Toxoplasmosis ([map05145](https://www.kegg.jp/entry/map05145)) |
|  | Organismal Systems | Digestive system | Cholesterol metabolism ([map04979](https://www.kegg.jp/entry/map04979)) |
|  | Cellular Processes | Transport and catabolism | Endocytosis ([map04144](https://www.kegg.jp/entry/map04144)) |
|  | Organismal Systems | Endocrine system | Cortisol synthesis and secretion ([map04927](https://www.kegg.jp/entry/map04927)) |
|  | Human Diseases | Endocrine and metabolic disease | Cushing syndrome ([map04934](https://www.kegg.jp/entry/map04934)) |
|  | Organismal Systems | Digestive system | Bile secretion ([map04976](https://www.kegg.jp/entry/map04976)) |
|  | Drug Development | Chronology: Other drugs | Antidyslipidemic agents ([map07052](https://www.kegg.jp/entry/map07052)) |
|  | Organismal Systems | Endocrine system | Ovarian steroidogenesis ([map04913](https://www.kegg.jp/entry/map04913)) |
|  | Organismal Systems | Endocrine system | PPAR signaling pathway ([map03320](https://www.kegg.jp/entry/map03320)) |
|  | Human Diseases | Endocrine and metabolic disease | Insulin resistance ([map04931](https://www.kegg.jp/entry/map04931)) |
| Cluster 3 |  |  |  |
|  | Human Diseases | Cardiovascular disease | Lipid and atherosclerosis ([map05417](https://www.kegg.jp/entry/map05417)) |
|  | Human Diseases | Infectious disease: parasitic | Toxoplasmosis ([map05145](https://www.kegg.jp/entry/map05145)) |
|  | Organismal Systems | Digestive system | Cholesterol metabolism ([map04979](https://www.kegg.jp/entry/map04979)) |
|  | Cellular Processes | Transport and catabolism | Endocytosis ([map04144](https://www.kegg.jp/entry/map04144)) |
|  | Organismal Systems | Endocrine system | Cortisol synthesis and secretion ([map04927](https://www.kegg.jp/entry/map04927)) |
|  | Human Diseases | Endocrine and metabolic disease | Cushing syndrome ([map04934](https://www.kegg.jp/entry/map04934)) |
|  | Organismal Systems | Digestive system | Bile secretion ([map04976](https://www.kegg.jp/entry/map04976)) |
|  | Drug Development | Chronology: Other drugs | Antidyslipidemic agents ([map07052](https://www.kegg.jp/entry/map07052)) |
|  | Organismal Systems | Endocrine system | Ovarian steroidogenesis ([map04913](https://www.kegg.jp/entry/map04913)) |
| Cluster 4 |  |  |  |
|  | Metabolism | Glycan biosynthesis and metabolism | N-Glycan biosynthesis ([hsa00510](https://www.genome.jp/pathway/hsa00510)) |
|  | Metabolism | Carbohydrate metabolism | Butanoate metabolism ([map00650](https://www.genome.jp/pathway/map00650)) |
|  | Organismal Systems | Endocrine system | Thyroid hormone synthesis ([map04918](https://www.genome.jp/pathway/map04918)) |
|  | Metabolism | Carbohydrate metabolism | Citrate cycle (TCA cycle) ([map00020](https://www.kegg.jp/entry/map00020)) |
|  | Metabolism | Energy metabolism | Methane metabolism ([map00680](https://www.kegg.jp/entry/map00680)) |
|  | Metabolism | Energy metabolism | Other carbon fixation pathways ([map00720](https://www.kegg.jp/entry/map00720)) |
|  | Organismal Systems | Nervous system | Cholinergic synapse ([map04725](https://www.kegg.jp/entry/map04725)) |
|  | Metabolism | Global and overview maps | Biosynthesis of secondary metabolites ([map01110](https://www.kegg.jp/entry/map01110)) |
|  | Organismal Systems | Endocrine system | PPAR signaling pathway ([map03320](https://www.kegg.jp/entry/map03320)) |
|  | Human Diseases | Endocrine and metabolic disease | Insulin resistance ([map04931](https://www.kegg.jp/entry/map04931)) |
|  | Environmental Information Processing | Membrane transport | ABC transporters ([map02010](https://www.kegg.jp/entry/map02010)) |
|  | Human Diseases | Neurodegenerative disease | Alzheimer disease ([map05010](https://www.kegg.jp/entry/map05010)) |
|  | Metabolism | Lipid metabolism | Biosynthesis of unsaturated fatty acids ([map01040](https://www.genome.jp/pathway/map01040)) |

**a)** The KEGG PATHWAY database^[5]^ is a collection of manually curated pathway maps representing current knowledge of molecular interactions, reactions, and network relationships across various biological and biomedical areas, including: 1. Metabolism; 2. Genetic Information Processing; 3. Environmental Information Processing; 4. Cellular Processes; 5. Organismal Systems; 6. Human Diseases; and 7. Drug Development.

# Table S5. *β*-coefficients of the variables included in the risk scores for 10-year all-cause mortality prediction derived in 70% of the UK Biobank and used with the same values in the internal and external validation.

| **Characteristics** | **Men aged 50–59 years** | | | **Men aged 60–69 years** | | | **Women aged 50–59 years** | | | **Women aged 60–69 years** | | |
| --- | --- | --- | --- | --- | --- | --- | --- | --- | --- | --- | --- | --- |
|  | **Covariates** | **Biomarkers** | **Covariates + Biomarkers** | **Covariates** | **Biomarkers** | **Covariates + Biomarkers** | **Covariates** | **Biomarkers** | **Covariates + Biomarkers** | **Covariates** | **Biomarkers** | **Covariates + Biomarkers** |
| **Age (per 10 years)** | 0.687 |  | 0.061 | 0.943 |  | 0.083 | 0.553 |  | 0.055 | 0.972 |  | 0.091 |
| **Sex** |  |  |  |  |  |  |  |  |  |  |  |  |
| Women | Ref. |  | Ref. | Ref. |  | Ref. | Ref. |  | Ref. | Ref. |  | Ref. |
| Men | - |  | - | - |  | - | - |  | - | - |  | - |
| **Education level** |  |  |  |  |  |  |  |  |  |  |  |  |
| Medium or low | Ref. |  | Ref. | Ref. |  | Ref. | Ref. |  | Ref. | Ref. |  | Ref. |
| High | -0.003 |  | -0.042 | -0.037 |  | -0.009 | -0.006 |  | -0.015 | -0.094 |  | -0.078 |
| **Smoking status** |  |  |  |  |  |  |  |  |  |  |  |  |
| Never smoker | Ref. |  | Ref. | Ref. |  | Ref. | Ref. |  | Ref. | Ref. |  | Ref. |
| Former smoker | 0.267 |  | 0.215 | 0.309 |  | 0.270 | 0.259 |  | 0.218 | 0.327 |  | 0.281 |
| Current smoker | 1.074 |  | 0.773 | 0.889 |  | 0.651 | 0.928 |  | 0.666 | 1.001 |  | 0.784 |
| **Physical activity** |  |  |  |  |  |  |  |  |  |  |  |  |
| Low | Ref. |  | Ref. | Ref. |  | Ref. | Ref. |  | Ref. | Ref. |  | Ref. |
| Moderate | -0.149 |  | -0.075 | -0.213 |  | -0.156 | -0.140 |  | -0.094 | -0.240 |  | -0.201 |
| High | -0.193 |  | -0.107 | -0.248 |  | -0.179 | -0.281 |  | -0.220 | -0.327 |  | -0.271 |
| **Alcohol consumption** |  |  |  |  |  |  |  |  |  |  |  |  |
| Abstainer | Ref. |  | Ref. | Ref. |  | Ref. | Ref. |  | Ref. | Ref. |  | Ref. |
| Low | 0.198 |  | 0.237 | 0.191 |  | 0.138 | 0.237 |  | 0.141 | 0.229 |  | 0.132 |
| Medium or high | 0.318 |  | 0.243 | 0.293 |  | 0.217 | 0.354 |  | 0.275 | 0.241 |  | 0.165 |
| **Body mass index (**kg/m^2^**)** |  |  |  |  |  |  |  |  |  |  |  |  |
| < 21.5 | 0.649 |  | 0.376 | 0.542 |  | 0.336 | 0.266 |  | 0.210 | 0.227 |  | 0.168 |
| ≥ 21.5 – < 25 | Ref. |  | Ref. | Ref. |  | Ref. | Ref. |  | Ref. | Ref. |  | Ref. |
| ≥ 25 – < 30 | -0.131 |  | 0.009 | -0.025 |  | 0.063 | -0.034 |  | -0.136 | -0.039 |  | -0.078 |
| ≥ 30 – < 35 | -0.054 |  | 0.038 | -0.071 |  | 0.132 | -0.026 |  | -0.249 | -0.108 |  | -0.221 |
| ≥ 35 | 0.350 |  | 0.336 | 0.286 |  | 0.218 | 0.393 |  | 0.115 | 0.227 |  | 0.122 |
| **Treated dyslipidemia** |  |  |  |  |  |  |  |  |  |  |  |  |
| No | Ref. |  | Ref. | Ref. |  | Ref. | Ref. |  | Ref. | Ref. |  | Ref. |
| Yes | -0.058 |  | -0.032 | -0.111 |  | -0.078 | -0.272 |  | -0.147 | -0.064 |  | -0.022 |
| **Hypertension** |  |  |  |  |  |  |  |  |  |  |  |  |
| No | Ref. |  | Ref. | Ref. |  | Ref. | Ref. |  | Ref. | Ref. |  | Ref. |
| Yes | 0.341 |  | 0.190 | 0.189 |  | 0.130 | 0.211 |  | 0.125 | 0.123 |  | 0.056 |
| **Diabetes** |  |  |  |  |  |  |  |  |  |  |  |  |
| No | Ref. |  | Ref. | Ref. |  | Ref. | Ref. |  | Ref. | Ref. |  | Ref. |
| Yes | 0.610 |  | 0.336 | 0.401 |  | 0.250 | 0.295 |  | 0.125 | 0.360 |  | 0.199 |
| **Cardiovascular disease** |  |  |  |  |  |  |  |  |  |  |  |  |
| No | Ref. |  | Ref. | Ref. |  | Ref. | Ref. |  | Ref. | Ref. |  | Ref. |
| Yes | 0.636 |  | 0.509 | 0.553 |  | 0.441 | 0.465 |  | 0.363 | 0.534 |  | 0.459 |
| **Cancer** |  |  |  |  |  |  |  |  |  |  |  |  |
| No | Ref. |  | Ref. | Ref. |  | Ref. | Ref. |  | Ref. | Ref. |  | Ref. |
| Yes | 1.186 |  | 1.010 | 0.712 |  | 0.666 | 1.402 |  | 1.322 | 0.754 |  | 0.732 |
| **Biomarkers** |  |  |  |  |  |  |  |  |  |  |  |  |
| Acetate |  | 0.031 | 0.029 |  | 0.043 | 0.040 |  | - | - |  | - | - |
| Acetoacetate |  | - | - |  | - | - |  | 0.046 | 0.034 |  | 0.056 | 0.047 |
| Acetone |  | 0.066 | 0.085 |  | 0.074 | 0.083 |  | - | - |  | - | - |
| Albumin |  | -0.191 | -0.164 |  | -0.075 | -0.060 |  | -0.204 | -0.186 |  | -0.063 | -0.040 |
| bOHbutyrate |  | 0.012 | 0.039 |  | 0.015 | 0.005 |  | - | - |  | - | - |
| Citrate |  | 0.068 | 0.071 |  | 0.071 | 0.053 |  | - | - |  | - | - |
| Creatinine |  | 0.044 | 0.055 |  | 0.043 | 0.030 |  | 0.052 | 0.045 |  | 0.065 | 0.069 |
| Glucose |  | 0.187 | 0.122 |  | 0.127 | 0.088 |  | 0.038 | 0.021 |  | 0.085 | 0.065 |
| GlycA |  | 0.351 | 0.273 |  | 0.305 | 0.252 |  | 0.376 | 0.290 |  | 0.335 | 0.281 |
| HDL_size |  | 0.051 | 0.073 |  | - | - |  | - | - |  | - | - |
| His |  | -0.066 | -0.046 |  | -0.046 | -0.035 |  | -0.016 | -0.041 |  | -0.037 | -0.029 |
| IDL-CE-pct |  | -0.134 | -0.071 |  | -0.039 | -0.022 |  | - | - |  | - | - |
| L-LDL-CE-pct |  | -0.004 | -0.006 |  |  |  |  | - | - |  | - | - |
| LA-pct |  | -0.277 | -0.167 |  | -0.233 | -0.162 |  | -0.310 | -0.241 |  | -0.257 | -0.204 |
| Lactate |  | 0.095 | 0.055 |  | - | - |  | - | - |  | - | - |
| Leu |  | - | - |  | - | - |  | -0.100 | -0.100 |  | - | - |
| M-LDL-TG-pct |  | - | - |  |  |  |  | 0.184 | 0.140 |  | 0.114 | 0.055 |
| Omega-6/Omega-3 |  | 0.246 | 0.190 |  | 0.223 | 0.187 |  | 0.266 | 0.218 |  | 0.251 | 0.221 |
| PUFA |  | - | - |  | - | - |  | - | - |  | -0.050 | -0.011 |
| S-HDL-CE |  | - | - |  | -0.172 | -0.144 |  | - | - |  | -0.100 | -0.111 |
| S-LDL-CE |  | - | - |  | -0.085 | -0.018 |  | - | - |  | - | - |
| Tyr |  | 0.165 | 0.142 |  | 0.146 | 0.142 |  | - | - |  | - | - |
| Val |  | -0.331 | -0.287 |  | -0.281 | -0.259 |  | -0.095 | -0.044 |  | -0.176 | -0.134 |
| VLDL-size |  | -0.290 | -0.175 |  | -0.201 | -0.177 |  | -0.229 | -0.204 |  | -0.215 | -0.190 |
| XL-HDL-FC |  | 0.225 | 0.201 |  | 0.041 | 0.070 |  | - | - |  | - | - |
| XXL-VLDL-PL-pct |  | 0.226 | 0.211 |  | - | - |  | - | - |  | - | - |

Notes: The method used to calculate the risk score was based on previously published literature.^[6-7]^ The names of the metabolic biomarkers are indicated by representative abbreviations, and the full names of which are detailed in **Table S10** (Supporting Information).

**Table S6. C-statistics of prediction models for 10- and 5-year all-cause mortality in the UK Biobank and ESTHER cohorts, excluding participants on lipid‑lowering therapy**

|  | **C-statistic (*P*)** **^h^** | | |
| --- | --- | --- | --- |
|  | **Derivation set**  **UK Biobank (70%)** | **Internal validation set**  **UK Biobank (30%)** | **External validation set**  **ESTHER** |
| **10-year all-cause mortality  in men aged 50–59 years** | **n=23,148**  **(879 deaths)** | **n=9,911**  **(378 deaths)** | **n=1,072**  **(103 deaths)** |
| Traditional risk factor model **^a^** | 0.783 (0.768, 0.797) | 0.774 (0.751, 0.798) | 0.735 (0.689, 0.781) |
| Metabolomics model **^b^** | 0.688 (0.673, 0.703) | 0.670 (0.645, 0.695) | 0.662 (0.611, 0.713) |
| Combined model | 0.847 (0.832, 0.862) | 0.834 (0.813, 0.855) | 0.817 (0.780, 0.854) |
|  | *ΔC*=0.064; *P*<0.001 | *ΔC*=0.060; *P*<0.001 | *ΔC*=0.082; *P*<0.001 |
| **10-year all-cause mortality  in men aged 60–69 years** | **n=25,666**  **(2,325 deaths)** | **n=10,908**  **(960 deaths)** | **n=1,594**  **(300 deaths)** |
| Traditional risk factor model **^a^** | 0.745 (0.736, 0.753) | 0.735 (0.725, 0.744) | 0.727 (0.695, 0.758) |
| Metabolomics model **^c^** | 0.683 (0.672, 0.693) | 0.669 (0.658, 0.681) | 0.653 (0.622, 0.684) |
| Combined model | 0.800 (0.788, 0.811) | 0.785 (0.775, 0.794) | 0.784 (0.753, 0.815) |
|  | *ΔC*=0.055; *P*<0.001 | *ΔC*=0.050; *P*<0.001 | *ΔC*=0.057; *P*<0.001 |
| **10-year all-cause mortality  in women aged 50–59 years** | **n=32,173**  **(727 deaths)** | **n=13,839**  **(352 deaths)** | **n=1,480**  **(82 deaths)** |
| Traditional risk factor model **^a^** | 0.749 (0.731, 0.767) | 0.735 (0.707, 0.764) | 0.727 (0.674, 0.780) |
| Metabolomics model **^d^** | 0.675 (0.656, 0.694) | 0.667 (0.639, 0.695) | 0.648 (0.593, 0.703) |
| Combined model | 0.803 (0.786, 0.820) | 0.790 (0.765, 0.815) | 0.766 (0.717, 0.810) |
|  | *ΔC*=0.054; *P*<0.001 | *ΔC*=0.055; *P*<0.001 | *ΔC*=0.039; *P*=0.052 |
| **10-year all-cause mortality  in women aged 60–69 years** | **n=33,551**  **(1,700 deaths)** | **n=14,520**  **(751 deaths)** | **n=1,962**  **(207 deaths)** |
| Traditional risk factor model **^a^** | 0.752 (0.740, 0.764) | 0.736 (0.719, 0.753) | 0.716 (0.681, 0.751) |
| Metabolomics model **^e^** | 0.669 (0.656, 0.682) | 0.656 (0.638, 0.674) | 0.640 (0.604, 0.676) |
| Combined model | 0.800 (0.788, 0.812) | 0.778 (0.762, 0.794) | 0.770 (0.738, 0.802) |
|  | *ΔC*=0.048; *P*<0.001 | *ΔC*=0.042; *P*<0.001 | *ΔC*=0.054; *P*<0.001 |
| **10-year all-cause mortality  in total study population** | **n=114,538**  **(5,631 deaths)** | **n=49,178**  **(2,441 deaths)** | **n=6,108**  **(692 deaths)** |
| Traditional risk factor model **^a^** | 0.759 (0.752, 0.766) | 0.724 (0.716, 0.732) | 0.722 (0.701, 0.743) |
| Metabolomics model **^f^** | 0.692 (0.685, 0.699) | 0.681 (0.672, 0.690) | 0.663 (0.645, 0.681) |
| Combined model | 0.837 (0.831, 0.844) | 0.800 (0.791, 0.809) | 0.791 (0.770, 0.812) |
|  | *ΔC*=0.078; *P*<0.001 | *ΔC*=0.076; *P*<0.001 | *ΔC*=0.069; *P*<0.001 |
| **5-year all-cause mortality  in total study population** | **n=114,538**  **(1,955 deaths)** | **n=49,178**  **(884 deaths)** | **n=6,108**  **(277 deaths)** |
| Traditional risk factor model **^a^** | 0.763 (0.751, 0.775) | 0.733 (0.719, 0.747) | 0.736 (0.707, 0.765) |
| Metabolomics model **^g^** | 0.704 (0.692, 0.716) | 0.686 (0.672, 0.700) | 0.675 (0.647, 0.703) |
| Combined model | 0.861 (0.852, 0.870) | 0.823 (0.809, 0.837) | 0.817 (0.791, 0.843) |
|  | *ΔC*=0.098; *P*<0.001 | *ΔC*=0.090; *P*<0.001 | *ΔC*=0.081; *P*<0.001 |

**a)** Variables of the traditional risk factor models are: age, sex, education level, smoking status, physical activity, alcohol consumption, body mass index, hypertension, diabetes, cardiovascular disease, and cancer.

**b)** Metabolites included in this model were (see **Table S10** (Supporting Information) for abbreviations): GlycA, XXL-VLDL-PL-pct, XL-HDL-FC, Omega-6/Omega-3, Tyr, Glucose, Acetone, HDL-size, Citrate, Lactate, Creatinine, bOHbutyrate, Acetate, L-LDL-CE-pct, His, IDL-CE-pct, Albumin, LA-pct, VLDL-size, Val.

**c)** Metabolites included in this model were (see **Table S10** (Supporting Information) for abbreviations): GlycA, Omega-6/Omega-3, Tyr, Glucose, Acetone, XL-HDL-FC, Citrate, Acetate, Creatinine, bOHbutyrate, S-LDL-CE, IDL-CE-pct, His, Albumin, S-HDL-CE, LA-pct, VLDL-size, Val.

**d)** Metabolites included in this model were (see **Table S10** (Supporting Information) for abbreviations): GlycA, Omega-6/Omega-3, M-LDL-TG-pct, Creatinine, Acetoacetate, Glucose, His, Val, Leu, Albumin, VLDL-size, LA-pct.

**e)** Metabolites included in this model were (see **Table S10** (Supporting Information) for abbreviations): GlycA, Omega-6/Omega-3, Creatinine, Glucose, M-LDL-TG-pct, Acetoacetate, PUFA, His, Albumin, S-HDL-CE, Val, VLDL-size, LA-pct.

**f)/g)** Metabolites included in this model were (see **Table S10** (Supporting Information) for abbreviations): GlycA, XXL-VLDL-PL-pct, XL-HDL-FC, Omega-6/Omega-3, Tyr, Glucose, Acetone, HDL-size, Citrate, Lactate, Creatinine, bOHbutyrate, Acetate, L-LDL-CE-pct, S-LDL-CE, IDL-CE-pct, M-LDL-TG-pct, Acetoacetate, PUFA, His, Val, Leu, Albumin, S-HDL-CE, LA-pct, VLDL-size.

**h)** The c-statistic of the combined model was tested against the c-statistic of the traditional risk factor model with the DeLong test.

# Table S7. Reclassification improvements in 10- and 5-year mortality prediction by adding metabolomic biomarkers to conventional risk models: evidence from the UK Biobank and ESTHER study.

|  | **Categorial NRI** **^f^** | **Continuous NRI** | **IDI** |
| --- | --- | --- | --- |
| **10-year all-cause mortality**  **in men aged 50–59 years** **^a^** |  |  |  |
| UK Biobank (70%) | 17.5±5.3%, *P*<0.001 | 37.5±10.9%, *P*<0.001 | 10.7±2.9%, *P*<0.001 |
| UK Biobank (30%) | 12.6±4.3%, *P*<0.001 | 32.0±9.1%, *P*<0.001 | 9.9±2.7%, *P*<0.001 |
| ESTHER Study | 18.9±5.9%, *P*<0.001 | 41.3±11.1%, *P*<0.001 | 12.4±3.2%, *P*<0.001 |
| **10-year all-cause mortality**  **in men aged 60–69 years** **^b^** |  |  |  |
| UK Biobank (70%) | 15.0±4.7%, *P*<0.001 | 30.1±7.5%, *P*<0.001 | 6.1±2.0%, *P*<0.001 |
| UK Biobank (30%) | 9.4±3.4%, *P*<0.001 | 26.5±6.8%, *P*<0.001 | 5.0±1.6%, *P*<0.001 |
| ESTHER Study | 8.2±2.5%, *P*<0.001 | 25.8±5.0%, *P*<0.001 | 4.6±1.0%, *P*<0.001 |
| **10-year all-cause mortality**  **in women aged 50–59 years** **^c^** |  |  |  |
| UK Biobank (70%) | 16.3±5.1%, *P*<0.001 | 31.4±8.1%, *P*<0.001 | 6.4±2.3%, *P*<0.001 |
| UK Biobank (30%) | 10.7±2.5%, *P*<0.001 | 29.7±7.9%, *P*<0.001 | 5.8±1.7%, *P*<0.001 |
| ESTHER Study | -0.2±0.4%, *P*=0.146 | 1.2±0.8%, *P*=0.045 | -0.05±0.08%, *P*=0.086 |
| **10-year all-cause mortality**  **in women aged 60–69 years** **^d^** |  |  |  |
| UK Biobank (70%) | 11.5±3.9%, *P*<0.001 | 26.7±7.4%, *P*<0.001 | 5.9±1.7%, *P*<0.001 |
| UK Biobank (30%) | 9.1±3.0%, *P*<0.001 | 24.4±5.1%, *P*<0.001 | 4.8±1.2%, *P*<0.001 |
| ESTHER Study | 14.5±4.3%, *P*<0.001 | 31.7±8.3%, *P*<0.001 | 6.2±1.8%, *P*<0.001 |
| **10-year all-cause mortality**  **in total study population** **^e^** |  |  |  |
| UK Biobank (70%) | 18.3±5.5%, *P*<0.001 | 41.6±11.8%, *P*<0.001 | 11.7±3.4%, *P*<0.001 |
| UK Biobank (30%) | 16.1±4.2%, *P*<0.001 | 38.2±8.4%, *P*<0.001 | 9.5±2.5%, *P*<0.001 |
| ESTHER Study | 14.7±3.8%, *P*<0.001 | 30.4±7.7%, *P*<0.001 | 8.3±1.6%, *P*<0.001 |
| **5-year all-cause mortality**  **in total study population** **^e^** |  |  |  |
| UK Biobank (70%) | 24.7±6.8%, *P*<0.001 | 42.2±12.4%, *P*<0.001 | 12.3±3.6%, *P*<0.001 |
| UK Biobank (30%) | 20.3±5.5%, *P*<0.001 | 39.7±10.1%, *P*<0.001 | 10.7±3.1%, *P*<0.001 |
| ESTHER Study | 19.1±4.7%, *P*<0.001 | 33.4±9.2%, *P*<0.001 | 9.8±2.2%, *P*<0.001 |

Notes: Data are expressed as point estimates with 95% confidence intervals, presented as estimate ± half the confidence interval width. Both the conventional risk factor model and the extended model incorporating metabolomic biomarkers were developed in the derivation cohort (70% of UK Biobank) and validated in the internal validation cohort (30% of UK Biobank) and the external ESTHER study. The conventional risk factor model, included age, sex, education level, smoking status, physical activity, alcohol consumption, body mass index, treated dyslipidemia, hypertension, diabetes, cardiovascular disease, and cancer.

**a)** 20 metabolic biomarkers, **b)** 18 metabolic biomarkers, **c)** 12 metabolic biomarkers, **d)** 13 metabolic biomarkers were used in the younger men, older men, younger women and older women groups, respectively.

**e)** The overlap of 26 unique biomarkers across four subgroups were used in the total study populations.

**f)** Categorical reclassification was assessed for three risk categories (≤ 5%, >5%–10%, >10%) based on the conventional risk factors and the biomarker score.^[8]^ IDI, integrated discrimination improvement;^[9]^ NRI, net reclassification improvement.^[10]^

# Table S8. Equations used to calculate the metabolomics-based mortality risk prediction (MetaboMR) clocks 1 and 2.

| **Subgroup** | **Intercept and *β*-coefficients** |
| --- | --- |
| **Step 1: Elastic net models** ^a^ |  |
| **To derive MetaboMR clock1** |  |
| Men aged 50–59 years | Raw metabolomic age1 = 55.123 + 0.017×Acetate + 0.024×Acetone - 0.259×Albumin + 0.040×bOHbutyrate + 0.224×Citrate + 0.001×Creatinine + 0.077×Glucose + 0.028×GlycA + 0.166×HDL-size - 0.062×His - 0.035×IDL-CE-pct - 0.014×L-LDL-CE-pct - 0.014×LA-pct + 0.012×Lactate + 0.257×Omega-6/Omega-3 + 0.200×Tyr - 0.203×Val - 0.093×VLDL-size + 0.135×XL-HDL-FC + 0.054×XXL-VLDL-PL-pct |
| Men aged 60–69 years | Raw metabolomic age1 = 64.116 + 0.011×Acetate + 0.105×Acetone - 0.212×Albumin + 0.186×bOHbutyrate + 0.251×Citrate + 0.120×Creatinine + 0.064×Glucose + 0.060×GlycA - 0.058×His - 0.002×IDL-CE-pct - 0.038×LA-pct + 0.222×Omega-6/Omega-3 - 0.010×S-HDL-CE - 0.322×S-LDL-CE + 0.135×Tyr - 0.180×Val - 0.021×VLDL-size + 0.026×XL-HDL-FC |
| Women aged 50–59 years | Raw metabolomic age1 = 54.834 + 0.102×Acetoacetate - 0.047×Albumin + 0.070×Creatinine + 0.129×Glucose + 0.129×GlycA - 0.078×His + 0.034×LA-pct - 0.181×Leu + 0.063×M-LDL-TG-pct + 0.359×Omega-6/Omega-3 - 0.138×Val - 0.085×VLDL-size |
| Women aged 60–69 years | Raw metabolomic age1 = 63.907 + 0.079×Acetoacetate - 0.232×Albumin + 0.179×Creatinine + 0.099×Glucose + 0.081×GlycA - 0.071×His - 0.015×LA-pct + 0.188×M-LDL-TG-pct + 0.169×Omega-6/Omega-3 - 0.023×PUFA - 0.060×S-HDL-CE - 0.143×Val - 0.124×VLDL-size |
| **To derive MetaboMR clock2** |  |
| Men aged 50–59 years | Raw metabolomic age2 = 54.532 - 0.001×(Education=High) + 0.549×(Smoking=Former) + 0.063×(Smoking=Current) - 0.063×(Physical activity=Moderate) - 0.119×(Physical activity=High) + 0.146×(Alcohol consumption=Low) + 0.109×(Alcohol consumption=Medium or High) + 0.100×(BMI< 21.5 kg/m2) + 0.042×(BMI ≥ 25 – < 30 kg/m2) + 0.241×(BMI ≥ 30 – < 35 kg/m2) + 0.435×(BMI ≥ 35 kg/m2) - 0.673×(Treated dyslipidemia) + 0.510×Hypertension + 0.007×Diabetes + 0.967×(Cardiovascular disease) + 0.881×Cancer + 0.013×Acetate + 0.002×Acetone - 0.278×Albumin + 0.043×bOHbutyrate + 0.252×Citrate + 0.003×Creatinine + 0.016×Glucose + 0.021×GlycA + 0.108×HDL-size - 0.040×His - 0.025×IDL-CE-pct - 0.012×L-LDL-CE-pct - 0.047×LA-pct + 0.026×Lactate + 0.214×Omega-6/Omega-3 + 0.165×Tyr - 0.157×Val - 0.010×VLDL-size + 0.067×XL-HDL-FC + 0.050×XXL-VLDL-PL-pct |
| Men aged 60–69 years | Raw metabolomic age2 = 63.589 - 0.116×(Education=High) + 0.347×(Smoking=Former) + 0.145×(Smoking=Current) - 0.040×(Physical activity=Moderate) - 0.124×(Physical activity=High) + 0.047×(Alcohol consumption=Low) + 0.257×(Alcohol consumption=Medium or High) + 0.010×(BMI< 21.5 kg/m2) + 0.001×(BMI ≥ 25 – < 30 kg/m2) + 0.162×(BMI ≥ 30 – < 35 kg/m2) + 0.531×(BMI ≥ 35 kg/m2) - 0.389×(Treated dyslipidemia) + 0.457×Hypertension + 0.130×Diabetes + 0.913×(Cardiovascular disease) + 0.884×Cancer + 0.003×Acetate + 0.109×Acetone - 0.236×Albumin + 0.191×bOHbutyrate + 0.239×Citrate + 0.089×Creatinine + 0.026×Glucose + 0.037×GlycA - 0.042×His - 0.040×IDL-CE-pct - 0.036×LA-pct + 0.181×Omega-6/Omega-3 - 0.024×S-HDL-CE - 0.187×S-LDL-CE + 0.143×Tyr - 0.163×Val - 0.003×VLDL-size + 0.125×XL-HDL-FC |
| Women aged 50–59 years | Raw metabolomic age2 = 54.480 - 0.006×(Education=High) + 0.363×(Smoking=Former) + 0.154×(Smoking=Current) - 0.070×(Physical activity=Moderate) - 0.004×(Physical activity=High) + 0.058×(Alcohol consumption=Low) + 0.055×(Alcohol consumption=Medium or High) + 0.008×(BMI< 21.5 kg/m2) - 0.107×(BMI ≥ 25 – < 30 kg/m2) - 0.311×(BMI ≥ 30 – < 35 kg/m2) + 0.535×(BMI ≥ 35 kg/m2) - 0.787×(Treated dyslipidemia) + 0.582×Hypertension + 0.058×Diabetes + 0.874×(Cardiovascular disease) + 0.731×Cancer + 0.098×Acetoacetate - 0.079×Albumin + 0.065×Creatinine + 0.083×Glucose + 0.127×GlycA - 0.063×His + 0.090×LA-pct - 0.195×Leu + 0.118×M-LDL-TG-pct + 0.341×Omega-6/Omega-3 - 0.176×Val - 0.102×VLDL-size |
| Women aged 60–69 years | Raw metabolomic age2 = 63.620 - 0.190×(Education=High) + 0.067×(Smoking=Former) + 0.465×(Smoking=Current) - 0.085×(Physical activity=Moderate) - 0.087×(Physical activity=High) + 0.164×(Alcohol consumption=Low) + 0.356×(Alcohol consumption=Medium or High) + 0.037×(BMI< 21.5 kg/m2) - 0.045×(BMI ≥ 25 – < 30 kg/m2) - 0.132×(BMI ≥ 30 – < 35 kg/m2) + 0.639×(BMI ≥ 35 kg/m2) - 0.787×(Treated dyslipidemia) + 0.671×Hypertension + 0.083×Diabetes + 0.848×(Cardiovascular disease) + 0.732×Cancer + 0.080×Acetoacetate - 0.274×Albumin + 0.157×Creatinine + 0.056×Glucose + 0.029×GlycA - 0.047×His - 0.062×LA-pct + 0.073×M-LDL-TG-pct + 0.088×Omega-6/Omega-3 - 0.146×PUFA - 0.009×S-HDL-CE - 0.122×Val - 0.151×VLDL-size |
| **Step 2: Linear regression models ^b^** | |
| **To derive MetaboMR clock1** |  |
| Men aged 50–59 years | MetaboMR clock1 = -1.128 + 1.021 × Raw metabolomic age1 |
| Men aged 60–69 years | MetaboMR clock1 = -0.819 + 1.013 × Raw metabolomic age1 |
| Women aged 50–59 years | MetaboMR clock1 = -0.752 + 1.003 × Raw metabolomic age1 |
| Women aged 60–69 years | MetaboMR clock1 = -0.559 + 1.009 × Raw metabolomic age1 |
| **To derive MetaboMR clock2** |  |
| Men aged 50–59 years | MetaboMR clock2 = -1.034 + 1.019 × Raw metabolomic age2 |
| Men aged 60–69 years | MetaboMR clock2 = -0.941 + 1.015 × Raw metabolomic age2 |
| Women aged 50–59 years | MetaboMR clock2 = -0.803 + 1.017 × Raw metabolomic age2 |
| Women aged 60–69 years | MetaboMR clock2 = -0.513 + 1.005 × Raw metabolomic age2 |

Abbreviations: MetaboMR clock1, metabolomics-based mortality risk prediction clock 1; MetaboMR clock2, metabolomics-based mortality risk prediction clock 2.

Notes: The names of the metabolic biomarkers are indicated by representative abbreviations, and the full names of which are detailed in **Table S10** (Supporting Information).

**a)** The intercept and coefficients stem from an elastic net model, in which chronological age was the dependent variable. Raw metabolomic age1 was derived solely from the selected metabolomic biomarkers in each stratum. Raw metabolomic age2 was derived from both the selected metabolomic biomarkers and traditional mortality risk factors in each stratum. The estimates stem from the training set (70% of the UK Biobank).

**b)** The intercept and coefficients stem from a linear regression model with chronological age as the dependent variable and the raw metabolomic age 1 or 2 as the independent variable. The estimates stem from the training set (70% of the UK Biobank).

**Table S9. Association of metabolomic age acceleration based on metabolomic biomarkers only (MetAA1) and based on both biomarkers and traditional risk factors (MetAA2) with 10-year all-cause mortality, excluding participants on lipid‑lowering therapy**

| **MetAA / Study  population** | **Training set**  **UK Biobank (70%)** | | |  | **Internal validation set**  **UK Biobank (30%)** | | |  | **External validation set**  **ESTHER** | | |
| --- | --- | --- | --- | --- | --- | --- | --- | --- | --- | --- | --- |
|  | **Mean  MetAA  ± SD  (years)** | **Crude HR  per 1-year  MetAA  increase ^a^** | **Adjusted HR  per 1-year  MetAA  increase ^b^** |  | **Mean  MetAA  ± SD  (years)** | **Crude HR  per 1-year  MetAA  increase ^a^** | **Adjusted HR  per 1-year  MetAA  increase ^b^** |  | **Mean  MetAA  ± SD  (years)** | **Crude HR  per 1-year  MetAA**  **increase ^a^** | **Adjusted HR  per 1-year  MetAA  increase ^b^** |
| **MetAA1** |  |  |  |  |  |  |  |  |  |  |  |
| **Men aged 50–59 years** | 0.00±5.68 | **1.17 (1.13–1.21)** | **1.14 (1.10–1.18)** |  | 0.23±5.92 | **1.21 (1.14–1.28)** | **1.18 (1.12–1.24)** |  | 0.37±5.78 | **1.20 (1.09–1.33)** | **1.17 (1.05–1.30)** |
| **Men aged 60–69 years** | 0.00±5.79 | **1.23 (1.20–1.26)** | **1.20 (1.18–1.22)** |  | 0.28±6.05 | **1.20 (1.17–1.23)** | **1.19 (1.16–1.22)** |  | 0.48±5.91 | **1.21 (1.15–1.28)** | **1.20 (1.14–1.27)** |
| **Women aged 50–59 years** | 0.00±5.71 | **1.16 (1.12–1.20)** | **1.13 (1.08–1.17)** |  | 0.14±5.64 | **1.15 (1.09–1.22)** | **1.11 (1.07–1.15)** |  | 0.44±6.12 | **1.14 (1.03–1.26)** | **1.12 (1.01–1.24)** |
| **Women aged 60–69 years** | 0.00±5.73 | **1.19 (1.16–1.22)** | **1.17 (1.14–1.20)** |  | 0.19±5.84 | **1.20 (1.16–1.24)** | **1.17 (1.13–1.21)** |  | 0.50±5.75 | **1.16 (1.08–1.23)** | **1.11 (1.04–1.19)** |
| **Total study population** | 0.00±5.78 | **1.14 (1.13–1.15)** | **1.12 (1.11–1.13)** |  | 0.20±5.61 | **1.13 (1.12–1.14)** | **1.11 (1.10–1.12)** |  | 0.45±6.06 | **1.12 (1.10–1.14)** | **1.10 (1.08–1.12)** |
| **MetAA2** |  |  |  |  |  |  |  |  |  |  |  |
| **Men aged 50–59 years** | 0.00±5.39 | **1.15 (1.11–1.19)** | N.A.^c^ |  | 0.14±5.70 | **1.18 (1.13–1.23)** | N.A.^c^ |  | 0.31±5.60 | **1.16 (1.05–1.29)** | N.A.^c^ |
| **Men aged 60–69 years** | 0.00±5.55 | **1.22 (1.20–1.24)** | N.A.^c^ |  | 0.21±5.81 | **1.18 (1.15–1.21)** | N.A.^c^ |  | 0.44±5.79 | **1.18 (1.12–1.24)** | N.A.^c^ |
| **Women aged 50–59 years** | 0.00±5.63 | **1.15 (1.10–1.20)** | N.A.^c^ |  | 0.10±5.47 | **1.12 (1.08–1.16)** | N.A.^c^ |  | 0.36±6.11 | **1.13 (1.04–1.25)** | N.A.^c^ |
| **Women aged 60–69 years** | 0.00±5.68 | **1.18 (1.15–1.21)** | N.A.^c^ |  | 0.18±5.74 | **1.16 (1.13–1.19)** | N.A.^c^ |  | 0.41±5.95 | **1.13 (1.06–1.20)** | N.A.^c^ |
| **Total study population** | 0.00±5.54 | **1.13 (1.12–1.14)** | N.A.^c^ |  | 0.16±5.59 | **1.12 (1.11–1.13)** | N.A.^c^ |  | 0.35±5.76 | **1.10 (1.08–1.12)** | N.A.^c^ |

Abbreviations: CI, confidence interval; HR, hazard ratio; MetAA1, metabolomic age acceleration.

Notes: Printed in bold: Statistically significant (*P*<0.05).

**a)** HRs for mortality were estimated per 1-year increase in metabolomic age acceleration (MetAA1), reflecting the relative increase in mortality risk for each year that a person’s metabolomic age exceeds their chronological age.

**b)** The adjusted Cox proportional hazards regression model included the following covariates: chronological age, sex, education level, smoking status, physical activity, alcohol consumption, body mass index, hypertension, diabetes, cardiovascular disease, and cancer.

**c)** As the MetaboMR clock2 already includes the traditional risk factors, it cannot be further adjusted for them.

# Table S10. Abbreviations, full names, classes and distributions of 249 metabolomic biomarkers in the UK Biobank and ESTHER study.

| **Metabolic biomarkers** | **Full Name** | **Class** | **UK Biobank  n (%)** | **UK Biobank  mean (SD)** | **ESTHER  n (%)** | **ESTHER mean (SD)** | **Unit** |
| --- | --- | --- | --- | --- | --- | --- | --- |
| Acetate | Acetate | Ketone bodies | 208,974 (99.9) | 0.02 (0.03) | 6,820 (100) | 0.04 (0.06) | mmol/l |
| Acetoacetate | Acetoacetate | Ketone bodies | 209,136 (99.9) | 0.01 (0.01) | 6,818 (99.9) | 0.01 (0.01) | mmol/l |
| Acetone | Acetone | Ketone bodies | 209,141 (99.9) | 0.01 (0.01) | 6,820 (100) | 0.02 (0.01) | mmol/l |
| Ala | Alanine | Amino acids | 209,061 (99.9) | 0.30 (0.08) | 6,775 (99.3) | 0.31 (0.08) | mmol/l |
| Albumin | Albumin | Fluid balance | 209,116 (99.9) | 39.24 (3.37) | 6,820 (100) | 44.70 (4.49) | g/l |
| ApoA1 | Apolipoprotein A1 | Apolipoproteins | 209,144 (100) | 1.47 (0.25) | 6,820 (100) | 1.52 (0.28) | g/l |
| ApoB | Apolipoprotein B | Apolipoproteins | 209,144 (100) | 0.86 (0.20) | 6,820 (100) | 1.10 (0.26) | g/l |
| ApoB/ApoA1 | Ratio of apolipoprotein B to apolipoprotein A1 | Apolipoproteins | 209,144 (100) | 0.60 (0.16) | 6,820 (100) | 0.75 (0.24) | ratio |
| bOHbutyrate | 3-Hydroxybutyrate | Ketone bodies | 205,167 (98.1) | 0.06 (0.06) | 6,769 (99.3) | 0.09 (0.07) | mmol/l |
| Cholines | Total cholines | Other lipids | 208,978 (99.9) | 2.60 (0.42) | 6,794 (99.6) | 2.94 (0.46) | mmol/l |
| Citrate | Citrate | Glycolysis related metabolites | 209,126 (99.9) | 0.07 (0.01) | 6,723 (98.6) | 0.06 (0.02) | mmol/l |
| Clinical-LDL-C | Clinical LDL cholesterol | Cholesterol | 209,144 (100) | 2.58 (0.75) | 6,820 (100) | 3.47 (0.99) | mmol/l |
| Creatinine | Creatinine | Fluid balance | 203,993 (97.5) | 0.07 (0.02) | 6,535 (96.1) | 0.06 (0.02) | mmol/l |
| DHA | Docosahexaenoic acid | Fatty acids | 208,978 (99.9) | 0.24 (0.09) | 6,794 (99.6) | 0.27 (0.08) | mmol/l |
| DHA-pct | Ratio of docosahexaenoic acid to total fatty acids | Fatty acid ratios | 208,978 (99.9) | 2.02 (0.69) | 6,794 (99.6) | 1.94 (0.51) | ratio |
| Gln | Glutamine | Amino acids | 208,489 (99.7) | 0.56 (0.08) | 6,627 (97.2) | 0.54 (0.11) | mmol/l |
| Glucose | Glucose | Glycolysis related metabolites | 208,766 (99.8) | 3.76 (1.22) | 6,584 (96.5) | 3.79 (2.17) | mmol/l |
| Gly | Glycine | Amino acids | 208,835 (99.8) | 0.17 (0.07) | 6,780 (99.4) | 0.36 (0.11) | mmol/l |
| GlycA | Glycoprotein acetyls | Inflammation | 209,143 (99.9) | 0.82 (0.12) | 6,820 (100) | 0.87 (0.13) | mmol/l |
| HDL-C | HDL cholesterol | Cholesterol | 209,144 (100) | 1.33 (0.33) | 6,820 (100) | 1.39 (0.34) | mmol/l |
| HDL-CE | Cholesteryl esters in HDL | Cholesteryl esters | 209,144 (100) | 1.04 (0.26) | 6,820 (100) | 1.07 (0.27) | mmol/l |
| HDL-FC | Free cholesterol in HDL | Free cholesterol | 209,144 (100) | 0.30 (0.07) | 6,820 (100) | 0.33 (0.07) | mmol/l |
| HDL-L | Total lipids in HDL | Total lipids | 209,144 (100) | 3.05 (0.65) | 6,820 (100) | 3.17 (0.69) | mmol/l |
| HDL-P | Concentration of HDL particles | Lipoprotein particle concentrations | 209,144 (100) | 0.02 (0.00) | 6,820 (100) | 0.02 (0.00) | mmol/l |
| HDL-PL | Phospholipids in HDL | Phospholipids | 209,144 (100) | 1.57 (0.32) | 6,820 (100) | 1.60 (0.35) | mmol/l |
| HDL-size | Average diameter for HDL particles | Lipoprotein particle sizes | 209,144 (100) | 9.64 (0.20) | 6,820 (100) | 9.67 (0.17) | nm |
| HDL-TG | Triglycerides in HDL | Triglycerides | 209,144 (100) | 0.15 (0.05) | 6,820 (100) | 0.17 (0.07) | mmol/l |
| His | Histidine | Amino acids | 208,869 (99.8) | 0.07 (0.01) | 6,785 (99.5) | 0.09 (0.02) | mmol/l |
| IDL-C | Cholesterol in IDL | IDL (average diameter 28.6 nm) | 209,144 (100) | 0.85 (0.22) | 6,820 (100) | 1.11 (0.30) | mmol/l |
| IDL-C-pct | Cholesterol to total lipids ratio in IDL | IDL ratios | 209,144 (100) | 67.58 (3.06) | 6,820 (100) | 67.57 (3.10) | % |
| IDL-CE | Cholesteryl esters in IDL | IDL (average diameter 28.6 nm) | 209,144 (100) | 0.62 (0.17) | 6,820 (100) | 0.82 (0.22) | mmol/l |
| IDL-CE-pct | Cholesteryl esters to total lipids ratio in IDL | IDL ratios | 209,144 (100) | 49.87 (2.49) | 6,820 (100) | 50.18 (2.66) | % |
| IDL-FC | Free cholesterol in IDL | IDL (average diameter 28.6 nm) | 209,144 (100) | 0.22 (0.06) | 6,820 (100) | 0.29 (0.08) | mmol/l |
| IDL-FC-pct | Free cholesterol to total lipids ratio in IDL | IDL ratios | 209,144 (100) | 17.71 (1.13) | 6,820 (100) | 17.40 (0.92) | % |
| IDL-L | Total lipids in IDL | IDL (average diameter 28.6 nm) | 209,144 (100) | 1.25 (0.30) | 6,820 (100) | 1.64 (0.41) | mmol/l |
| IDL-P | Concentration of IDL particles | IDL (average diameter 28.6 nm) | 209,144 (100) | 0.01 (0.01) | 6,820 (100) | 0.01 (0.01) | mmol/l |
| IDL-PL | Phospholipids in IDL | IDL (average diameter 28.6 nm) | 209,144 (100) | 0.30 (0.07) | 6,820 (100) | 0.39 (0.10) | mmol/l |
| IDL-PL-pct | Phospholipids to total lipids ratio in IDL | IDL ratios | 209,144 (100) | 23.84 (0.92) | 6,820 (100) | 23.79 (0.78) | % |
| IDL-TG | Triglycerides in IDL | IDL (average diameter 28.6 nm) | 209,144 (100) | 0.10 (0.03) | 6,820 (100) | 0.14 (0.05) | mmol/l |
| IDL-TG-pct | Triglycerides to total lipids ratio in IDL | IDL ratios | 209,144 (100) | 8.59 (2.55) | 6,820 (100) | 8.64 (2.79) | % |
| Ile | Isoleucine | Branched−chain amino acids | 209,111 (99.9) | 0.05 (0.02) | 6,820 (100) | 0.07 (0.02) | mmol/l |
| L-HDL-C | Cholesterol in large HDL | Large HDL (average diameter 12.1 nm) | 209,144 (100) | 0.30 (0.17) | 6,820 (100) | 0.32 (0.15) | mmol/l |
| L-HDL-C-pct | Cholesterol to total lipids ratio in large HDL | Large HDL ratios | 209,144 (100) | 43.64 (5.64) | 6,820 (100) | 44.61 (4.81) | % |
| L-HDL-CE | Cholesteryl esters in large HDL | Large HDL (average diameter 12.1 nm) | 209,144 (100) | 0.23 (0.13) | 6,820 (100) | 0.25 (0.12) | mmol/l |
| L-HDL-CE-pct | Cholesteryl esters to total lipids ratio in large HDL | Large HDL ratios | 209,144 (100) | 33.68 (4.84) | 6,820 (100) | 34.02 (4.46) | % |
| L-HDL-FC | Free cholesterol in large HDL | Large HDL (average diameter 12.1 nm) | 209,144 (100) | 0.07 (0.04) | 6,820 (100) | 0.08 (0.03) | mmol/l |
| L-HDL-FC-pct | Free cholesterol to total lipids ratio in large HDL | Large HDL ratios | 209,144 (100) | 9.97 (1.21) | 6,820 (100) | 10.58 (1.02) | % |
| L-HDL-L | Total lipids in large HDL | Large HDL (average diameter 12.1 nm) | 209,144 (100) | 0.66 (0.33) | 6,820 (100) | 0.71 (0.30) | mmol/l |
| L-HDL-P | Concentration of large HDL particles | Large HDL (average diameter 12.1 nm) | 209,144 (100) | 0.01 (0.01) | 6,820 (100) | 0.01 (0.01) | mmol/l |
| L-HDL-PL | Phospholipids in large HDL | Large HDL (average diameter 12.1 nm) | 209,144 (100) | 0.33 (0.15) | 6,820 (100) | 0.35 (0.14) | mmol/l |
| L-HDL-PL-pct | Phospholipids to total lipids ratio in large HDL | Large HDL ratios | 209,144 (100) | 50.70 (3.12) | 6,820 (100) | 49.03 (2.57) | % |
| L-HDL-TG | Triglycerides in large HDL | Large HDL (average diameter 12.1 nm) | 209,144 (100) | 0.03 (0.01) | 6,820 (100) | 0.04 (0.02) | mmol/l |
| L-HDL-TG-pct | Triglycerides to total lipids ratio in large HDL | Large HDL ratios | 209,144 (100) | 5.66 (3.27) | 6,820 (100) | 6.35 (3.57) | % |
| L-LDL-C | Cholesterol in large LDL | Large LDL (average diameter 25.5 nm) | 209,144 (100) | 1.15 (0.29) | 6,820 (100) | 1.51 (0.38) | mmol/l |
| L-LDL-C-pct | Cholesterol to total lipids ratio in large LDL | Large LDL ratios | 209,144 (100) | 71.09 (1.93) | 6,820 (100) | 71.25 (2.39) | % |
| L-LDL-CE | Cholesteryl esters in large LDL | Large LDL (average diameter 25.5 nm) | 209,144 (100) | 0.85 (0.22) | 6,820 (100) | 1.11 (0.27) | mmol/l |
| L-LDL-CE-pct | Cholesteryl esters to total lipids ratio in large LDL | Large LDL ratios | 209,144 (100) | 52.64 (1.35) | 6,820 (100) | 52.55 (1.58) | % |
| L-LDL-FC | Free cholesterol in large LDL | Large LDL (average diameter 25.5 nm) | 209,144 (100) | 0.30 (0.08) | 6,820 (100) | 0.40 (0.11) | mmol/l |
| L-LDL-FC-pct | Free cholesterol to total lipids ratio in large LDL | Large LDL ratios | 209,144 (100) | 18.45 (1.35) | 6,820 (100) | 18.71 (1.28) | % |
| L-LDL-L | Total lipids in large LDL | Large LDL (average diameter 25.5 nm) | 209,144 (100) | 1.61 (0.39) | 6,820 (100) | 2.12 (0.50) | mmol/l |
| L-LDL-P | Concentration of large LDL particles | Large LDL (average diameter 25.5 nm) | 209,144 (100) | 0.01 (0.01) | 6,820 (100) | 0.01 (0.01) | mmol/l |
| L-LDL-PL | Phospholipids in large LDL | Large LDL (average diameter 25.5 nm) | 209,144 (100) | 0.36 (0.09) | 6,820 (100) | 0.47 (0.11) | mmol/l |
| L-LDL-PL-pct | Phospholipids to total lipids ratio in large LDL | Large LDL ratios | 209,144 (100) | 22.47 (0.80) | 6,820 (100) | 22.03 (0.62) | % |
| L-LDL-TG | Triglycerides in large LDL | Large LDL (average diameter 25.5 nm) | 209,144 (100) | 0.10 (0.03) | 6,820 (100) | 0.14 (0.05) | mmol/l |
| L-LDL-TG-pct | Triglycerides to total lipids ratio in large LDL | Large LDL ratios | 209,144 (100) | 6.44 (1.86) | 6,820 (100) | 6.71 (2.39) | % |
| L-VLDL-C | Cholesterol in large VLDL | Large VLDL (average diameter 53.6 nm) | 209,144 (100) | 0.10 (0.05) | 6,820 (100) | 0.11 (0.05) | mmol/l |
| L-VLDL-C-pct | Cholesterol to total lipids ratio in large VLDL | Large VLDL ratios | 209,123 (99.9) | 30.20 (4.39) | 6,820 (100) | 33.54 (5.73) | % |
| L-VLDL-CE | Cholesteryl esters in large VLDL | Large VLDL (average diameter 53.6 nm) | 209,144 (100) | 0.05 (0.02) | 6,820 (100) | 0.06 (0.03) | mmol/l |
| L-VLDL-CE-pct | Cholesteryl esters to total lipids ratio in large VLDL | Large VLDL ratios | 209,123 (99.9) | 16.25 (3.50) | 6,820 (100) | 18.38 (4.26) | % |
| L-VLDL-FC | Free cholesterol in large VLDL | Large VLDL (average diameter 53.6 nm) | 209,144 (100) | 0.05 (0.03) | 6,820 (100) | 0.05 (0.03) | mmol/l |
| L-VLDL-FC-pct | Free cholesterol to total lipids ratio in large VLDL | Large VLDL ratios | 209,123 (99.9) | 13.95 (1.38) | 6,820 (100) | 14.93 (2.52) | % |
| L-VLDL-L | Total lipids in large VLDL | Large VLDL (average diameter 53.6 nm) | 209,144 (100) | 0.34 (0.18) | 6,820 (100) | 0.35 (0.19) | mmol/l |
| L-VLDL-P | Concentration of large VLDL particles | Large VLDL (average diameter 53.6 nm) | 209,144 (100) | 0.01 (0.01) | 6,820 (100) | 0.01 (0.01) | mmol/l |
| L-VLDL-PL | Phospholipids in large VLDL | Large VLDL (average diameter 53.6 nm) | 209,144 (100) | 0.07 (0.04) | 6,820 (100) | 0.08 (0.04) | mmol/l |
| L-VLDL-PL-pct | Phospholipids to total lipids ratio in large VLDL | Large VLDL ratios | 209,123 (99.9) | 19.13 (3.36) | 6,820 (100) | 20.86 (3.79) | % |
| L-VLDL-TG | Triglycerides in large VLDL | Large VLDL (average diameter 53.6 nm) | 209,144 (100) | 0.17 (0.10) | 6,820 (100) | 0.16 (0.10) | mmol/l |
| L-VLDL-TG-pct | Triglycerides to total lipids ratio in large VLDL | Large VLDL ratios | 209,123 (99.9) | 50.67 (5.92) | 6,820 (100) | 45.37 (7.82) | % |
| LA | Linoleic acid | Fatty acids | 208,978 (99.9) | 3.48 (0.70) | 6,794 (99.6) | 4.17 (0.80) | mmol/l |
| LA-pct | Ratio of linoleic acid to total fatty acids | Fatty acid ratios | 208,978 (99.9) | 28.65 (3.44) | 6,794 (99.6) | 29.84 (3.66) | % |
| Lactate | Lactate | Glycolysis related metabolites | 208,761 (99.8) | 3.94 (1.11) | 6,817 (99.9) | 4.45 (2.71) | mmol/l |
| LDL-C | LDL cholesterol | Cholesterol | 209,144 (100) | 1.76 (0.45) | 6,820 (100) | 2.30 (0.55) | mmol/l |
| LDL-CE | Cholesteryl esters in LDL | Cholesteryl esters | 209,144 (100) | 1.29 (0.33) | 6,820 (100) | 1.68 (0.40) | mmol/l |
| LDL-FC | Free cholesterol in LDL | Free cholesterol | 209,144 (100) | 0.47 (0.12) | 6,820 (100) | 0.62 (0.16) | mmol/l |
| LDL-L | Total lipids in LDL | Total lipids | 209,144 (100) | 2.53 (0.62) | 6,820 (100) | 3.30 (0.75) | mmol/l |
| LDL-P | Concentration of LDL particles | Lipoprotein particle concentrations | 209,144 (100) | 0.01 (0.01) | 6,820 (100) | 0.01 (0.01) | mmol/l |
| LDL-PL | Phospholipids in LDL | Phospholipids | 209,144 (100) | 0.61 (0.14) | 6,820 (100) | 0.79 (0.18) | mmol/l |
| LDL-size | Average diameter for LDL particles | Lipoprotein particle sizes | 209,144 (100) | 23.92 (0.09) | 6,820 (100) | 23.95 (0.11) | nm |
| LDL-TG | Triglycerides in LDL | Triglycerides | 209,144 (100) | 0.15 (0.04) | 6,820 (100) | 0.20 (0.07) | mmol/l |
| Leu | Leucine | Branched−chain amino acids | 209,122 (99.9) | 0.10 (0.03) | 6,820 (100) | 0.16 (0.04) | mmol/l |
| M-HDL-C | Cholesterol in medium HDL | Medium HDL (average diameter 10.9 nm) | 209,144 (100) | 0.50 (0.13) | 6,820 (100) | 0.51 (0.15) | mmol/l |
| M-HDL-C-pct | Cholesterol to total lipids ratio in medium HDL | Medium HDL ratios | 209,144 (100) | 47.48 (2.99) | 6,820 (100) | 47.21 (3.71) | % |
| M-HDL-CE | Cholesteryl esters in medium HDL | Medium HDL (average diameter 10.9 nm) | 209,144 (100) | 0.41 (0.10) | 6,820 (100) | 0.41 (0.12) | mmol/l |
| M-HDL-CE-pct | Cholesteryl esters to total lipids ratio in medium HDL | Medium HDL ratios | 209,144 (100) | 39.14 (2.59) | 6,820 (100) | 38.38 (3.53) | % |
| M-HDL-FC | Free cholesterol in medium HDL | Medium HDL (average diameter 10.9 nm) | 209,144 (100) | 0.09 (0.03) | 6,820 (100) | 0.09 (0.03) | mmol/l |
| M-HDL-FC-pct | Free cholesterol to total lipids ratio in medium HDL | Medium HDL ratios | 209,144 (100) | 8.34 (0.70) | 6,820 (100) | 8.83 (0.63) | % |
| M-HDL-L | Total lipids in medium HDL | Medium HDL (average diameter 10.9 nm) | 209,144 (100) | 1.05 (0.23) | 6,820 (100) | 1.06 (0.27) | mmol/l |
| M-HDL-P | Concentration of medium HDL particles | Medium HDL (average diameter 10.9 nm) | 209,144 (100) | 0.01 (0.01) | 6,820 (100) | 0.01 (0.01) | mmol/l |
| M-HDL-PL | Phospholipids in medium HDL | Medium HDL (average diameter 10.9 nm) | 209,144 (100) | 0.50 (0.10) | 6,820 (100) | 0.50 (0.12) | mmol/l |
| M-HDL-PL-pct | Phospholipids to total lipids ratio in medium HDL | Medium HDL ratios | 209,144 (100) | 47.12 (1.28) | 6,820 (100) | 46.69 (1.43) | % |
| M-HDL-TG | Triglycerides in medium HDL | Medium HDL (average diameter 10.9 nm) | 209,144 (100) | 0.06 (0.02) | 6,820 (100) | 0.06 (0.03) | mmol/l |
| M-HDL-TG-pct | Triglycerides to total lipids ratio in medium HDL | Medium HDL ratios | 209,144 (100) | 5.40 (1.83) | 6,820 (100) | 6.11 (2.66) | % |
| M-LDL-C | Cholesterol in medium LDL | Medium LDL (average diameter 23 nm) | 209,144 (100) | 0.43 (0.12) | 6,820 (100) | 0.56 (0.14) | mmol/l |
| M-LDL-C-pct | Cholesterol to total lipids ratio in medium LDL | Medium LDL ratios | 209,144 (100) | 68.34 (1.74) | 6,820 (100) | 68.04 (2.01) | % |
| M-LDL-CE | Cholesteryl esters in medium LDL | Medium LDL (average diameter 23 nm) | 209,144 (100) | 0.31 (0.09) | 6,820 (100) | 0.40 (0.10) | mmol/l |
| M-LDL-CE-pct | Cholesteryl esters to total lipids ratio in medium LDL | Medium LDL ratios | 209,144 (100) | 49.15 (1.94) | 6,820 (100) | 48.63 (1.95) | % |
| M-LDL-FC | Free cholesterol in medium LDL | Medium LDL (average diameter 23 nm) | 209,144 (100) | 0.12 (0.03) | 6,820 (100) | 0.16 (0.04) | mmol/l |
| M-LDL-FC-pct | Free cholesterol to total lipids ratio in medium LDL | Medium LDL ratios | 209,144 (100) | 19.19 (1.94) | 6,820 (100) | 19.40 (2.12) | % |
| M-LDL-L | Total lipids in medium LDL | Medium LDL (average diameter 23 nm) | 209,144 (100) | 0.63 (0.17) | 6,820 (100) | 0.81 (0.20) | mmol/l |
| M-LDL-P | Concentration of medium LDL particles | Medium LDL (average diameter 23 nm) | 209,144 (100) | 0.01 (0.01) | 6,820 (100) | 0.01 (0.01) | mmol/l |
| M-LDL-PL | Phospholipids in medium LDL | Medium LDL (average diameter 23 nm) | 209,144 (100) | 0.16 (0.04) | 6,820 (100) | 0.21 (0.05) | mmol/l |
| M-LDL-PL-pct | Phospholipids to total lipids ratio in medium LDL | Medium LDL ratios | 209,144 (100) | 26.13 (0.82) | 6,820 (100) | 26.25 (0.93) | % |
| M-LDL-TG | Triglycerides in medium LDL | Medium LDL (average diameter 23 nm) | 209,144 (100) | 0.03 (0.01) | 6,820 (100) | 0.05 (0.02) | mmol/l |
| M-LDL-TG-pct | Triglycerides to total lipids ratio in medium LDL | Medium LDL ratios | 209,144 (100) | 5.53 (1.61) | 6,820 (100) | 5.71 (2.16) | % |
| M-VLDL-C | Cholesterol in medium VLDL | Medium VLDL (average diameter 44.5 nm) | 209,144 (100) | 0.17 (0.07) | 6,820 (100) | 0.23 (0.08) | mmol/l |
| M-VLDL-C-pct | Cholesterol to total lipids ratio in medium VLDL | Medium VLDL ratios | 209,144 (100) | 29.75 (6.95) | 6,820 (100) | 33.61 (6.46) | % |
| M-VLDL-CE | Cholesteryl esters in medium VLDL | Medium VLDL (average diameter 44.5 nm) | 209,144 (100) | 0.09 (0.04) | 6,820 (100) | 0.13 (0.05) | mmol/l |
| M-VLDL-CE-pct | Cholesteryl esters to total lipids ratio in medium VLDL | Medium VLDL ratios | 209,144 (100) | 16.08 (5.20) | 6,820 (100) | 18.83 (4.87) | % |
| M-VLDL-FC | Free cholesterol in medium VLDL | Medium VLDL (average diameter 44.5 nm) | 209,144 (100) | 0.08 (0.03) | 6,820 (100) | 0.10 (0.03) | mmol/l |
| M-VLDL-FC-pct | Free cholesterol to total lipids ratio in medium VLDL | Medium VLDL ratios | 209,144 (100) | 13.67 (1.80) | 6,820 (100) | 14.78 (1.68) | % |
| M-VLDL-L | Total lipids in medium VLDL | Medium VLDL (average diameter 44.5 nm) | 209,144 (100) | 0.59 (0.21) | 6,820 (100) | 0.70 (0.23) | mmol/l |
| M-VLDL-P | Concentration of medium VLDL particles | Medium VLDL (average diameter 44.5 nm) | 209,144 (100) | 0.01 (0.01) | 6,820 (100) | 0.01 (0.01) | mmol/l |
| M-VLDL-PL | Phospholipids in medium VLDL | Medium VLDL (average diameter 44.5 nm) | 209,144 (100) | 0.13 (0.05) | 6,820 (100) | 0.17 (0.05) | mmol/l |
| M-VLDL-PL-pct | Phospholipids to total lipids ratio in medium VLDL | Medium VLDL ratios | 209,144 (100) | 22.21 (1.88) | 6,820 (100) | 23.95 (1.84) | % |
| M-VLDL-TG | Triglycerides in medium VLDL | Medium VLDL (average diameter 44.5 nm) | 209,144 (100) | 0.29 (0.12) | 6,820 (100) | 0.30 (0.13) | mmol/l |
| M-VLDL-TG-pct | Triglycerides to total lipids ratio in medium VLDL | Medium VLDL ratios | 209,144 (100) | 48.04 (8.71) | 6,820 (100) | 42.44 (8.14) | % |
| MUFA | Monounsaturated fatty acids | Fatty acids | 208,978 (99.9) | 2.95 (0.83) | 6,794 (99.6) | 3.36 (0.93) | mmol/l |
| MUFA-pct | Ratio of monounsaturated fatty acids to total fatty acids | Fatty acid ratios | 208,978 (99.9) | 23.82 (2.65) | 6,794 (99.6) | 23.59 (2.40) | % |
| non-HDL-C | Total cholesterol minus HDL-C | Cholesterol | 209,144 (100) | 3.34 (0.86) | 6,820 (100) | 4.33 (1.05) | mmol/l |
| Omega-3 | Omega-3 fatty acids | Fatty acids | 208,978 (99.9) | 0.55 (0.23) | 6,794 (99.6) | 0.63 (0.21) | mmol/l |
| Omega-3-pct | Ratio of omega-3 fatty acids to total fatty acids | Fatty acid ratios | 208,978 (99.9) | 4.51 (1.58) | 6,794 (99.6) | 4.46 (1.12) | % |
| Omega-6 | Omega-6 fatty acids | Fatty acids | 208,978 (99.9) | 4.54 (0.70) | 6,794 (99.6) | 5.18 (0.79) | mmol/l |
| Omega-6/Omega-3 | Ratio of omega-6 fatty acids to omega-3 fatty acids | Fatty acid ratios | 208,969 (99.9) | 9.45 (4.21) | 6,794 (99.6) | 8.90 (2.59) | ratio |
| Omega-6-pct | Ratio of omega-6 fatty acids to total fatty acids | Fatty acid ratios | 208,978 (99.9) | 37.58 (3.57) | 6,794 (99.6) | 37.21 (3.55) | % |
| Phe | Phenylalanine | Aromatic amino acids | 209,046 (99.9) | 0.05 (0.01) | 6,786 (99.5) | 0.10 (0.02) | mmol/l |
| Phosphatidylc | Phosphatidylcholines | Other lipids | 208,978 (99.9) | 2.13 (0.39) | 6,794 (99.6) | 2.38 (0.43) | mmol/l |
| Phosphoglyc | Phosphoglycerides | Other lipids | 208,978 (99.9) | 2.32 (0.41) | 6,794 (99.6) | 2.61 (0.45) | mmol/l |
| PUFA | Polyunsaturated fatty acids | Fatty acids | 208,978 (99.9) | 5.10 (0.82) | 6,794 (99.6) | 5.81 (0.92) | mmol/l |
| PUFA/MUFA | Ratio of polyunsaturated fatty acids to monounsaturated fatty acids | Fatty acid ratios | 208,978 (99.9) | 1.80 (0.34) | 6,794 (99.6) | 1.80 (0.31) | ratio |
| PUFA-pct | Ratio of polyunsaturated fatty acids to total fatty acids | Fatty acid ratios | 208,978 (99.9) | 42.10 (3.75) | 6,794 (99.6) | 41.67 (3.57) | % |
| Pyruvate | Pyruvate | Glycolysis related metabolites | 208,489 (99.7) | 0.08 (0.03) | 6,698 (98.2) | 0.20 (0.24) | mmol/l |
| Remnant-C | Remnant cholesterol (non-HDL, non-LDL -cholesterol) | Cholesterol | 209,144 (100) | 1.58 (0.43) | 6,820 (100) | 2.03 (0.52) | mmol/l |
| S-HDL-C | Cholesterol in small HDL | Small HDL (average diameter 8.7 nm) | 209,144 (100) | 0.45 (0.06) | 6,820 (100) | 0.47 (0.08) | mmol/l |
| S-HDL-C-pct | Cholesterol to total lipids ratio in small HDL | Small HDL ratios | 209,144 (100) | 38.31 (1.88) | 6,820 (100) | 39.11 (2.34) | % |
| S-HDL-CE | Cholesteryl esters in small HDL | Small HDL (average diameter 8.7 nm) | 209,144 (100) | 0.33 (0.05) | 6,820 (100) | 0.34 (0.06) | mmol/l |
| S-HDL-CE-pct | Cholesteryl esters to total lipids ratio in small HDL | Small HDL ratios | 209,144 (100) | 28.36 (1.87) | 6,820 (100) | 28.29 (2.54) | % |
| S-HDL-FC | Free cholesterol in small HDL | Small HDL (average diameter 8.7 nm) | 209,144 (100) | 0.12 (0.02) | 6,820 (100) | 0.13 (0.02) | mmol/l |
| S-HDL-FC-pct | Free cholesterol to total lipids ratio in small HDL | Small HDL ratios | 209,144 (100) | 9.95 (0.54) | 6,820 (100) | 10.82 (0.76) | % |
| S-HDL-L | Total lipids in small HDL | Small HDL (average diameter 8.7 nm) | 209,144 (100) | 1.18 (0.16) | 6,820 (100) | 1.20 (0.19) | mmol/l |
| S-HDL-P | Concentration of small HDL particles | Small HDL (average diameter 8.7 nm) | 209,144 (100) | 0.01 (0.00) | 6,820 (100) | 0.01 (0.00) | mmol/l |
| S-HDL-PL | Phospholipids in small HDL | Small HDL (average diameter 8.7 nm) | 209,144 (100) | 0.67 (0.10) | 6,820 (100) | 0.67 (0.12) | mmol/l |
| S-HDL-PL-pct | Phospholipids to total lipids ratio in small HDL | Small HDL ratios | 209,144 (100) | 57.09 (1.22) | 6,820 (100) | 55.74 (1.46) | % |
| S-HDL-TG | Triglycerides in small HDL | Small HDL (average diameter 8.7 nm) | 209,144 (100) | 0.05 (0.02) | 6,820 (100) | 0.06 (0.02) | mmol/l |
| S-HDL-TG-pct | Triglycerides to total lipids ratio in small HDL | Small HDL ratios | 209,144 (100) | 4.60 (1.33) | 6,820 (100) | 5.15 (1.89) | % |
| S-LDL-C | Cholesterol in small LDL | Small LDL (average diameter 18.7 nm) | 209,144 (100) | 0.18 (0.05) | 6,820 (100) | 0.23 (0.05) | mmol/l |
| S-LDL-C-pct | Cholesterol to total lipids ratio in small LDL | Small LDL ratios | 209,144 (100) | 63.18 (2.04) | 6,820 (100) | 63.65 (2.00) | % |
| S-LDL-CE | Cholesteryl esters in small LDL | Small LDL (average diameter 18.7 nm) | 209,144 (100) | 0.13 (0.03) | 6,820 (100) | 0.17 (0.04) | mmol/l |
| S-LDL-CE-pct | Cholesteryl esters to total lipids ratio in small LDL | Small LDL ratios | 209,144 (100) | 45.90 (2.07) | 6,820 (100) | 46.28 (1.91) | % |
| S-LDL-FC | Free cholesterol in small LDL | Small LDL (average diameter 18.7 nm) | 209,144 (100) | 0.05 (0.01) | 6,820 (100) | 0.06 (0.02) | mmol/l |
| S-LDL-FC-pct | Free cholesterol to total lipids ratio in small LDL | Small LDL ratios | 209,144 (100) | 17.28 (2.01) | 6,820 (100) | 17.37 (2.44) | % |
| S-LDL-L | Total lipids in small LDL | Small LDL (average diameter 18.7 nm) | 209,144 (100) | 0.29 (0.07) | 6,820 (100) | 0.36 (0.08) | mmol/l |
| S-LDL-P | Concentration of small LDL particles | Small LDL (average diameter 18.7 nm) | 209,144 (100) | 0.01 (0.01) | 6,820 (100) | 0.01 (0.01) | mmol/l |
| S-LDL-PL | Phospholipids in small LDL | Small LDL (average diameter 18.7 nm) | 209,144 (100) | 0.09 (0.02) | 6,820 (100) | 0.11 (0.02) | mmol/l |
| S-LDL-PL-pct | Phospholipids to total lipids ratio in small LDL | Small LDL ratios | 209,144 (100) | 31.23 (1.75) | 6,820 (100) | 30.95 (1.55) | % |
| S-LDL-TG | Triglycerides in small LDL | Small LDL (average diameter 18.7 nm) | 209,144 (100) | 0.02 (0.01) | 6,820 (100) | 0.02 (0.01) | mmol/l |
| S-LDL-TG-pct | Triglycerides to total lipids ratio in small LDL | Small LDL ratios | 209,144 (100) | 5.59 (1.84) | 6,820 (100) | 5.29 (2.33) | % |
| S-VLDL-C | Cholesterol in small VLDL | Small VLDL (average diameter 36.8 nm) | 209,144 (100) | 0.16 (0.05) | 6,820 (100) | 0.21 (0.07) | mmol/l |
| S-VLDL-C-pct | Cholesterol to total lipids ratio in small VLDL | Small VLDL ratios | 209,144 (100) | 37.79 (4.78) | 6,820 (100) | 41.02 (4.64) | % |
| S-VLDL-CE | Cholesteryl esters in small VLDL | Small VLDL (average diameter 36.8 nm) | 209,144 (100) | 0.10 (0.03) | 6,820 (100) | 0.13 (0.04) | mmol/l |
| S-VLDL-CE-pct | Cholesteryl esters to total lipids ratio in small VLDL | Small VLDL ratios | 209,144 (100) | 23.55 (2.82) | 6,820 (100) | 25.73 (2.67) | % |
| S-VLDL-FC | Free cholesterol in small VLDL | Small VLDL (average diameter 36.8 nm) | 209,144 (100) | 0.06 (0.02) | 6,820 (100) | 0.08 (0.02) | mmol/l |
| S-VLDL-FC-pct | Free cholesterol to total lipids ratio in small VLDL | Small VLDL ratios | 209,144 (100) | 14.24 (2.26) | 6,820 (100) | 15.29 (2.29) | % |
| S-VLDL-L | Total lipids in small VLDL | Small VLDL (average diameter 36.8 nm) | 209,144 (100) | 0.42 (0.13) | 6,820 (100) | 0.52 (0.17) | mmol/l |
| S-VLDL-P | Concentration of small VLDL particles | Small VLDL (average diameter 36.8 nm) | 209,144 (100) | 0.01 (0.01) | 6,820 (100) | 0.01 (0.01) | mmol/l |
| S-VLDL-PL | Phospholipids in small VLDL | Small VLDL (average diameter 36.8 nm) | 209,144 (100) | 0.10 (0.03) | 6,820 (100) | 0.13 (0.04) | mmol/l |
| S-VLDL-PL-pct | Phospholipids to total lipids ratio in small VLDL | Small VLDL ratios | 209,144 (100) | 23.60 (2.11) | 6,820 (100) | 24.63 (2.27) | % |
| S-VLDL-TG | Triglycerides in small VLDL | Small VLDL (average diameter 36.8 nm) | 209,144 (100) | 0.16 (0.06) | 6,820 (100) | 0.18 (0.08) | mmol/l |
| S-VLDL-TG-pct | Triglycerides to total lipids ratio in small VLDL | Small VLDL ratios | 209,144 (100) | 38.61 (6.78) | 6,820 (100) | 34.35 (6.77) | % |
| SFA | Saturated fatty acids | Fatty acids | 208,978 (99.9) | 4.19 (0.97) | 6,794 (99.6) | 4.91 (1.12) | mmol/l |
| SFA-pct | Ratio of saturated fatty acids to total fatty acids | Fatty acid ratios | 208,978 (99.9) | 34.08 (1.96) | 6,794 (99.6) | 34.74 (1.86) | % |
| Sphingomyelins | Sphingomyelins | Other lipids | 208,973 (99.9) | 0.46 (0.08) | 6,794 (99.6) | 0.54 (0.08) | mmol/l |
| TG/PG | Ratio of triglycerides to phosphoglycerides | Other lipids | 208,978 (99.9) | 0.58 (0.23) | 6,794 (99.6) | 0.55 (0.22) | ratio |
| Total-BCAA | Total concentration of branched-chain amino acids (leucine + isoleucine + valine) | Branched−chain amino acids | 208,977 (99.9) | 0.37 (0.09) | 6,794 (99.6) | 0.49 (0.10) | mmol/l |
| Total-C | Total cholesterol | Cholesterol | 209,144 (100) | 4.67 (0.98) | 6,820 (100) | 5.72 (1.12) | mmol/l |
| Total-CE | Total esterified cholesterol | Cholesteryl esters | 209,144 (100) | 3.40 (0.70) | 6,820 (100) | 4.14 (0.81) | mmol/l |
| Total-FA | Total fatty acids | Fatty acids | 208,978 (99.9) | 12.23 (2.42) | 6,794 (99.6) | 14.08 (2.76) | mmol/l |
| Total-FC | Total free cholesterol | Free cholesterol | 209,144 (100) | 1.28 (0.28) | 6,820 (100) | 1.58 (0.32) | mmol/l |
| Total-L | Total lipids in lipoprotein particles | Total lipids | 209,144 (100) | 8.99 (1.69) | 6,820 (100) | 10.53 (1.90) | mmol/l |
| Total-P | Total concentration of lipoprotein particles | Lipoprotein particle concentrations | 209,144 (100) | 0.02 (0.00) | 6,820 (100) | 0.02 (0.00) | mmol/l |
| Total-PL | Total phospholipids in lipoprotein particles | Phospholipids | 209,144 (100) | 2.97 (0.49) | 6,820 (100) | 3.36 (0.54) | mmol/l |
| Total-TG | Total triglycerides | Triglycerides | 209,144 (100) | 1.35 (0.59) | 6,820 (100) | 1.45 (0.66) | mmol/l |
| Tyr | Tyrosine | Aromatic amino acids | 208,902 (99.8) | 0.06 (0.01) | 6,781 (99.4) | 0.07 (0.02) | mmol/l |
| Unsaturation | Degree of unsaturation | Fatty acids | 208,978 (99.9) | 1.36 (0.08) | 6,794 (99.6) | 1.35 (0.07) | degree |
| Val | Valine | Branched−chain amino acids | 208,982 (99.9) | 0.21 (0.04) | 6,793 (99.6) | 0.26 (0.05) | mmol/l |
| VLDL-C | VLDL cholesterol | Cholesterol | 209,144 (100) | 0.73 (0.25) | 6,820 (100) | 0.92 (0.30) | mmol/l |
| VLDL-CE | Cholesteryl esters in VLDL | Cholesteryl esters | 209,144 (100) | 0.44 (0.15) | 6,820 (100) | 0.56 (0.17) | mmol/l |
| VLDL-FC | Free cholesterol in VLDL | Free cholesterol | 209,144 (100) | 0.30 (0.11) | 6,820 (100) | 0.36 (0.12) | mmol/l |
| VLDL-L | Total lipids in VLDL | Total lipids | 209,144 (100) | 2.16 (0.87) | 6,820 (100) | 2.44 (0.96) | mmol/l |
| VLDL-P | Concentration of VLDL particles | Lipoprotein particle concentrations | 209,144 (100) | 0.01 (0.01) | 6,820 (100) | 0.01 (0.01) | mmol/l |
| VLDL-PL | Phospholipids in VLDL | Phospholipids | 209,144 (100) | 0.48 (0.18) | 6,820 (100) | 0.58 (0.21) | mmol/l |
| VLDL-size | Average diameter for VLDL particles | Lipoprotein particle sizes | 209,144 (100) | 38.66 (1.24) | 6,820 (100) | 38.14 (1.04) | nm |
| VLDL-TG | Triglycerides in VLDL | Triglycerides | 209,144 (100) | 0.95 (0.49) | 6,820 (100) | 0.94 (0.52) | mmol/l |
| XL-HDL-C | Cholesterol in very large HDL | Very large HDL (average diameter 14.3 nm) | 209,144 (100) | 0.08 (0.04) | 6,820 (100) | 0.09 (0.03) | mmol/l |
| XL-HDL-C-pct | Cholesterol to total lipids ratio in very large HDL | Very large HDL ratios | 209,054 (99.9) | 50.07 (4.96) | 6,820 (100) | 50.60 (4.92) | % |
| XL-HDL-CE | Cholesteryl esters in very large HDL | Very large HDL (average diameter 14.3 nm) | 209,144 (100) | 0.06 (0.03) | 6,820 (100) | 0.07 (0.02) | mmol/l |
| XL-HDL-CE-pct | Cholesteryl esters to total lipids ratio in very large HDL | Very large HDL ratios | 209,054 (99.9) | 34.90 (3.54) | 6,820 (100) | 35.78 (3.87) | % |
| XL-HDL-FC | Free cholesterol in very large HDL | Very large HDL (average diameter 14.3 nm) | 209,144 (100) | 0.02 (0.01) | 6,820 (100) | 0.03 (0.01) | mmol/l |
| XL-HDL-FC-pct | Free cholesterol to total lipids ratio in very large HDL | Very large HDL ratios | 209,054 (99.9) | 15.17 (3.68) | 6,820 (100) | 14.71 (2.82) | % |
| XL-HDL-L | Total lipids in very large HDL | Very large HDL (average diameter 14.3 nm) | 209,144 (100) | 0.17 (0.08) | 6,820 (100) | 0.18 (0.07) | mmol/l |
| XL-HDL-P | Concentration of very large HDL particles | Very large HDL (average diameter 14.3 nm) | 209,144 (100) | 0.01 (0.01) | 6,820 (100) | 0.01 (0.01) | mmol/l |
| XL-HDL-PL | Phospholipids in very large HDL | Very large HDL (average diameter 14.3 nm) | 209,144 (100) | 0.08 (0.05) | 6,820 (100) | 0.08 (0.04) | mmol/l |
| XL-HDL-PL-pct | Phospholipids to total lipids ratio in very large HDL | Very large HDL ratios | 209,054 (99.9) | 44.51 (6.75) | 6,820 (100) | 43.17 (6.25) | % |
| XL-HDL-TG | Triglycerides in very large HDL | Very large HDL (average diameter 14.3 nm) | 209,144 (100) | 0.01 (0.00) | 6,820 (100) | 0.01 (0.01) | mmol/l |
| XL-HDL-TG-pct | Triglycerides to total lipids ratio in very large HDL | Very large HDL ratios | 209,054 (99.9) | 5.41 (4.00) | 6,820 (100) | 3.93 (4.32) | % |
| XL-VLDL-C | Cholesterol in very large VLDL | Very large VLDL (average diameter 64 nm) | 209,144 (100) | 0.05 (0.03) | 6,820 (100) | 0.06 (0.03) | mmol/l |
| XL-VLDL-C-pct | Cholesterol to total lipids ratio in very large VLDL | Very large VLDL ratios | 207,296 (99.1) | 29.26 (8.08) | 6,820 (100) | 34.73 (11.19) | % |
| XL-VLDL-CE | Cholesteryl esters in very large VLDL | Very large VLDL (average diameter 64 nm) | 209,144 (100) | 0.03 (0.01) | 6,820 (100) | 0.04 (0.02) | mmol/l |
| XL-VLDL-CE-pct | Cholesteryl esters to total lipids ratio in very large VLDL | Very large VLDL ratios | 207,296 (99.1) | 17.28 (6.78) | 6,820 (100) | 22.03 (9.86) | % |
| XL-VLDL-FC | Free cholesterol in very large VLDL | Very large VLDL (average diameter 64 nm) | 209,144 (100) | 0.02 (0.01) | 6,820 (100) | 0.02 (0.02) | mmol/l |
| XL-VLDL-FC-pct | Free cholesterol to total lipids ratio in very large VLDL | Very large VLDL ratios | 207,296 (99.1) | 11.98 (1.73) | 6,820 (100) | 10.65 (4.62) | % |
| XL-VLDL-L | Total lipids in very large VLDL | Very large VLDL (average diameter 64 nm) | 209,144 (100) | 0.21 (0.14) | 6,820 (100) | 0.21 (0.14) | mmol/l |
| XL-VLDL-P | Concentration of very large VLDL particles | Very large VLDL (average diameter 64 nm) | 209,144 (100) | 0.01 (0.01) | 6,820 (100) | 0.01 (0.01) | mmol/l |
| XL-VLDL-PL | Phospholipids in very large VLDL | Very large VLDL (average diameter 64 nm) | 209,144 (100) | 0.04 (0.03) | 6,820 (100) | 0.04 (0.03) | mmol/l |
| XL-VLDL-PL-pct | Phospholipids to total lipids ratio in very large VLDL | Very large VLDL ratios | 207,296 (99.1) | 18.33 (2.67) | 6,820 (100) | 17.56 (5.96) | % |
| XL-VLDL-TG | Triglycerides in very large VLDL | Very large VLDL (average diameter 64 nm) | 209,144 (100) | 0.12 (0.08) | 6,820 (100) | 0.11 (0.09) | mmol/l |
| XL-VLDL-TG-pct | Triglycerides to total lipids ratio in very large VLDL | Very large VLDL ratios | 207,296 (99.1) | 52.42 (8.15) | 6,820 (100) | 45.18 (13.12) | % |
| XS-VLDL-C | Cholesterol in very small VLDL | Very small VLDL (average diameter 31.3 nm) | 209,144 (100) | 0.19 (0.05) | 6,820 (100) | 0.24 (0.07) | mmol/l |
| XS-VLDL-C-pct | Cholesterol to total lipids ratio in very small VLDL | Very small VLDL ratios | 209,144 (100) | 50.96 (4.81) | 6,820 (100) | 51.68 (4.73) | % |
| XS-VLDL-CE | Cholesteryl esters in very small VLDL | Very small VLDL (average diameter 31.3 nm) | 209,144 (100) | 0.13 (0.04) | 6,820 (100) | 0.17 (0.05) | mmol/l |
| XS-VLDL-CE-pct | Cholesteryl esters to total lipids ratio in very small VLDL | Very small VLDL ratios | 209,144 (100) | 34.93 (4.34) | 6,820 (100) | 35.73 (4.36) | % |
| XS-VLDL-FC | Free cholesterol in very small VLDL | Very small VLDL (average diameter 31.3 nm) | 209,144 (100) | 0.06 (0.02) | 6,820 (100) | 0.08 (0.02) | mmol/l |
| XS-VLDL-FC-pct | Free cholesterol to total lipids ratio in very small VLDL | Very small VLDL ratios | 209,144 (100) | 16.02 (0.62) | 6,820 (100) | 15.95 (0.57) | % |
| XS-VLDL-L | Total lipids in very small VLDL | Very small VLDL (average diameter 31.3 nm) | 209,144 (100) | 0.37 (0.09) | 6,820 (100) | 0.48 (0.13) | mmol/l |
| XS-VLDL-P | Concentration of very small VLDL particles | Very small VLDL (average diameter 31.3 nm) | 209,144 (100) | 0.01 (0.01) | 6,820 (100) | 0.01 (0.01) | mmol/l |
| XS-VLDL-PL | Phospholipids in very small VLDL | Very small VLDL (average diameter 31.3 nm) | 209,144 (100) | 0.11 (0.03) | 6,820 (100) | 0.14 (0.04) | mmol/l |
| XS-VLDL-PL-pct | Phospholipids to total lipids ratio in very small VLDL | Very small VLDL ratios | 209,144 (100) | 29.25 (0.92) | 6,820 (100) | 29.28 (1.13) | % |
| XS-VLDL-TG | Triglycerides in very small VLDL | Very small VLDL (average diameter 31.3 nm) | 209,144 (100) | 0.07 (0.02) | 6,820 (100) | 0.09 (0.03) | mmol/l |
| XS-VLDL-TG-pct | Triglycerides to total lipids ratio in very small VLDL | Very small VLDL ratios | 209,144 (100) | 19.79 (4.28) | 6,820 (100) | 19.04 (4.13) | % |
| XXL-VLDL-C | Cholesterol in chylomicrons and extremely large VLDL | Chylomicrons and extremely large VLDL (particle diameters from 75 nm upwards) | 209,144 (100) | 0.06 (0.04) | 6,820 (100) | 0.05 (0.05) | mmol/l |
| XXL-VLDL-C-pct | Cholesterol to total lipids ratio in chylomicrons and extremely large VLDL | Chylomicrons and extremely large VLDL ratios | 200,675 (95.9) | 29.75 (12.66) | 6,820 (100) | 35.97 (26.27) | % |
| XXL-VLDL-CE | Cholesteryl esters in chylomicrons and extremely large VLDL | Chylomicrons and extremely large VLDL (particle diameters from 75 nm upwards) | 209,144 (100) | 0.03 (0.02) | 6,820 (100) | 0.03 (0.03) | mmol/l |
| XXL-VLDL-CE-pct | Cholesteryl esters to total lipids ratio in chylomicrons and extremely large VLDL | Chylomicrons and extremely large VLDL ratios | 200,675 (95.9) | 16.88 (9.32) | 6,820 (100) | 22.14 (20.09) | % |
| XXL-VLDL-FC | Free cholesterol in chylomicrons and extremely large VLDL | Chylomicrons and extremely large VLDL (particle diameters from 75 nm upwards) | 209,144 (100) | 0.02 (0.02) | 6,820 (100) | 0.02 (0.02) | mmol/l |
| XXL-VLDL-FC-pct | Free cholesterol to total lipids ratio in chylomicrons and extremely large VLDL | Chylomicrons and extremely large VLDL ratios | 200,675 (95.9) | 12.87 (4.53) | 6,820 (100) | 7.96 (6.61) | % |
| XXL-VLDL-L | Total lipids in chylomicrons and extremely large VLDL | Chylomicrons and extremely large VLDL (particle diameters from 75 nm upwards) | 209,144 (100) | 0.23 (0.20) | 6,820 (100) | 0.18 (0.21) | mmol/l |
| XXL-VLDL-P | Concentration of chylomicrons and extremely large VLDL particles | Chylomicrons and extremely large VLDL (particle diameters from 75 nm upwards) | 209,144 (100) | 0.01 (0.01) | 6,820 (100) | 0.01 (0.01) | mmol/l |
| XXL-VLDL-PL | Phospholipids in chylomicrons and extremely large VLDL | Chylomicrons and extremely large VLDL (particle diameters from 75 nm upwards) | 209,144 (100) | 0.04 (0.03) | 6,820 (100) | 0.03 (0.04) | mmol/l |
| XXL-VLDL-PL-pct | Phospholipids to total lipids ratio in chylomicrons and extremely large VLDL | Chylomicrons and extremely large VLDL ratios | 200,675 (95.9) | 15.39 (4.24) | 6,820 (100) | 11.04 (8.67) | % |
| XXL-VLDL-TG | Triglycerides in chylomicrons and extremely large VLDL | Chylomicrons and extremely large VLDL (particle diameters from 75 nm upwards) | 209,144 (100) | 0.14 (0.13) | 6,820 (100) | 0.10 (0.13) | mmol/l |
| XXL-VLDL-TG-pct | Triglycerides to total lipids ratio in chylomicrons and extremely large VLDL | Chylomicrons and extremely large VLDL ratios | 200,675 (95.9) | 54.86 (13.84) | 6,820 (100) | 49.07 (16.50) | % |

Abbreviations: HDL, high-density lipoprotein; IDL, intermediate-density lipoprotein; LDL, low-density lipoprotein; VLDL, very-low-density lipoprotein; SD, standard deviation.


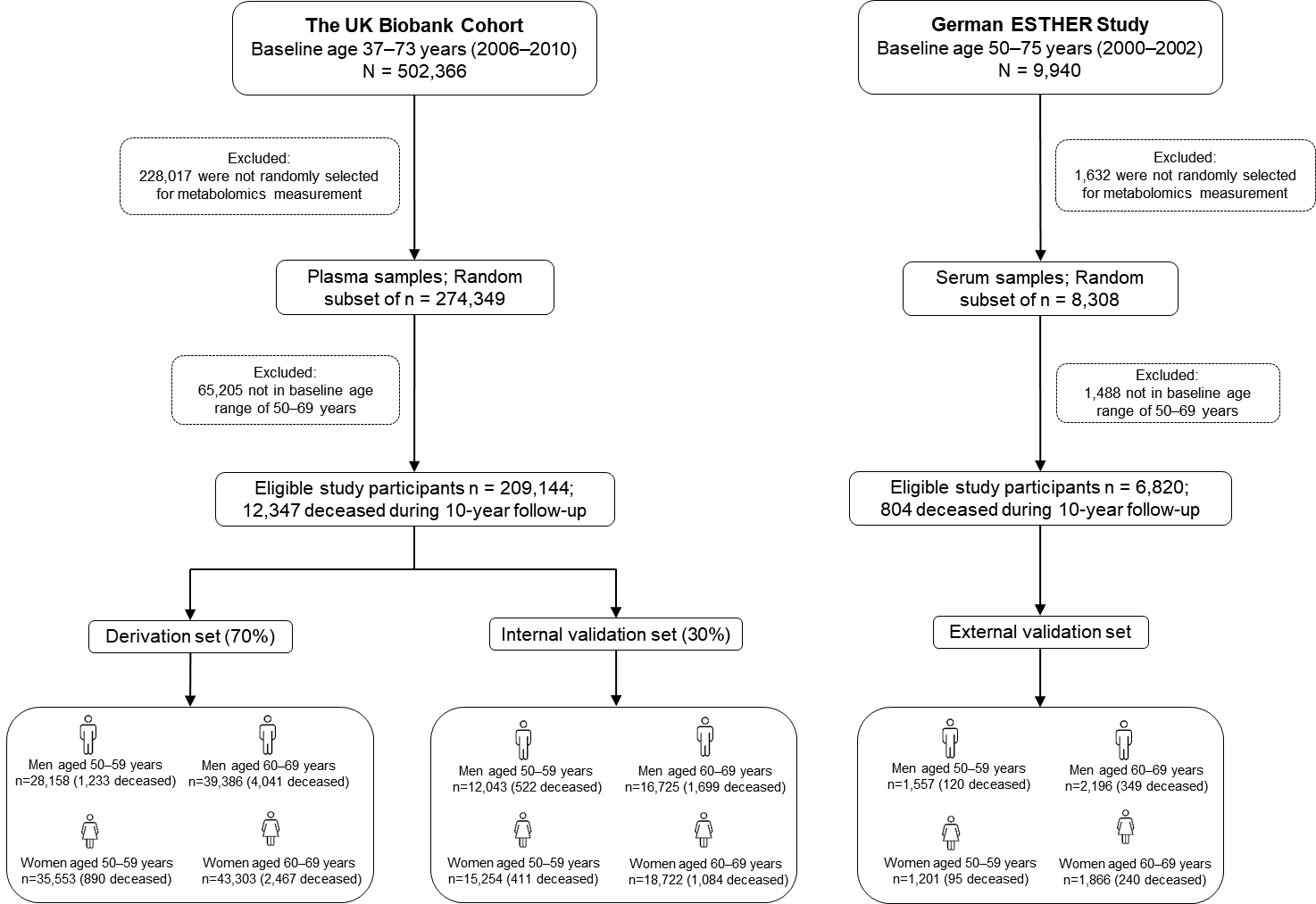


**Figure S1. Flow chart of study population from the UK Biobank and ESTHER study.**


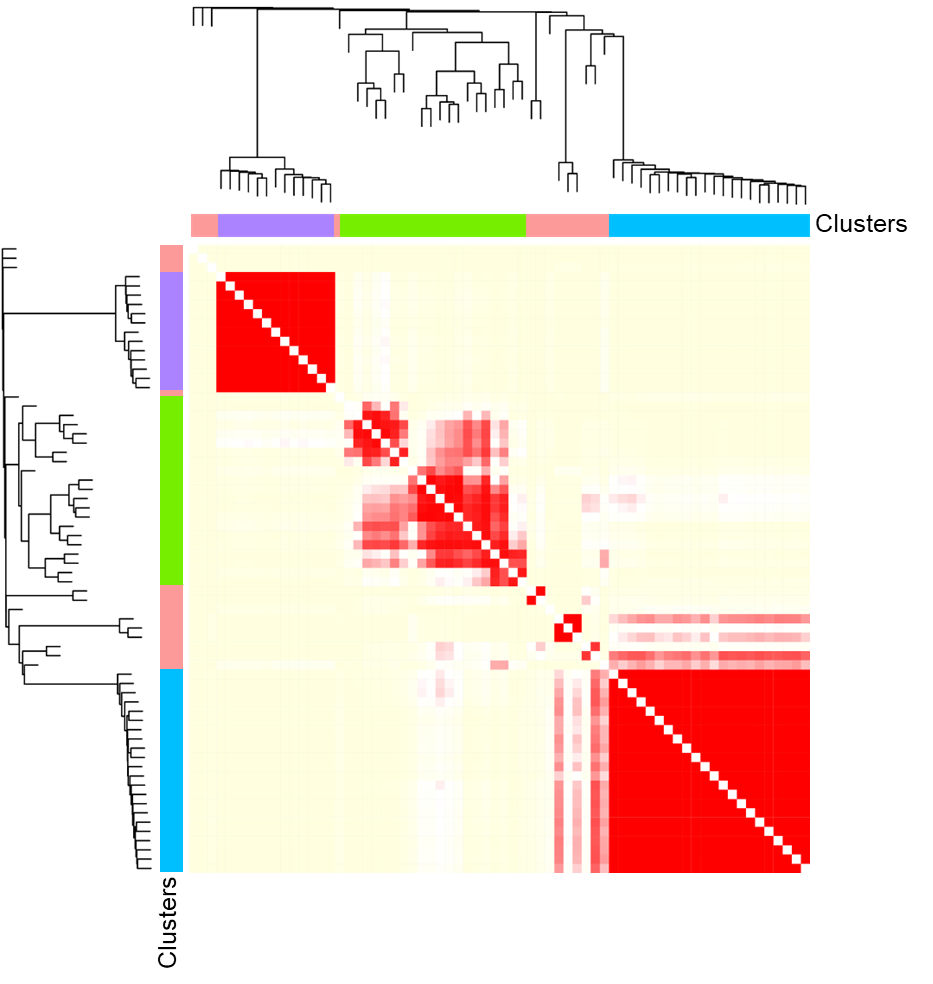


**Figure S2.** **Topological overlap map of 68 metabolomic biomarkers.**

Notes: Metabolic biomarkers are arranged in rows and columns based on the clustering tree. Light yellow shades indicate low topological overlap (low similarity), while red shades indicate higher overlap and similarity.^[11]^ Metabolite clusters are represented by the squares along the diagonal.


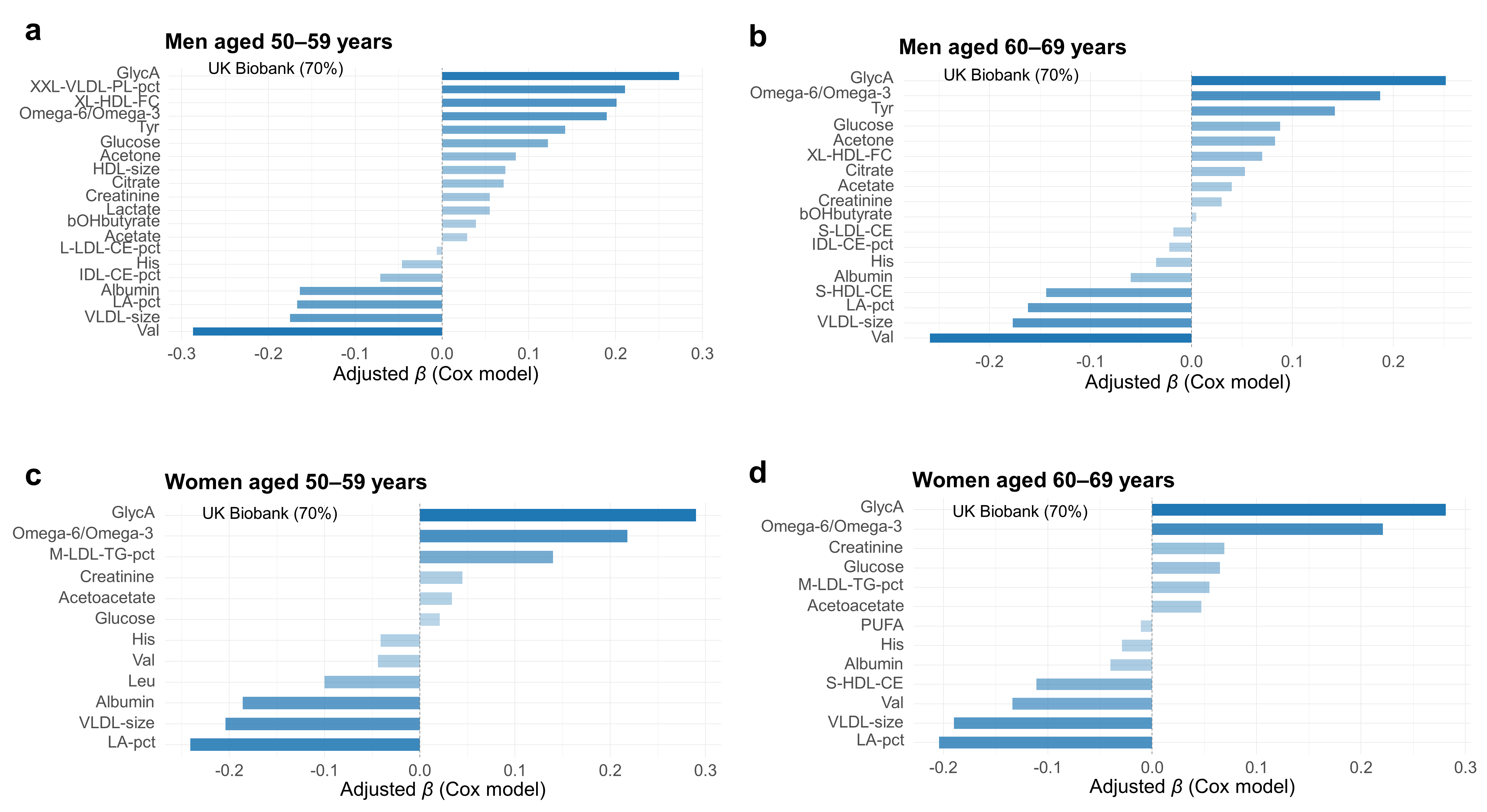


**Figure S3.** **Effect sizes of standardized *β*-coefficients of selected biomarkers used to derive mortality risk scores in the training set (70% of the UK Biobank).**

Note: Biomarkers are ranked by their effect sizes. Biomarker names are abbreviated in the figure; full names are listed in **Table S10** (Supporting Information).


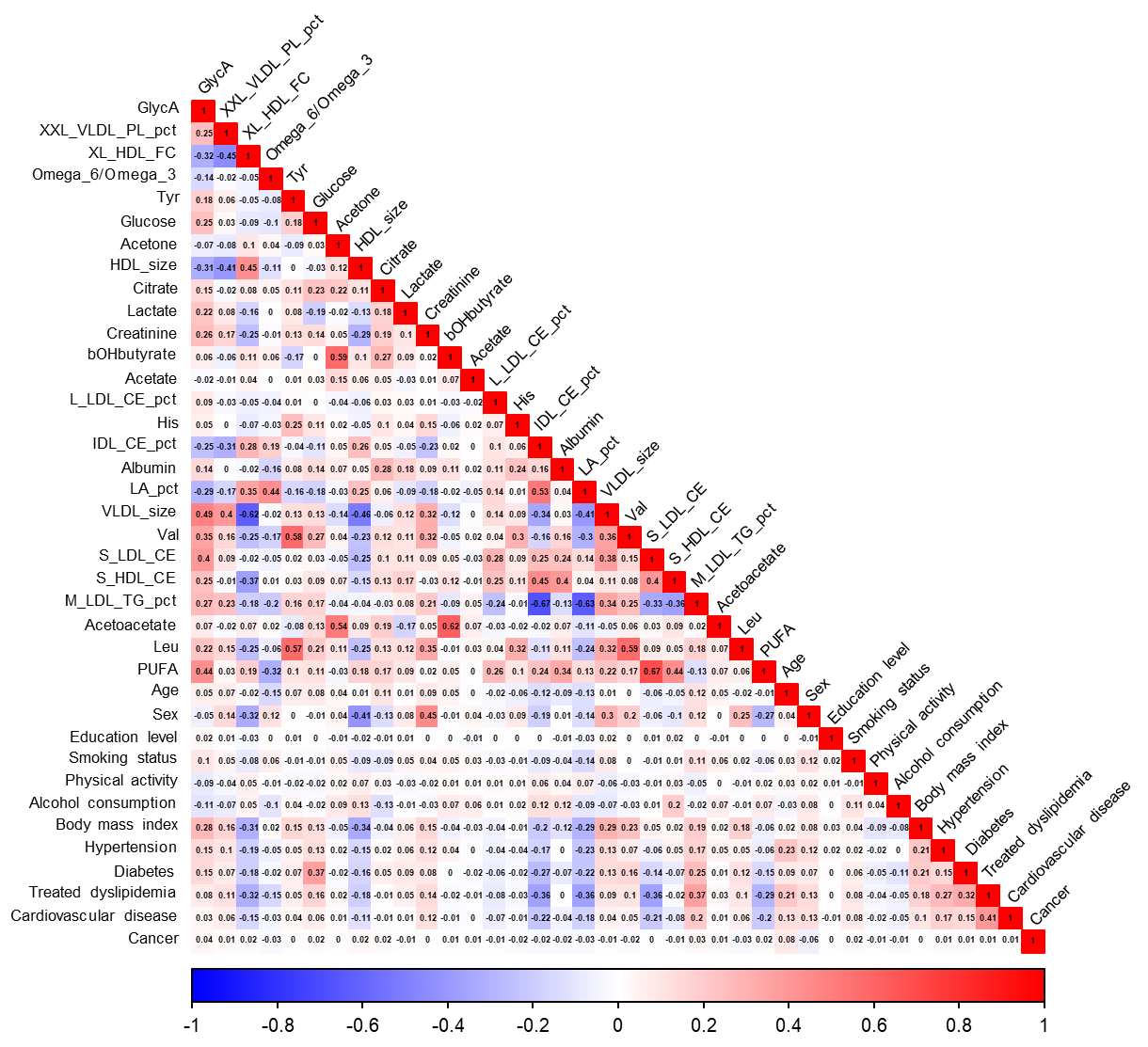


**Figure S4.** **Spearman correlations**^[6]^ **between the conventional risk factors and 26 metabolomic biomarkers in the derivation set (70% UK Biobank).**


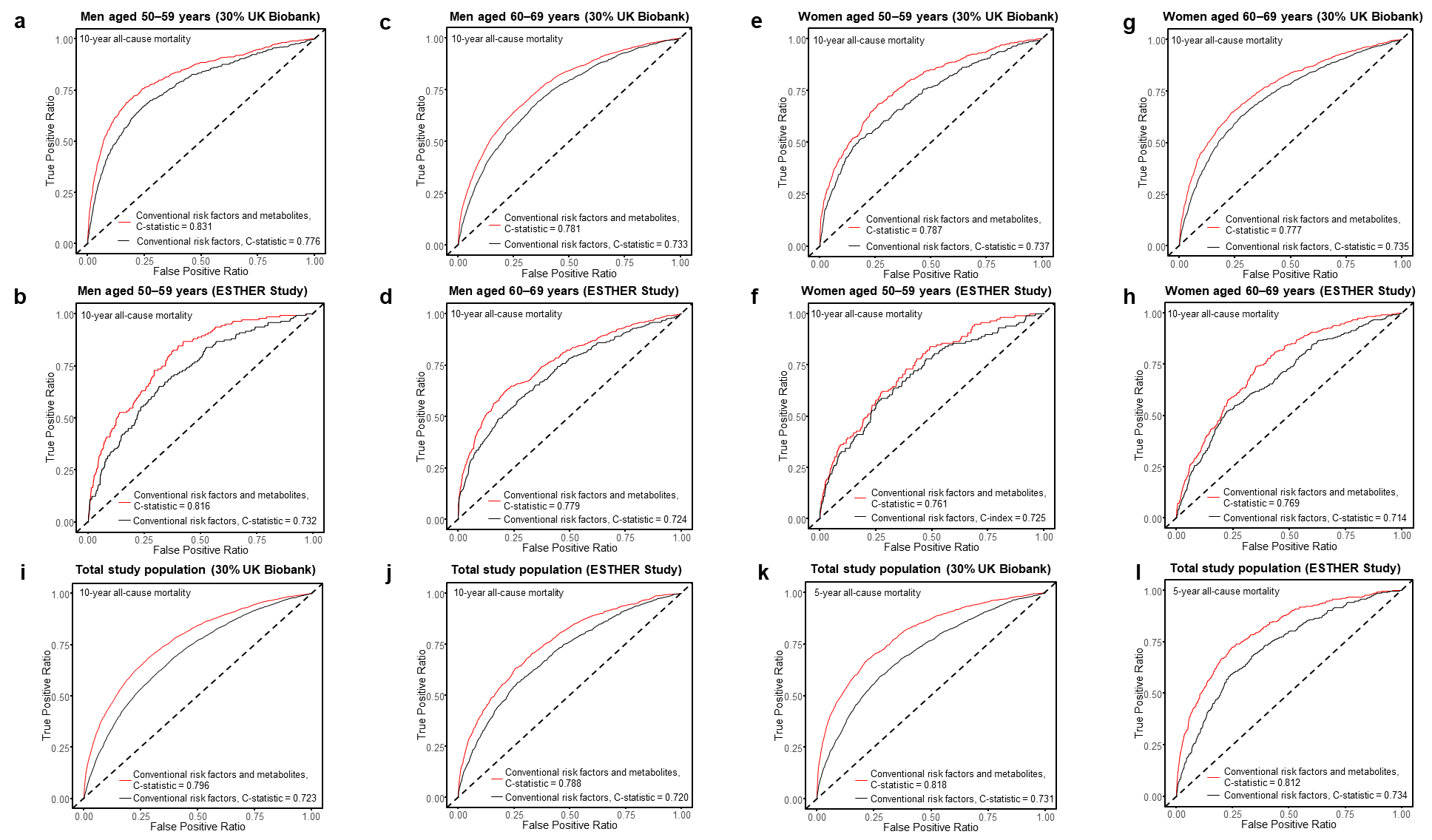


**Figure S5. Improved mortality prediction accuracy with identified metabolomic biomarkers in sex- and age-specific groups, as well as the total study population.**

Notes: For 10-year all-cause mortality, ROC curves^[12]^ were assessed in: **a)** younger men (30% UK Biobank), **b)** younger men (ESTHER), **c)** older men (30% UK Biobank), **d)** older men (ESTHER), **e)** younger women (30% UK Biobank), **f)** younger women (ESTHER), **g)** older women (30% UK Biobank), **h)** older women (ESTHER), **i)** the total study population (30% UK Biobank), and **j)** the total study population (ESTHER). For 5-year all-cause mortality, ROC curves were assessed in **k)** the total study population from 30% of UK Biobank and **l)** the total study population from ESTHER. The curves are based on the predictions from the conventional risk factors (black) and the metabolic biomarkers (red).


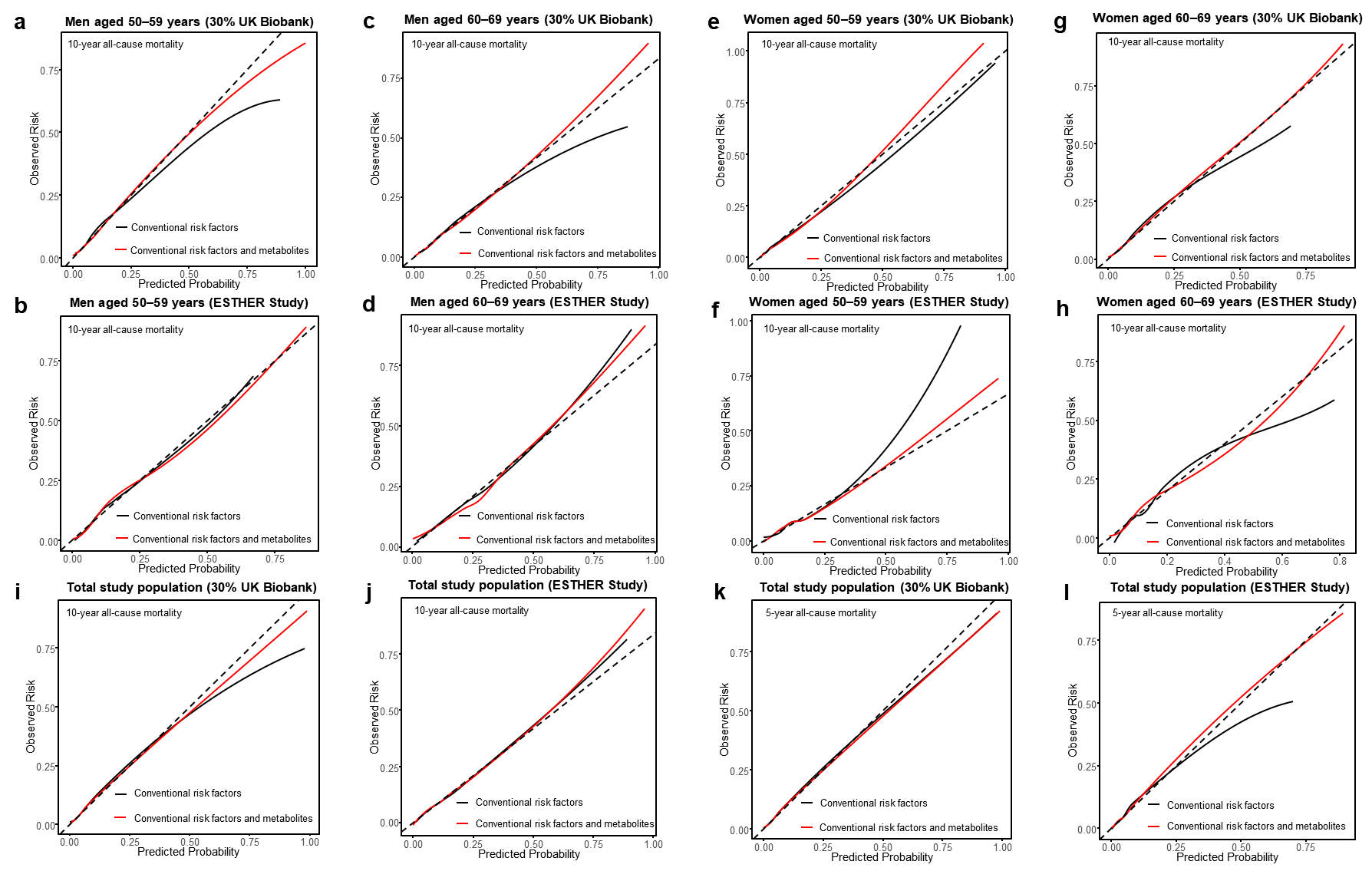


**Figure S6.** **Calibration curves of the models only including conventional risk factors and their combinations with the metabolites for risk prediction in the internal and external validation cohorts.**

Notes: For 10-year all-cause mortality, calibration^[13]^ was assessed in: **a)** younger men (30% UK Biobank), **b)** younger men (ESTHER), **c)** older men (30% UK Biobank), **d)** older men (ESTHER), **e)** younger women (30% UK Biobank), **f)** younger women (ESTHER), **g)** older women (30% UK Biobank), **h)** older women (ESTHER), **i)** the total study population (30% UK Biobank), and **j)** the total study population (ESTHER). For 5-year all-cause mortality, calibration was assessed in **k)** the total study population from 30% of UK Biobank and **l)** the total study population from ESTHER.


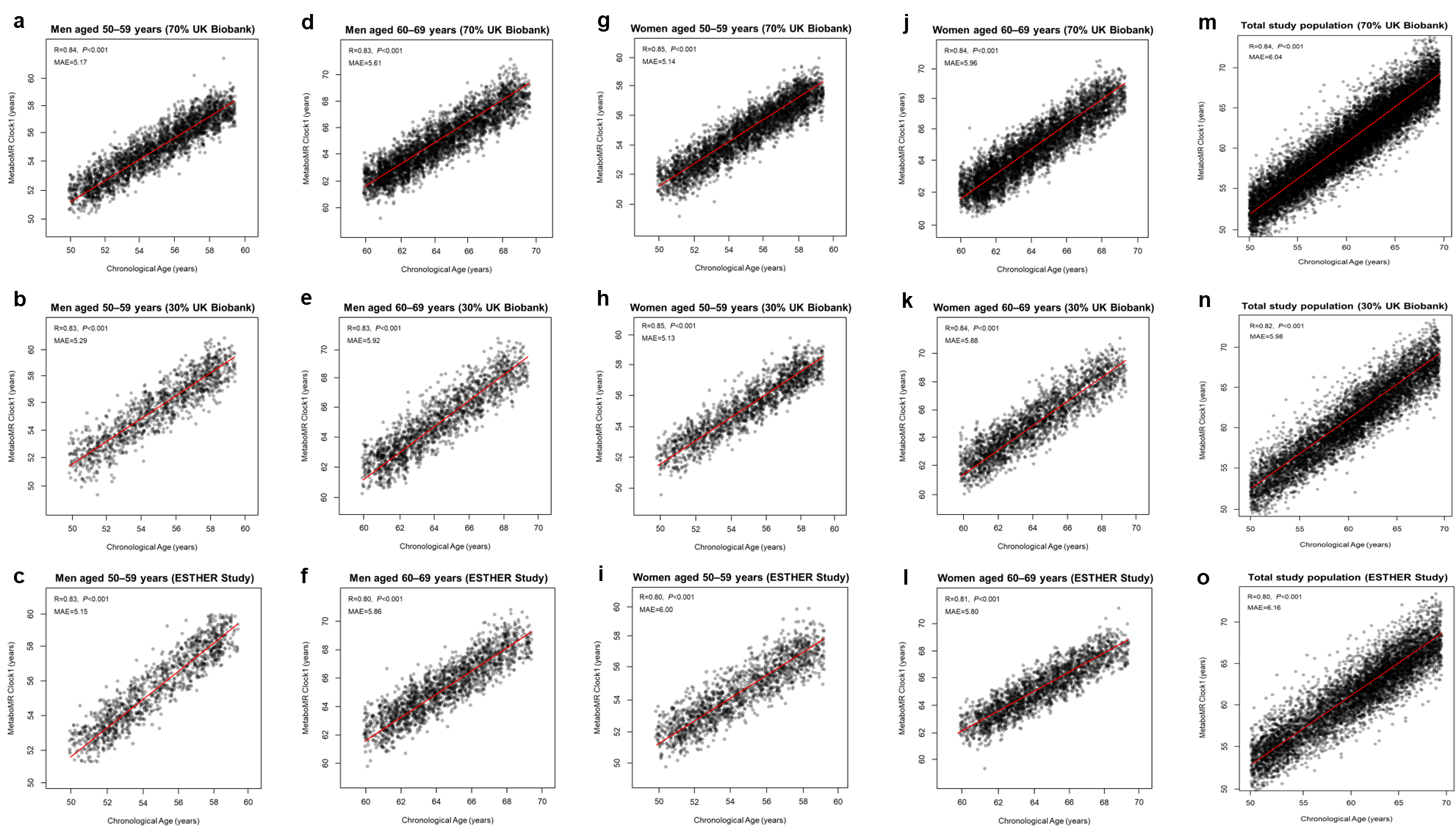


**Figure S7.** **Linear correlation between chronological age and the metabolomic age based on 26 selected metabolomic biomarkers only (metabolomics-based mortality risk clock 1 (MetaboMR clock1).**

Notes: The Pearson correlation coefficient (*r*) is shown in the top left corner of the panels of the figure. In addition, the mean absolute error (MAE) is stated, which reflects the average absolute deviation between predicted metabolomic age and chronological age. Lower MAE values indicate greater model prediction accuracy.^[14-15]^


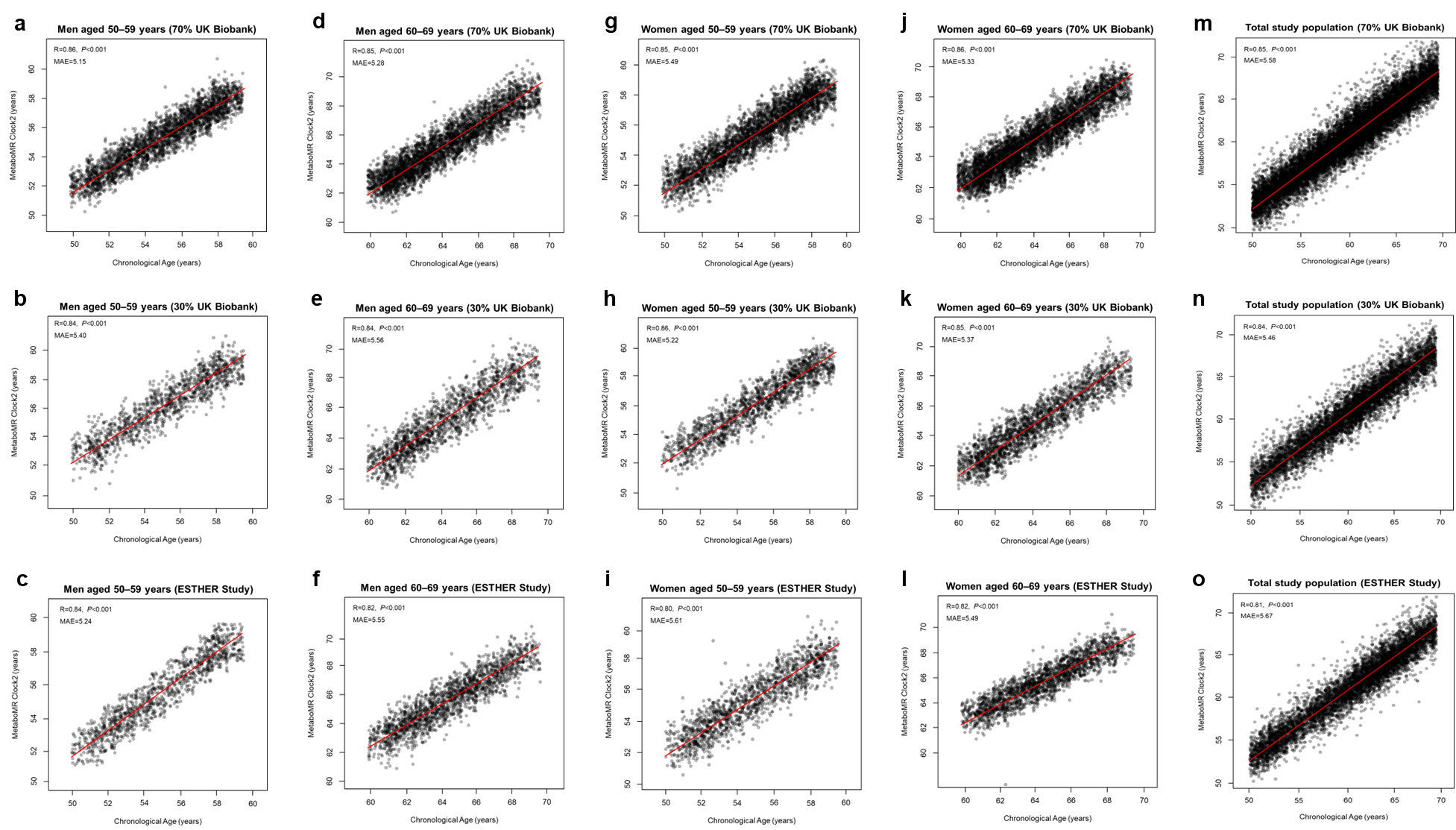


**Figure S8.** **Linear correlation between chronological age and metabolomic age based on 26 selected metabolomic biomarkers and traditional mortality risk factors (metabolomics-based mortality risk clock 2 (MetaboMR clock2)).**

Notes: The Pearson correlation coefficient (*r*) is shown in the top left corner of the panels of the figure. In addition, the mean absolute error (MAE) is stated, which reflects the average absolute deviation between predicted metabolomic age and chronological age. Lower MAE values indicate greater model prediction accuracy.^[14-15]^

**
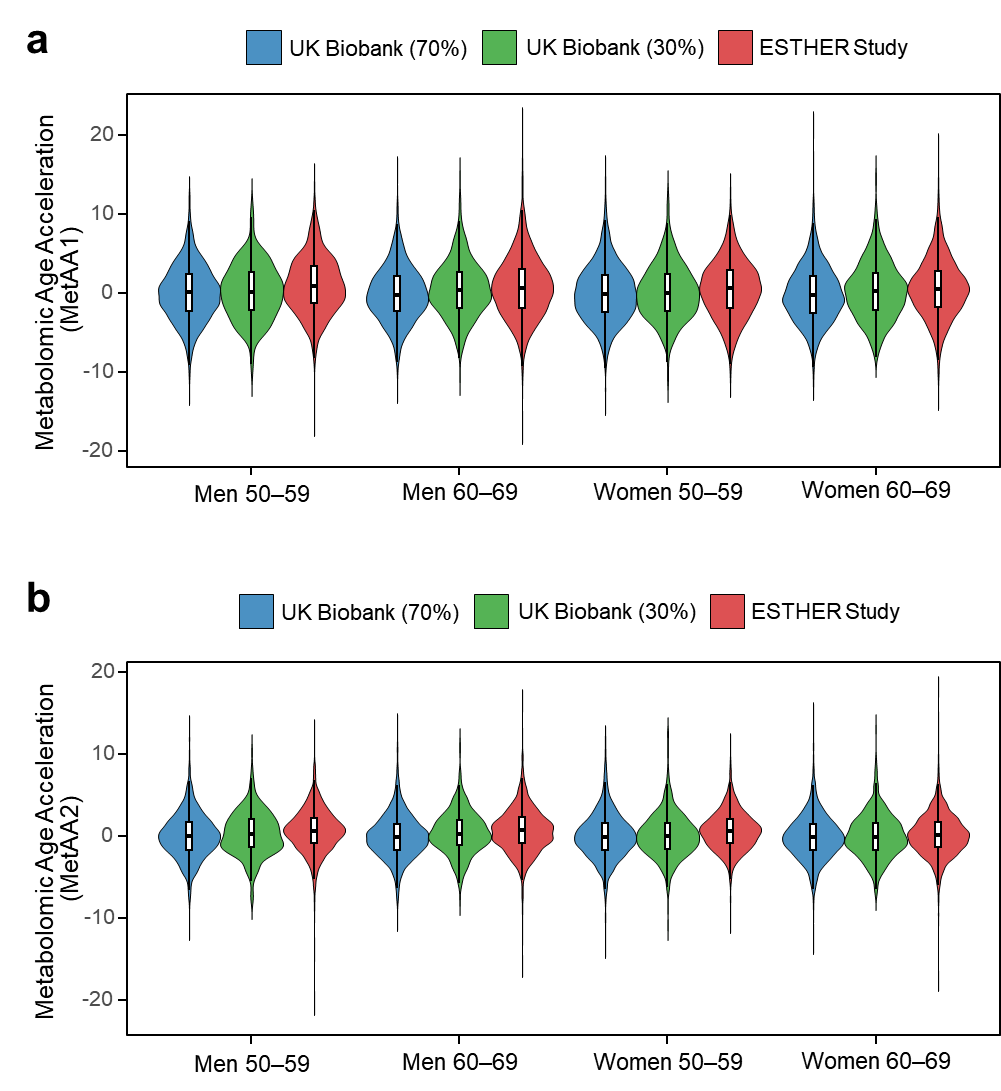
**

**Figure S9.** **Violin plots of the distribution of metabolomic age acceleration (MetAA) derived from (a) MetaboMR clock1 and (b) from MetaboMR clock2 in the UK Biobank and ESTHER cohorts**

**Figure legend:** The center line, box limits, and whiskers represent the median, interquartile range, and full range, respectively.

**Abbreviations:** MetAA, metabolomic age acceleration; MetaboMR, metabolomics-based mortality risk.

**Supplementary References**

[1] A. Sjölander, P. W. Dickman, "Why test for proportional hazards-or any other model assumptions?," *American Journal of Epidemiology* **2024**, *193* (6): 926. <https://doi.org/10.1093/aje/kwae002>.

[2] M. J. Stensrud, M. A. Hernán, "Why test for proportional hazards?," *JAMA* **2020**, *323* (14): 1401. <https://doi.org/10.1001/jama.2020.1267>.

[3] Y. Benjamini, Y. Hochberg, "Controlling the false discovery rate: a practical and powerful approach to multiple testing," *Journal of the Royal Statistical Society Series B-statistical Methodology* **1995**, *57* (1): 289. <https://doi.org/10.1111/j.2517-6161.1995.tb02031.x>.

[4] P. M. Grambsch, T. M. Therneau, "Proportional hazards tests and diagnostics based on weighted residuals," *Biometrika* **1994**, *81* (3): 515.

[5] M. Kanehisa, S. Goto, Y. Sato, M. Kawashima, M. Furumichi, M. Tanabe, "Data, information, knowledge and principle: back to metabolism in KEGG," *Nucleic Acids Research* **2014**, *42* (Database issue): D199. <https://doi.org/10.1093/nar/gkt1076>.

[6] J. Deelen, J. Kettunen, K. Fischer, et al., "A metabolic profile of all-cause mortality risk identified in an observational study of 44,168 individuals," *Nature Communications* **2019**, *10* (1): 3346. <https://doi.org/10.1038/s41467-019-11311-9>.

[7] K. Fischer, J. Kettunen, P. Würtz, et al., "Biomarker profiling by nuclear magnetic resonance spectroscopy for the prediction of all-cause mortality: an observational study of 17,345 persons," *PLoS Medicine* **2014**, *11* (2): e1001606. <https://doi.org/10.1371/journal.pmed.1001606>.

[8] M. Ersbøll, N. Valeur, U. M. Mogensen, et al., "Prediction of all-cause mortality and heart failure admissions from global left ventricular longitudinal strain in patients with acute myocardial infarction and preserved left ventricular ejection fraction," *Journal of the American College of Cardiology* **2013**, *61* (23): 2365. <https://doi.org/10.1016/j.jacc.2013.02.061>.

[9] M. J. Pencina, R. B. D'Agostino, Sr., E. W. Steyerberg, "Extensions of net reclassification improvement calculations to measure usefulness of new biomarkers," *Statistics in Medicine* **2011**, *30* (1): 11. <https://doi.org/10.1002/sim.4085>.

[10] K. F. Kerr, Z. Wang, H. Janes, R. L. McClelland, B. M. Psaty, M. S. Pepe, "Net reclassification indices for evaluating risk prediction instruments: a critical review," *Epidemiology* **2014**, *25* (1): 114. <https://doi.org/10.1097/ede.0000000000000018>.

[11] P. Langfelder, S. Horvath, "WGCNA: an R package for weighted correlation network analysis," *BMC Bioinformatics* **2008**, *9*: 559. <https://doi.org/10.1186/1471-2105-9-559>.

[12] L. Antolini, B. H. Nam, R. B. D'Agostino, "Inference on correlated discrimination measures in survival analysis: a nonparametric approach," *Communications in Statistics-Theory and Methods* **2004**, *33* (9): 2117. <https://doi.org/10.1081/sta-200026579>.

[13] B. Van Calster, D. J. McLernon, M. van Smeden, L. Wynants, E. W. Steyerberg, "Calibration: the Achilles heel of predictive analytics," *BMC Medicine* **2019**, *17* (1): 230. <https://doi.org/10.1186/s12916-019-1466-7>.

[14] X. Jia, J. Fan, X. Wu, et al., "A novel metabolomic aging clock predicting health outcomes and its genetic and modifiable factors," *Advanced Science* **2024**, *11* (43): e2406670. <https://doi.org/10.1002/advs.202406670>.

[15] J. Mutz, R. Iniesta, C. M. Lewis, "Metabolomic age (MileAge) predicts health and life span: a comparison of multiple machine learning algorithms," *Science Advances* **2024**, *10* (51): eadp3743. <https://doi.org/10.1126/sciadv.adp3743>.
